# The Global Research Collaboration of Network Meta-Analysis: A Social Network Analysis

**DOI:** 10.1371/journal.pone.0163239

**Published:** 2016-09-29

**Authors:** Lun Li, Ferrán Catalá-López, Adolfo Alonso-Arroyo, Jinhui Tian, Rafael Aleixandre-Benavent, Dawid Pieper, Long Ge, Liang Yao, Quan Wang, Kehu Yang

**Affiliations:** 1 Department of General Surgery, The Second Xiangya Hospital, Central South University, Changsha, Hunan, China; 2 Evidence-Based Medicine Center, School of Basic Medical Sciences, Lanzhou University, Lanzhou, Gansu, China; 3 Department of Medicine, University of Valencia/ INCLIVA Health Research Institute and CIBERSAM, Valencia, Spain; 4 Clinical Epidemiology Program, Ottawa Hospital Research Institute, Ottawa, ON, Canada; 5 Department of History of Science and Documentation, University of Valencia, Valencia, Spain; 6 Ingenio-Spanish National Research Council (CSIC) and UISYS-University of Valencia, Valencia, Spain; 7 Institute for Research in Operative Medicine, Department for Evidence-Based Health Services Research, Witten/Herdecke University, Cologne, Germany; 8 Department of Gastrointestinal Surgery, Xijing Hospital of Digestive Diseases, Xijing Hospital, Four Military Medical University, Xi'an, Shaanxi, China; University of Texas at Austin, UNITED STATES

## Abstract

**Background and Objective:**

Research collaborations in biomedical research have evolved over time. No studies have addressed research collaboration in network meta-analysis (NMA). In this study, we used social network analysis methods to characterize global collaboration patterns of published NMAs over the past decades.

**Methods:**

PubMed, EMBASE, Web of Science and the Cochrane Library were searched (at 9^th^ July, 2015) to include systematic reviews incorporating NMA. Two reviewers independently selected studies and cross-checked the standardized data. Data was analyzed using Ucinet 6.0 and SPSS 17.0. NetDraw software was used to draw social networks.

**Results:**

771 NMAs published in 336 journals from 3459 authors and 1258 institutions in 49 countries through the period 1997–2015 were included. More than three-quarters (n = 625; 81.06%) of the NMAs were published in the last 5-years. The BMJ (4.93%), Current Medical Research and Opinion (4.67%) and PLOS One (4.02%) were the journals that published the greatest number of NMAs. The UK and the USA (followed by Canada, China, the Netherlands, Italy and Germany) headed the absolute global productivity ranking in number of NMAs. The top 20 authors and institutions with the highest publication rates were identified. Overall, 43 clusters of authors (four major groups: one with 37 members, one with 12 members, one with 11 members and one with 10 members) and 21 clusters of institutions (two major groups: one with 62 members and one with 20 members) were identified. The most prolific authors were affiliated with academic institutions and private consulting firms. 181 consulting firms and pharmaceutical industries (14.39% of institutions) were involved in 199 NMAs (25.81% of total publications). Although there were increases in international and inter-institution collaborations, the research collaboration by authors, institutions and countries were still weak and most collaboration groups were small sizes.

**Conclusion:**

Scientific production on NMA is increasing worldwide with research leadership of Western countries (most notably, the UK, the USA and Canada). More authors, institutions and nations are becoming involved in research collaborations, but frequently with limited international collaborations.

## Introduction

The current landscape of biomedical research is faced with complex challenges which require collaboration among scientists and institutions all over the world. Research collaborations occur when scientists and investigators work together to move their research forward, which contributes to the advancement of knowledge by exploiting the results of scientific efforts more cost-effectively and also makes resources sharing and knowledge stocking possible internationally [1]. Systematic reviews and meta-analyses are the backbone of evidence based medicine which requires conscientious, explicit, and judicious use of current best evidence in making decisions about the care of individual patients [2].

In the last decade, network meta-analysis (NMA) has been introduced as a generalization of pairwise meta-analysis, enabling simultaneous assessment of the relative effectiveness of several interventions across a network of randomized clinical trials (RCTs) [3–5]. The value of NMAs for health-care decision making has been recognized and accepted by different health technology assessment and funding agencies worldwide [6]. NMA requires more complex meta-analytic techniques (that are associated with additional assumptions) when evaluating large numbers of trials, participants and treatment alternatives [7].

Research collaborations in clinical research have evolved over the last decades. For example, a recent social network study showed that there have been an increasing number of research collaborations in the science of meta-analysis published in high-impact journals worldwide [8]. To the best of our knowledge, there has been no specific study focusing on the characterization of global research collaborations on published NMAs. Social network analysis is grounded in the assessment of empirical data and may provide an appropriate approach to identify scientists, groups and institutions [8, 9]. It also offers highly interesting information to understand the nature and structure of relationships and interactions within a scientific community. One frequently used approach for studying research collaboration involves co-authorship networks which are a class of social networks illustrating collaboration based on presence as co-authors in a research publication. Publication data can also be used to explore and visualize research collaboration among institutions and countries [10, 11]. Therefore, we used social network analysis methods to characterize global collaboration patterns of published NMAs over the past decades.

## Method

### Literature searching

PubMed, EMBASE, Web of Science and the Cochrane Library were searched without publication date restrictions. The search strategy was developed based on two publications [12, 13] and reported in a previous study [14]. The search strategy was peer-reviewed prior to execution by BS (20 years of experience as information specialist) and KY (20 years of experience as information specialist) using peer review of electronic search strategies (PRESS) [15] (S1 Appendix). All searches were conducted at 9th July, 2015.

### Inclusion and exclusion criteria

Systematic reviews incorporating NMA were included, no matter what kinds of names they used, such as *mixed treatment comparison*, *indirect comparison meta-analysis*, *multiple treatment meta-analysis*. We considered NMA as any meta-analysis which are analyzing, simultaneously, three or more different interventions in one meta-analysis [16]. A NMA includes adjusted indirect treatment comparison of open-loop networks and mixed treatment comparison of at least one closed loop in the evidence network [6, 7]. We also included full economic evaluations where both costs and health outcomes have been measured if they conducted a NMA for the clinical effectiveness instead of citing a previous NMA. Single technology appraisals or diagnostic accuracy test meta-analyses were excluded. Methodological studies, protocols, letters, comments, editorials, and naive indirect comparison meta-analysis which only used the raw data from included primary studies [17] were also excluded. If a NMA was found repeated in several publications, that latest was included.

### Study selection

Two reviewers (MP, LL and/or LG) independently screened the titles and abstracts to select the potential articles. Then the full-texts were retrieved and read to judge. Disagreements were discussed among the two reviewers.

### Data preparation

We created a bibliometric database in order to prepare the information, including publication titles, the year of publication, the journal title, author’s names, institutional names, country of origin, the number of authors, institutions and countries. For each included paper, one author (LL) abstracted the data based on the full-texts and all information was checked by a second author (LY, JT, QW, or DP).

A process of standardization was carried out to bring together the various different names of a specific author or institution. The criterion followed in the case of the authors was the occurrence of the institutional signature associated with the variations of names and surnames. In the case of institutions, it is important to point out that, in many bibliographic registers, two or more institutions are included under the same institutional address (for example, in the cases of research institutes and/or hospitals attached to universities). In such cases, these institutions were kept apart, giving each bibliographic register as many institutional signatures as macro-institutions can be identified [8]. If there were different forms for the names of the same person or institutions, and the information will be checked based on the publications, information from official websites and PubMed searching.

### Bibliometric indicators and social network analysis

We used the term co-authorship to refer to joint authorship of a scientific paper by 2 individual authors, and institutional collaboration to refer to joint relationship by different institutions and international collaboration to refer to joint relationship by different countries. Threshold of collaboration refers to the figure used to form clusters of authors, institutions and countries (the frequency of co-authorship between pairs of authors or of collaboration between institutions or countries), and reflects a criterion to label identifiable clusters as research groups. A higher threshold of collaboration would guarantee a clear view of the research collaboration networks, and thus center the analyses on the more intense collaboration relationships. We applied an *a posteriori* threshold of three or more papers signed in co-authorship for the main analyses.

We used BICOMS (Bibliographic Item Co-Occurrence Mining System) software to automatically abstract information from the TXT file, and constructed binary matrix of relationships between authors, institutions, and countries. Then it was imported to Ucinet 6.0 program (version 6.198, Analytic Technologies, USA) to construct network data.

We calculated degree of centrality, closeness, betweenness for author, institution and country networks using Ucinet 6.0. Centrality measures help the researcher to determine which nodes are important to be kept in the network [18–20]. Degree of centrality of a node is characterized as the number of ties or edges close to a given node, that is, degree of centrality equals the number of coauthors of a given author [19, 20]. In connected graphs there is a natural distance metric between all pairs of nodes, defined by the length of their shortest paths. The farness of a node is defined as the sum of its distances to all other nodes, and its closeness is defined as the inverse of the farness. Thus, the more central a node is the lower its total distance to all other nodes. Closeness can be regarded as a measure of how long it will take to spread information to all other nodes sequentially. Closeness of centrality deals with the structural position of nodes in the whole network [19]. Betweenness is a centrality measure of a vertex within a graph. This indicator is defined as the number of shortest paths crossing through a node [20]. Betweenness centrality refers to the extent that a person lies in-between two other people that would otherwise not be connected. Betweenness centrality quantifies the number of times a node acts as a bridge along the shortest path between two other nodes. A high ‘betweenness’ score would indicate a person’s potential to act as a gatekeeper of information/resources between the people they connect within a particular network. Individual betweenness centrality scores were generated for ‘provision of information’ relationships to understand whether key individuals were central to this information [21].

We presented network diagrams or 'sociograms' to represent the structure of scientific collaboration within the groups and across groups. A sociogram is a graphic representation of social connections and relationships among members of a scientific community and may be helpful in presenting networks of collaborations as well as the influence of scientists, institutions and countries in the field of NMA. Sociograms are generally composed of two fundamental components: vertices (authors, institutions or countries) and edges (number of papers or collaborations). Specialized software NetDraw was used to draw social networks.

### Data analysis for the basic information

All continuous variables were presented as mean ± SD and median (inter-quartile range, IQR), and categorical variables were expressed using frequencies and percentages. All data was analyzed using SPSS software 17.0.

## Results

### Search results

**Fig 1:**
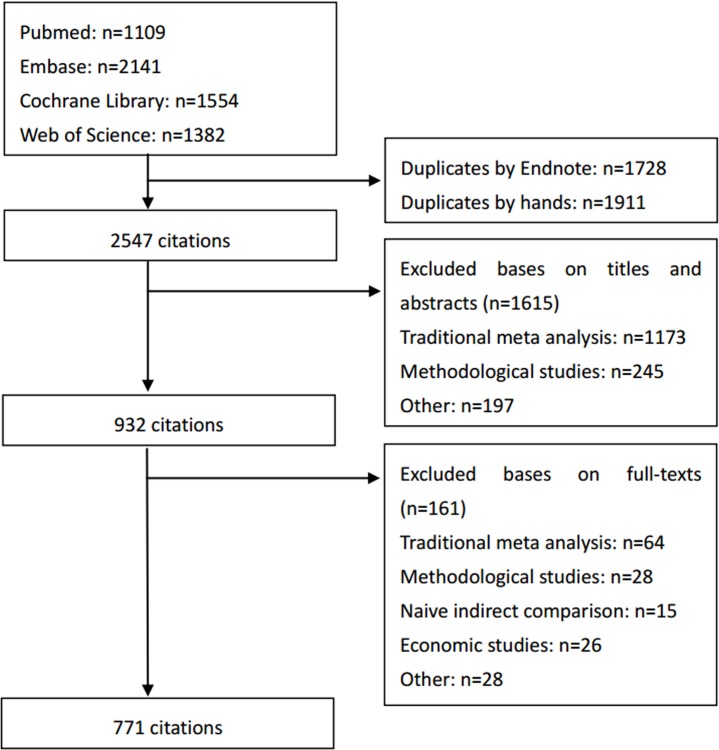
Flow chart. Selection of NMAs.

After literature search, 6168 citations were retrieved. Of them, 3639 citations were duplicates, so 2547 citations were sent for further screening. Based on titles and abstracts, 1615 citations were excluded, as they were traditional meta-analysis (n = 1173), methodological studies (n = 245), or other (n = 197). Then 161 articles were excluded based on reading full-texts, because of traditional meta-analysis (n = 64), methodological studies (n = 28), naive indirect comparison (n = 15), economic studies (n = 26) and other (n = 28). Finally, 771 NMAs were included (Fig 1, S2 Appendix).

### Global publication trend

**Fig 2:**
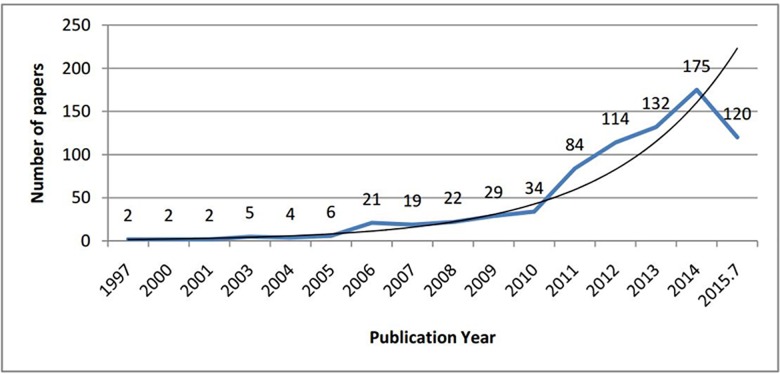
The number of published NMAs from 1997 to 2015. In blue, observed values (number of publications). In black, expected values (prediction line).

The trend of annual papers from 1997 to 2015 is shown in Fig 2. More than three-quarters (n = 625; 81.06%) of the NMAs were published in the last 5-years. The first NMA was an adjusted indirect comparison and published in 1997 [22], and the first mixed treatment comparison was published in 2003 [23]. The number of published NMAs increased slowly until 2010. The year that published most NMAs was 2014 (175/771; 22.70%). Both the number of NMAs conducted by two or more institutions and by international collaboration among different countries also increased in the study period (Fig A in S3 Appendix).

### Journals

336 journals published 771 papers, 211 (62.80%) journals published only one NMA, 56 (16.67%) journals published two, 28 (8.33%) journals published three, and 41 (12.20%) published more than three papers. The BMJ published the largest number of papers (n = 38; 4.93%), followed by Current Medical Research & Opinion (n = 36; 4.67%), Health Technology Assessment (n = 21; 4.0%), and PLOS One (n = 31; 4.02%) (Table 1).

**Table 1:** The journals that published five or more NMAs.

Journal	Publications (%)	Journal	Publications (%)
BMJ	38 (4.93)	JAMA	6 (0.78)
Curr Med Res Opin	36 (4.67)	Ann Rheum Dis	6 (0.78)
PLOS One	31 (4.02)	BMC Med	6 (0.78)
Health Technol Assess	26 (3.37)	Thromb Haemost	6 (0.78)
Cochrane Database Syst Rev	22 (2.85)	J Rheumatol	5 (0.65)
Clin Ther	16 (2.08)	Lancet Oncol	5 (0.65)
Lancet	12 (1.56)	Medicine (Baltimore)	5 (0.65)
Value Health	9 (1.17)	Int J Chron Obstruct Pulmon Dis	5 (0.65)
Ann Intern Med	9 (1.17)	J Clin Periodontol	5 (0.65)
BMJ Open	9 (1.17)	J Clin Endocrinol Metab	5 (0.65)
J Am Coll Cardiol	8 (1.04)	J Clin Pharm Ther	5 (0.65)
Int J Cardiol	8 (1.04)	Diabetes Obes Metab	5 (0.65)
Aliment Pharmacol Ther	8 (1.04)	AHRQ Comparative Effectiveness Reviews	5 (0.65)
QJM	7 (0.91)	Cancer Treat Rev	5 (0.65)

### Authors

The average author number per NMA was 6.52 (SD 3.68, range 1–32, median 6, IQR 4–8) and increased from 1997 to 2014 (Fig B in S3 Appendix). The four-author NMAs were most (113, 14.66%) followed by five-author NMAs (111, 14.40%) and six-author NMAs (105, 13.62%) (Fig C in S3 Appendix).

A total of 3459 authors were retrieved. 2684 (77.59%) authors were involved in only one NMA, 483 (13.96%) authors were involved in two, 129 (3.73%) authors were involved in three, and only 163 (4.71%) authors contributed more than three NMAs. We identified 21 authors who published nine or more papers (Table 2). The most prolific authors were Jeroen P Jansen with 24, Edward J Mills with 21, Giuseppe Biondi-Zoccai with 18, Kristian Thorlund, Yukang Tu, Georgia Salanti, Jos Kleijnen with 13 each. One third of the 22 most prolific authors were affiliated with consulting firms and/or the pharmaceutical and medical technology industries.

**Table 2:** Ranking of most prolific authors (9 or more papers), their affiliations and centrality measures.

Author	Affiliation	Publications (%)	Collaborators	Freeman's degree	Closeness	Freeman betweenness
Jeroen P Jansen	Redwood Outcomes Health Consulting Inc/Precision for Value, Canada and Tufts University, USA	24 (3.11)	31	61	4.01	0.42
Edward J Mills	University of Ottawa, Redwood Outcomes Health Consulting Inc/Precision for Value, Canada and Stanford University, USA	21 (2.72)	28	80	3.62	0.42
Giuseppe Biondi-Zoccai	Sapienza University of Rome, Italy	18 (2.33)	49	144	6.33	0.42
Kristian Thorlund	McMaster University, Redwood Outcomes Health Consulting Inc/Precision for Value, Canada and Stanford University, USA	13 (1.69)	21	50	2.58	0.42
Yukang Tu	National Taiwan University, China Taiwan	13 (1.69)	11	50	1.42	0.13
Georgia Salanti	University of Ioannina, Greece (now, University of Bern, Switzerland)	13 (1.69)	27	57	3.49	0.42
Jos Kleijnen	Kleijnen Systematic Reviews Ltd, UK and University of Maastricht, Netherlands	13 (1.69)	20	40	3.10	0.42
Ping Wu	University of Ottawa and Redwood Outcomes Health Consulting Inc/Precision for Value, Canada	12 (1.56)	21	46	2.71	0.42
Alex Sutton	University of Leicester, UK	12 (1.56)	24	44	3.10	0.42
Eric Druyts	University of Ottawa and Redwood Outcomes Health Consulting Inc/Precision for Value, Canada	11 (1.43)	13	36	1.68	0.42
George Wells	University of Ottawa, Canada	11 (1.43)	20	58	2.58	0.42
Gerald Gartlehner	University of North Carolina at Chapel Hill, USA; Danube University, Krems, Austria	10 (1.30)	21	74	2.71	0.13
Richard A Hansen	University of North Carolina at Chapel Hill, USA and Auburn University, USA	10 (1.30)	21	74	2.71	0.13
Peter Jüni	University of Bern, Switzerland (now, St. Michael’s Hospital, Canada)	10 (1.30)	36	79	465	0.42
Chris Cameron	Canadian Agency for Drugs and Technologies in Health, Canada (now, Cornerstone Research Group Inc., Canada)	10 (1.30)	24	55	3.10	0.42
Mohammad Hassan Murad	Mayo Clinic, USA	10 (1.30)	13	26	1.68	0.42
Alphons G H Kessels	University of Maastricht, The Netherlands	9 (1.17)	14	25	1.81	0.42
Neil Hawkins	ICON plc, Oxford Outcomes Ltd, UK	9 (1.17)	18	21	2.33	0.42
Nicky Welton	University of Bristol, UK	9 (1.17)	19	21	2.46	0.42
Gregg W Stone	Columbia University Medical Center, USA	9 (1.17)	54	133	6.98	0.42
Giacomo Frati	Sapienza University of Rome, Italy and IRCCS Neuro Med, Italy	9 (1.17)	17	60	3.49	0.42
Rob Riemsma	Kleijnen Systematic Reviews Ltd, UK	9 (1.17)	31	32	2.20	0.42

**Fig 3:**
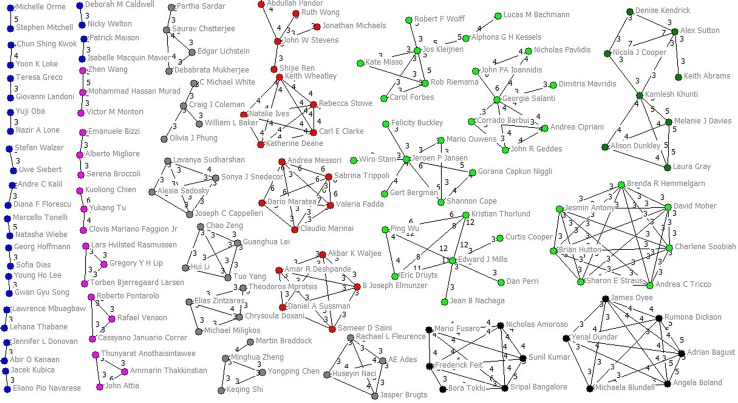
Main clusters of authors (two-eight members) applying a threshold of three or more papers signed in co-authorship.

**Fig 4:**
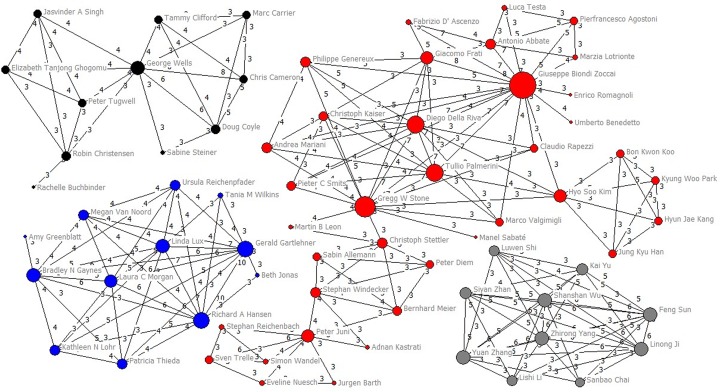
Main clusters of authors (10–37 members) applying a threshold of three or more papers signed in co-authorship.

Among 775 authors who published two NMAs or more, 556 (71.74%) authors collaborated with others twice at most and only 219 (28.26%) authors collaborated with others at least three times (Fig D in S3 Appendix). Only 66 authors (8.52%) collaborated with others at least five times (Fig E in S3 Appendix). The average collaboration times per people they collaborated with was 1.76 (SD 0.57, median 1.75, IQR 1.36–2.00, range 1–5.25). Gregg W Stone (with 54 collaborators) and Giuseppe Biondi Zoccai (with 49) collaborated with most people, followed by Hyo Soo Kim (with 45), Peter Jüni (with 36), Tullio Palmerini (with 34) and Diego Della Riva (with 34) (Table 2). Applying a threshold of three or more papers published as co-authors (Figs 3 and 4), we identified 43 clusters. Of them, four major groups (37, 12, 11 and 10 members for each group) were identified. The top 20 highest degree, closeness, betweenness were listed in Table A in S4 Appendix.

### Institutions

The average institution number per NMA was 3.63 (SD 2.60, range 1–26, median 3, IQR 2–5). The average institution number per NMA fluctuated around three before 2007, and around four after 2007 (Fig B in S3 Appendix). The three-institution NMAs were most (170, 22.05%) followed by two-institution NMAs (154, 19.97%) and four- institution NMAs (129, 16.73%) (Fig C in S3 Appendix).

1258 institutions were involved. 833 (66.22%) institutions were involved in only one paper, 173 (13.75%) institutions were involved in two, 87 (6.92%) institutions were involved in three, and 13.12% authors contributed more than three articles. We identified 21 institutions which published 15 or more NMAs (Table 3). The most prolific institutions were McMaster University with 33, University of Ottawa with 30, University of Birmingham with 27, University of Bristol and University of Toronto with 25 each, Sapienza University of Rome and Mapi Values, USA with 22 each, and Mapi Values, Netherlands with 20.

**Table 3:** Ranking of most prolific institutions (15 or more papers), their country and centrality measures.

Institution Name	Country	Publications (%)	Collaborators	Freeman's degree	Closeness	Freeman betweenness
McMaster University	Canada	33 (4.28)	50	111	5.19	6.11
University of Ottawa	Canada	30 (3.89)	46	112	5.19	5.14
University of Birmingham	UK	27 (3.50)	40	63	5.19	6.32
University of Bristol	UK	25 (3.24)	44	61	5.20	6.13
University of Toronto	UK	25 (3.24)	34	68	5.15	3.95
Sapienza University of Rome	Italy	22 (2.85)	52	106	5.15	5.20
Mapi Values, USA	USA	22 (2.85)	27	57	5.11	2.96
Mapi Values, The Netherlands	The Netherlands	20 (2.59)	23	44	5.09	1.56
Columbia University	USA	19 (2.46)	69	147	5.22	9.81
University of Sheffield	UK	19 (2.46)	20	23	5.08	1.81
Harvard University	USA	19 (2.46)	57	78	5.22	8.00
University of York	UK	17 (2.20)	18	24	5.02	1.24
University of Leicester	UK	17 (2.20)	19	23	5.09	1.74
Tufts University	USA	17 (2.20)	28	48	5.17	2.90
Johns Hopkins University	USA	16 (2.08)	23	35	5.10	1.68
University College London	UK	16 (2.08)	27	28	5.14	2.95
University of Alberta	Canada	16 (2.08)	18	40	5.08	0.52
University of Ioannina	Greece	15 (1.95)	29	48	5.13	2.44
Technische Universität München	Germany	15 (1.95)	54	89	5.18	5.02
Maastricht University	Netherlands	15 (1.95)	22	38	5.12	1.35
Mayo Clinic	USA	15 (1.95)	20	31	5.08	1. 27

**Fig 5:**
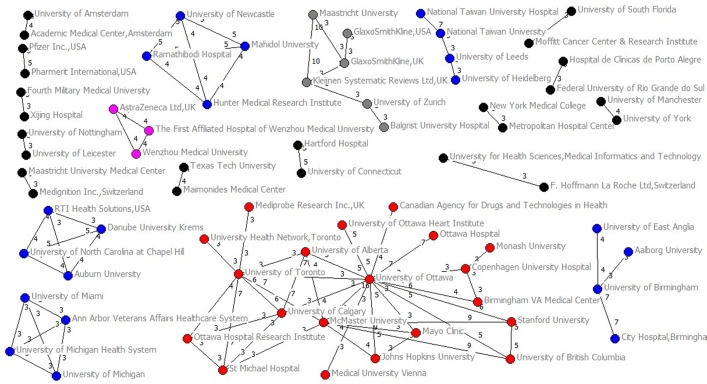
Main clusters of institutions (2–20 members) applying a threshold of three or more papers signed.

**Fig 6:**
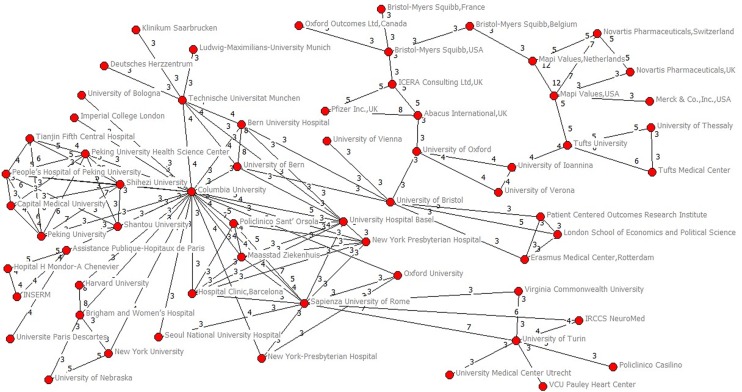
Main clusters of institutions (62 members) applying a threshold of three or more papers signed.

Among 425 institutions which published two NMAs or more, 290 (68.24%) institutions collaborated with others twice at most and 135 (31.76%) institutions collaborated with others at least three times (Fig F in S3 Appendix). Only 57 institutions (13.41%) collaborated with others at least five times (Fig G in S3 Appendix). The average collaboration times per institution they collaborated with was 1.46 (SD 0.56, median 1.33, IQR 1.00–1.71, range 1–5). Columbia University (with 69 institutional collaborators) had the most collaborated institutions, followed by Harvard University (with 57), Technische Universität München (with 54), Sapienza University of Rome (with 52) and McMaster University (with 50) (Table 3). Applying a threshold of three or more NMAs published (Figs 5 and 6), we identified 21 clusters. Of them, two major groups (62 and 20 members for each group) were identified. The top 20 highest degree, closeness, betweenness were listed in Table B in S4 Appendix.

### Country

The average country number per NMA was 1.84 (SD 1.36, range 1–13, median 1, IQR 1–2). The average country number per NMA fluctuated around one before 2007, and around two after 2007 (Fig B in S3 Appendix). The one-country NMAs were most (423, 54.86%) followed by two-country NMAs (185, 23.99%) and three-country NMAs (78, 10.12%) (Fig C in S3 Appendix).

49 countries were involved in conducting and publishing NMA. Twelve (24.49%) countries were involved in only one NMA, five (10.20%) countries were involved in two NMAs, three (6.12%) countries were involved in three NMAs, and 59.12% countries contributed more than three articles. We identified 12 countries which published 30 or more papers (Table 4). The UK (with 289 publications; 37.48%) and USA (with 281; 36.45%) published the most NMAs, followed by Canada (with 110; 14.27%) and China (with 87; 11.28%). The top 20 highest degree, closeness, betweenness were listed in Table C in S4 Appendix.

**Table 4:** Ranking of most prolific countries (10 or more papers) and their centrality measures.

Country	Publications (%)	Single country publications (%)	Collaborative publications (%)	Country collaborators	Freeman'sdegree	Closeness	Freeman betweenness
UK	289 (37.48)	108 (37.37)	181 (62.63)	31	386	47.37	16.09
USA	281 (36.45)	99 (35.23)	182 (64.77)	30	376	46.75	13.24
Canada	110 (14.27)	36 (32.73)	74 (67.27)	23	156	42.86	8.20
China	87 (11.28)	62 (71.26)	25 (28.74)	14	43	38.30	0.61
Netherlands	84 (10.89)	5 (5.95)	79 (94.05)	20	209	41.38	1.11
Italy	73 (9.47)	19 (26.03)	54 (73.97)	24	173	43.37	5.46
Germany	68 (8.82)	10 (14.71)	58 (85.29)	24	172	43.37	3.60
France	52 (6.74)	15 (28.85)	37 (71.15)	20	124	40.911	1.50
Switzerland	51 (6.61)	4 (7.84)	47 (92.16)	19	135	40.45	1.09
Australia	33 (4.28)	9 (27.27)	24 (72.73)	20	73	41.38	1.51
Belgium	32 (4.15)	2 (6.25)	30 (93.75)	17	87	40	1.92
Denmark	30 (3.89)	2 (6.67)	28 (93.33)	17	89	40	1.62
Greece	26 (3.37)	2 (7.69)	24 (92.31)	14	54	38.71	0.47
Spain	23 (2.98)	6 (26.09)	17 (73.91)	19	83	40.45	1.10
Brazil	22 (2.85)	8 (36.36)	14 (63.64)	16	45	39.13	1.18
Korea	21 (2.72)	9 (42.86)	12 (57.14)	18	54	40	0.69
Austria	18 (2.33)	2 (11.11)	16 (88.89)	12	39	37.90	0.73
Sweden	13 (1.69)	1 (7.69)	12 (92.31)	14	39	38.71	5.68
Japan	12 (1.56)	3 (25.00)	9 (75.00)	10	20	36.36	0.04
Poland	10 (1.30)	1 (10.00)	9 (90.00)	15	29	39.13	1.29

**Fig 7:**
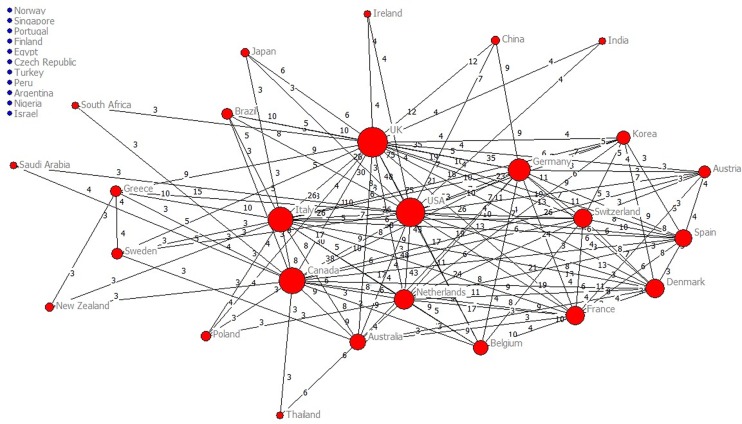
Global collaboration. Main clusters of countries applying a threshold of three or more papers signed.

Applying a threshold of three or more papers published (Fig 7), we identified one cluster of countries with research leadership of Western countries (most notably, from Europe and North America). The USA and the UK published the largest number of international collaboration publications (182 and 181, respectively), followed by the Netherlands (with 79) and Canada (with 74). Among the most prolific countries, China had the largest number of single country papers (Table 4).

### Consulting firm or pharmaceutical industry involvement

Many consulting firms or pharmaceutical industries were involved in conducting and publishing NMAs. There were 181 sub-companies (106 patient companies) accounting for 14.39% of all institutions, and they conducted or involved in 25.68% NMAs (Table 5).

**Table 5:** The ranking of consulting firm or pharmaceutical industry.

According to sub-company	Publications (%)	According to parent company	Publications (%)
Mapi Values, USA	22 (2.85)	Mapi Values	31 (4.02)
Mapi Values, Netherlands	19 (2.46)	Pfizer Inc.	26 (3.37)
Pfizer Inc., USA	14 (1.82)	Novartis Pharmaceuticals	16 (2.08)
Kleijnen Systematic Reviews Ltd, UK	13 (1.69)	Bristol-Myers Squibb	15 (1.95)
Pfizer Inc., UK	13 (1.69)	AstraZeneca Ltd	13 (1.69)
Novartis Pharmaceuticals, Switzerland	12 (1.56)	RTI Health Solutions	13 (1.69)
Bristol-Myers Squibb, USA	10 (1.30)	Kleijnen Systematic Reviews Ltd	13 (1.69)
Abacus International, UK	10 (1.30)	Oxford Outcomes Ltd	11 (1.43)
Merck & Co.,Inc., USA	9 (1.17)	Merck & Co., Inc.	9 (1.17)
AstraZeneca Ltd, UK	9 (1.17)	F. Hoffmann La Roche Ltd	8 (1.04)
Oxford Outcomes Ltd, UK	9 (1.17)	Evidera	7 (0.91)
Boehringer Ingelheim Ltd, Germany	8 (1.04)	Eli Lilly and Company Ltd	7 (0.91)
Novartis Pharmaceuticals, UK	7 (0.91)	GlaxoSmithKline	6 (0.78)
RTI Health Solutions, USA	7 (0.91)	ICERA Consulting Ltd	6 (0.78)
RTI Health Solutions, UK	7 (0.91)	Janssen Pharmaceutical	5 (0.65)
ICERA Consulting Ltd, UK	6 (0.78)	Pharmerit International	5 (0.65)
GlaxoSmithKline, UK	6 (0.78)	Mediprobe Research Inc.	4 (0.52)
Eli Lilly and Company Ltd, USA	6 (0.78)	UCB Pharma	4 (0.52)
Bristol-Myers Squibb, Belgium	5 (0.65)	Heron Health	4 (0.52)
Evidera, USA	5 (0.65)	Analysis Group Inc.	4 (0.52)
Pharmerit International, USA	5 (0.65)	Ghement Statistical Consulting	4 (0.52)
		OptumInsight	4 (0.52%)

## Discussion

This study summarized some significant collaborations and research trends in published NMAs worldwide. In particular, the social network analysis methodology allowed us to identify the most productive authors and institutions, as well as the identification of the clusters or groups of authors with intense collaboration and the relationships established between the institutions that have published papers in the main biomedical journals through the period 1997–2015. In our study, the UK and the USA headed the absolute productivity ranking (number of papers) followed by Canada, China and other European countries. Although these countries lead in the number of published NMAs, the efforts during the period of study were global, with 771 publications from more than 3400 authors and 1200 institutions in almost 50 different countries.

Research collaboration simply put, is the working together of scientists and researchers to achieve a common goal of producing scientific knowledge [24]. Nonetheless, technology development weakened link between location and scientific research [25] and make global research collaboration possible, and then authors from different locations (institutions or countries) could work together to address particular scientific problems in innovative and effective ways. A NMA has been considered as the next generation of evidence synthesis methods, and research collaboration in NMA has many benefits. First, the number and types of roles and responsibilities in a NMA project are determined by the complexity, purpose and scope of the research question. NMA involves a research protocol, literature searching, study selection, critical appraisal and advanced evidence synthesis methods. This means in order to conduct a rigorous and replicable NMA, at least a methodologist responsible for study design, a librarian responsible for developing search strategy and conducting searching and an experienced analyst responsible for quantitative data synthesis and interpretation of results are needed in the collaboration team. It is rare that one person could do all work related to NMA. In our study, only 1.30% NMAs were conducted by one author. Second, research collaboration can increase value and reduce the waste in research [26–28], also in NMA. The research and medical resources are limited, which requires scientists and researchers all over the world to work together to resolve important clinical and research questions and to establish research priorities. Multidisciplinary teams of experienced researchers can help to enhance the design, conduct, and reporting of NMA that is needed to provide reliable results. Third, collaboration among different institutions or countries also increases the visibility and impact of research. This is obvious when research collaboration occurs among different countries and regions. Systematic reviews with NMA can be crucial for helping scientists, clinicians and other users make sense of vast numbers of new and often conflicting studies comparing multiple interventions that are published in the literature. Research collaboration could prompt NMA to include relevant studies from different countries and overcome the language obstacle, and further to conclude based on much more comprehensive and complete data. Although most time, we make decisions for local use, we need to gather and summarize all available evidence on a particular question globally.

The fact that geographical proximity, as well as language and cultural similarities between countries, international mobility of human capital, may have impact on the way scientists and researchers in different countries collaborate [8, 29]. The analysis of the social structure of the collaborations allowed us to observe a ‘same country or institution phenomenon’, which might be because that scientists and researchers in same country or institution have the quickest and easiest ways to communicate, and further form collaboration relationship. The authors from same institutions tend to have more collaboration times than those authors from different institutions. For example, a ten-member group from China that involved scientists from Peking University, Tianjin Fifth Central Hospital, Capital Medical University an eight-member group from University of Leicester, UK. To our knowledge, this is not occasional, and we could find examples of other groups from same institution or country (a six-author group from University of Liverpool, UK; a five-author group from University of Sheffield, UK, and from Newcastle University, UK). The reasons for ‘same country or institution phenomenon’ are complex. Some countries, particularly China in the last years, have developed a research culture that places a strong emphasis on the production of systematic reviews and meta-analyses [30]. However, it was said cultural history holds back Chinese research collaboration [31]. Other reasons include finance problems, communications, strong competitions, etc. For the UK, the USA and Canada, we could also find same phenomenon, but of a different nature. This might be explained, at least in part, by current research funding opportunities for scientists, research teams and national networks of excellence developing and applying NMA methodologies, but also the strong commitment by some funding agencies and decision makers (for example, the Health Technology Assessment program of the UK National Institute for Health Research, the Evidence-based Practice Center program of the US Agency for Healthcare Research and Quality and the Drug Safety and Effectiveness Network of the Canadian Institutes of Health Research).

A recent paper examined the global collaborative patterns on meta-analyses of randomized trials published in high impact factor medical journals [8]. Although not directly comparable with the present analysis, we have observed similar patterns in terms of size and numbers of clusters for authors, institutions and countries, with the USA, the UK and Canada taking leadership, and a clear under-representation of scientists and institutions based in low and middle income countries (for example, Central and South America, South and East Asia, and Africa). Remarkably, the scientific community captured by that network analysis was centered on a nucleus of prolific authors from prestigious academic centers and affiliated hospitals (for example, the University of Oxford in the UK, McMaster University in Canada, and the University of Bern in Switzerland). Unlike previous research [8], in our study we observed a significant involvement of consulting firms and/or pharmaceutical industry in conducting and publishing NMAs. In particular, an important number of the most prolific authors were affiliated with consulting firms. Similarly, the scientific community captured by the social network analysis identified clusters of authors from private-for-profit companies (consulting firms and the pharmaceutical industry) and affiliated collaborators from academia and hospitals. There are several possible explanations for these findings. Industry funds an increasing proportion of medical research [32]. NMA is the next generation of evidence synthesis methods and an important tool in comparative effectiveness research, which aims to assist consumers, clinicians, purchasers, and policy makers to make informed decisions by providing improved evidence [33]. NMA provides a global estimate of treatment effects for a set of multiple competing interventions, and are becoming increasingly attractive as they offer a comprehensive framework for decision-making [5, 34]. Therefore, industry-funded NMAs may be part of promotional strategies aimed at demonstrating that new products are more effective and at enhancing market access activities. Additionally, a growing body of literature has been drawing attention to the fact that industry-sponsored studies are more likely to report results and conclusions favoring the sponsor’s product or to report more favorable results than non-industry sponsored studies [35–38].

To our knowledge, this study is the first one that aimed to characterize global patterns of collaboration in NMA applying techniques from social network analysis. The present study has identified collaborations between authors and institutions from different countries (which can therefore be considered the scientists and researchers in the vanguard of scientific development within the area), and provides considerable information on the structure that can be put to various purposes, such as: 1) designing and evaluating strategies to promote the efficient use of existing guidelines and methodological standards [6, 7, 39, 40] to improve the methodological quality, transparency, and consistency of study conduct and reporting of NMAs, 2) promoting educational and training activities, 3) sharing knowledge for developing innovative methodologies, and 4) encouraging coordinated research programs for perceived high-priority topics to reduce the burden of diseases and risk factors worldwide [27, 41, 42]. Similarly, we identified an important number of consulting firms or pharmaceutical industries that were involved in publishing NMAs. Whether sponsorship affects or not research collaboration, but also the reporting quality of methods and results, should be considered and explored further in future evaluations of potential sponsorship bias in the field of NMA.

There are also some limitations to our study. First, only English literatures were included, this might introduce potential selection bias for this study and, therefore, findings may not generalizable to NMAs published in a language other than English. Second, we restricted our analysis to NMAs applied to the evaluation of three or more healthcare interventions, but excluded other important publications that also merit consideration in the field (for example, protocols, conceptual and methodological papers). Therefore, there may be researchers and scientists (or institutions) who do not appear or they might be underrepresented. Third, previous research [12, 13, 43–45] has pointed out that important deficiencies in the current reporting of methods and results of published NMAs, but we did not analyze how research collaboration affect the conduct and the reporting quality of NMAs. Fourth, as in many bibliometric analyses using publication data [8], the importance of normalizing the names of authors and their institutions is fundamental for avoiding potential errors. For authors affiliated with two or more different institutions and countries, we opted to assign as many names to the macro-institutions as could be identified. Although this resulted in the problem of multiplying the number of institutions in the recount, it was necessary in order to avoid losing information concerning the macro-institutions occurring in second place or later in the list of names, but it might result in an overrepresentation of inter-institutional collaboration.

## Conclusion

Scientific production on NMA is increasing worldwide with research leadership of Western countries (most notably, European countries, the USA and Canada) and China. More authors, institutions and countries are becoming involved in research collaborations, but frequently with limited international collaborations. The information presented in this study might be helpful for researchers to understand the collaboration networks, identify leading authors and institutions that might promote future global research collaboration practices in NMA and defining a high-priority scientific agenda for comparative effectiveness research.

## Supporting Information

S1 Appendix Search strategy. (DOCX)

S2 Appendix List of included studies. (DOC)

S3 Appendix Additional tables. (DOCX)

S4 Appendix Additional figures. (DOCX)

S5 Appendix PRISMA checklist. (DOC)
