# Supplementary material for: The Global Research Collaboration of Network Meta-Analysis: A Social Network Analysis
Source: PLoS One. 2016 Sep 29;11(9):e0163239. doi: 10.1371/journal.pone.0163239 (PMC5042468; doi:10.1371/journal.pone.0163239)
Supplement: S1 Appendix — (DOCX) [file pone.0163239.s001.docx]

**Appendix S1. Search strategy.**

We searched PubMed (via MEDLINE/PubMed.com), Embase (via Embase.com), Web of Science (via ISI Web of Knowledge) and the Cochrane library (including Cochrane Database of Systematic Reviews, Database of Abstracts of Reviews of Effects, Health Technology Assessment Database, NHS Economic Evaluation Database) from inception to the search time. The search strategy for this study was constructed by using a combination of MESH subject headings and text words relating to “Network meta analysis” OR “mixed treatment comparisons meta analysis” OR “multiple treatments meta analysis” OR “indirect comparison meta analysis”. All the searches were conducted at 9th July, 2015 No restrictions in languages, publication date, and published statuses were applied to database searches.

**PubMed search strategy**

#1 (Network meta analysis [Title/Abstract]) OR Network meta analyses [Title/Abstract]

#2 (meta analysis [Title/Abstract]) OR meta analyses [Title/Abstract]

#3 "Meta-Analysis as Topic"[Mesh] OR "Meta-Analysis" [Publication Type]

#4 #2 OR #3

#5 (mixed treatment*[Title/Abstract]) OR multiple treatment*[Title/Abstract]

#6 #5 AND #4

#7 (((multiple treatment comparison [Title/Abstract]) OR multiple treatment comparisons [Title/Abstract]) OR mixed treatment comparisons [Title/Abstract]) OR mixed treatment comparison [Title/Abstract]

#8 (indirect comparison [Title/Abstract]) OR indirect comparisons [Title/Abstract]

#9 #8 and #4

#10 #1 OR #6 OR #7 OR #9

#8 limit #7 to human

**Embase search strategy**

#1 'network meta analysis' OR 'network meta analyses'

#2 'meta analysis' OR 'meta analyses'

#3 'meta analysis'/exp

#4 #2 OR #3

#5 'mixed treatment' OR 'mixed treatments'

#6 'multiple treatment' OR 'multiple treatments'

#7 #5 OR #6

#8 #7 AND #4

#9 'multiple treatment comparison' OR 'multiple treatment comparisons' OR 'mixed treatment comparisons' OR 'mixed treatment comparison'

#10 'indirect comparison' OR 'indirect comparisons'

#11 #10 and #4

#12 #1 OR #8 OR #9 OR #11

**ISI Web of Knowledge search strategy**

#1 Topic= “Network meta analysis” OR “Network meta analyses”

#5 Topic= “mixed treatment comparison meta analysis” OR “mixed treatment comparisons meta analyses” OR “mixed treatment meta analysis” OR “mixed treatment meta analyses” OR “mixed treatment comparisons” OR “mixed treatment comparison”

#6 Topic= “multiple treatment comparison meta analysis” OR “multiple treatment comparisons meta analyses” OR “multiple treatments meta analysis” OR “multiple treatments meta analyses” OR “multiple treatment meta analysis” OR “multiple treatment meta analyses” OR “multiple treatment comparison” OR “multiple treatment comparisons”

#7 Topic= “indirect comparison meta analysis” OR “indirect comparison meta analyses” OR “indirect comparisons meta analysis” OR “indirect comparisons meta analyses”

#7 #1 OR #4 OR #5 OR #6

**The Cochrane Library search strategy**

#1 Network meta analysis or Network meta analyses: ti,ab,kw (Word variations have been searched)

#2 meta analysis: ti,ab,kw or meta analyses: ti,ab,kw (Word variations have been searched)

#3 MeSH descriptor: [Meta-Analysis as Topic] explode all trees

#4 MeSH descriptor: [Meta-Analysis] explode all trees

#5 #2 or #3 or #4

#6 mixed treatment or mixed treatments (Word variations have been searched)

#7 'multiple treatment or multiple treatments':ti,ab,kw (Word variations have been searched)

#8 #7 or #6

#9 #8 and #5

#10 'multiple treatment comparison' or 'multiple treatment comparisons' or 'mixed treatment comparisons' or 'mixed treatment comparison':ti,ab,kw (Word variations have been searched)

#11 #10 and #5

#12 'indirect comparison or indirect comparisons':ti,ab,kw (Word variations have been searched)

#13 #11 and #5

#14 #1 or #9 or #11 or #13

#15 limit #10 to Cochrane review

#16 limit #10 to other review

#17 limit #10 to Technology Assessments

#18 limit #10 to Economic Evaluations

#18 #15 or #16 or #17
