# Supplementary material for: The Global Research Collaboration of Network Meta-Analysis: A Social Network Analysis
Source: PLoS One. 2016 Sep 29;11(9):e0163239. doi: 10.1371/journal.pone.0163239 (PMC5042468; doi:10.1371/journal.pone.0163239)
Supplement: S2 Appendix — (DOC) [file pone.0163239.s002.doc]

**Appendix 2. List of included studies.**

1. Bucher HC, Guyatt GH, Griffith LE, Walter SD. The results of direct and indirect treatment comparisons in meta-analysis of randomized controlled trials. *J Clin Epidemiol* 1997; 50(6): 683-91.

2. Li Wan Po A, Zhang WY. Systematic overview of co-proxamol to assess analgesic effects of addition of dextropropoxyphene to paracetamol. *BMJ (Clinical research ed)* 1997; 315(7122): 1565-71.

3. Einarson TR, Kulin NA, Tingey D, Iskedjian M. Meta-analysis of the effect of latanoprost and brimonidine on intraocular pressure in the treatment of glaucoma. *Clin Ther* 2000; 22(12): 1502-15.

4. Wilhelmus KR. The treatment of herpes simplex virus epithelial keratitis. *Transactions of the American Ophthalmological Society* 2000; 98: 505-32.

5. Bhandari M, Guyatt GH, Swiontkowski MF, Schemitsch EH. Treatment of open fractures of the shaft of the tibia. *The Journal of bone and joint surgery British volume* 2001; 83(1): 62-8.

6. Sauriol L, Laporta M, Edwardes MD, Deslandes M, Ricard N, Suissa S. Meta-analysis comparing newer antipsychotic drugs for the treatment of schizophrenia: Evaluating the indirect approach. *Clinical Therapeutics* 2001; 23(6): 942-56.

7. Coomarasamy A, Knox EM, Gee H, Song F, Khan KS. Effectiveness of nifedipine versus atosiban for tocolysis in preterm labour: a meta-analysis with an indirect comparison of randomised trials. *BJOG : an international journal of obstetrics and gynaecology* 2003; 110(12): 1045-9.

8. Hind D, Calvert N, McWilliams R, et al. Ultrasonic locating devices for central venous cannulation: meta-analysis. *BMJ (Clinical research ed)* 2003; 327(7411): 361.

9. Hochberg MC, Tracy JK, Hawkins-Holt M, Flores RH. Comparison of the efficacy of the tumour necrosis factor alpha blocking agents adalimumab, etanercept, and infliximab when added to methotrexate in patients with active rheumatoid arthritis. *Ann Rheum Dis* 2003; 62 Suppl 2: ii13-6.

10. Lim E, Ali Z, Ali A, et al. Indirect comparison meta-analysis of aspirin therapy after coronary surgery. *BMJ (Clinical research ed)* 2003; 327(7427): 1309.

11. Psaty BM, Lumley T, Furberg CD, et al. Health outcomes associated with various antihypertensive therapies used as first-line agents: a network meta-analysis. *Jama* 2003; 289(19): 2534-44.

12. Habib AS, El-Moalem HE, Gan TJ. The efficacy of the 5-HT3 receptor antagonists combined with droperidol for PONV prophylaxis is similar to their combination with dexamethasone: a meta-analysis of randomized controlled trials (Structured abstract). *Canadian Journal of Anesthesia*, 2004. http://onlinelibrary.wiley.com/o/cochrane/cldare/articles/DARE-12004009844/frame.html (accessed.

13. Jones L, Griffin S, Palmer S, Main C, Orton V, Sculpher M. Clinical effectiveness and cost-effectiveness of clopidogrel and modified-release dipyridamole in the secondary prevention of occlusive vascular events: a systematic review and economic evaluation (Structured abstract). *Health Technology Assessment Database*, 2004. http://onlinelibrary.wiley.com/o/cochrane/clhta/articles/HTA-32004000809/frame.html (accessed.

14. Panidou ET, Trikalinos TA, Ioannidis JP. Limited benefit of antiretroviral resistance testing in treatment-experienced patients: a meta-analysis. *AIDS (London, England)* 2004; 18(16): 2153-61.

15. Wehren LE, Hosking D, Hochberg MC. Putting evidence-based medicine into clinical practice: comparing anti-resorptive agents for the treatment of osteoporosis. *Curr Med Res Opin* 2004; 20(4): 525-31.

16. Batterham MJ. Investigating heterogeneity in studies of resting energy expenditure in persons with HIV/AIDS: a meta-analysis. *The American journal of clinical nutrition* 2005; 81(3): 702-13.

17. Biondi-Zoccai GG, Agostoni P, Abbate A, et al. Adjusted indirect comparison of intracoronary drug-eluting stents: evidence from a metaanalysis of randomized bare-metal-stent-controlled trials. *Int J Cardiol* 2005; 100(1): 119-23.

18. Brophy JM, Joseph L. Medical decision making with incomplete evidence - Choosing a platelet glycoprotein IIbIIIa receptor inhibitor for percutaneous coronary interventions. *Medical Decision Making* 2005; 25(2): 222-8.

19. Mudge MA, Davey PJ, Coleman KA, et al. A comparison of olanzapine versus risperidone for the treatment of schizophrenia: a meta-analysis of randomised clinical trials (Structured abstract). *International Journal of Psychiatry in Clinical Practice*, 2005. http://onlinelibrary.wiley.com/o/cochrane/cldare/articles/DARE-12005009676/frame.html (accessed.

20. Otoul C, Arrigo C, van Rijckevorsel K, French JA. Meta-analysis and indirect comparisons of levetiracetam with other second-generation antiepileptic drugs in partial epilepsy. *Clinical neuropharmacology* 2005; 28(2): 72-8.

21. Richy F, Schacht E, Bruyere O, Ethgen O, Gourlay M, Reginster JY. Vitamin D analogs versus native vitamin D in preventing bone loss and osteoporosis-related fractures: a comparative meta-analysis. *Calcified tissue international* 2005; 76(3): 176-86.

22. Abou-Setta AM. Firm embryo transfer catheters for assisted reproduction: a systematic review and meta-analysis using direct and adjusted indirect comparisons. *Reproductive biomedicine online* 2006; 12(2): 191-8.

23. Berner MM, Kriston L, Harms A. Efficacy of PDE-5-inhibitors for erectile dysfunction. A comparative meta-analysis of fixed-dose regimen randomized controlled trials administering the International Index of Erectile Function in broad-spectrum populations. *International journal of impotence research* 2006; 18(3): 229-35.

24. Brown TJ, Hooper L, Elliott RA, et al. A comparison of the cost-effectiveness of five strategies for the prevention of non-steroidal anti-inflammatory drug-induced gastrointestinal toxicity: a systematic review with economic modelling. *Health technology assessment (Winchester, England)* 2006; 10(38): iii-iv, xi-xiii, 1-183.

25. Chou R, Fu R, Huffman LH, Korthuis PT. Initial highly-active antiretroviral therapy with a protease inhibitor versus a non-nucleoside reverse transcriptase inhibitor: discrepancies between direct and indirect meta-analyses. *Lancet* 2006; 368(9546): 1503-15.

26. Cooper NJ, Sutton AJ, Lu G, Khunti K. Mixed comparison of stroke prevention treatments in individuals with nonrheumatic atrial fibrillation. *Archives of internal medicine* 2006; 166(12): 1269-75.

27. Davies L, Brown TJ, Haynes S, Payne K, Elliott RA, McCollum C. Cost-effectiveness of cell salvage and alternative methods of minimising perioperative allogeneic blood transfusion: a systematic review and economic model. *Health technology assessment (Winchester, England)* 2006; 10(44): iii-iv, ix-x, 1-210.

28. Eckert L, Falissard B. Using meta-regression in performing indirect-comparisons: comparing escitalopram with venlafaxine XR. *Current Medical Research and Opinion* 2006; 22(11): 2313-21.

29. Eckert L, Lancon C. Duloxetine compared with fluoxetine and venlafaxine: use of meta-regression analysis for indirect comparisons. *Bmc Psychiatry* 2006; 6: 30.

30. Gartlehner G, Hansen RA, Jonas BL, Thieda P, Lohr KN. The comparative efficacy and safety of biologics for the treatment of rheumatoid arthritis: a systematic review and metaanalysis. *The Journal of rheumatology* 2006; 33(12): 2398-408.

31. Jansen JP. Self-monitoring of glucose in type 2 diabetes mellitus: a Bayesian meta-analysis of direct and indirect comparisons. *Curr Med Res Opin* 2006; 22(4): 671-81.

32. Kearney PM, Baigent C, Godwin J, Halls H, Emberson JR, Patrono C. Do selective cyclo-oxygenase-2 inhibitors and traditional non-steroidal anti-inflammatory drugs increase the risk of atherothrombosis: meta-analysis of randomised trials (Structured abstract). *BMJ (Clinical research ed)*, 2006. http://onlinelibrary.wiley.com/o/cochrane/cldare/articles/DARE-12006008225/frame.html (accessed.

33. King S, Griffin S, Hodges Z, et al. A systematic review and economic model of the effectiveness and cost-effectiveness of methylphenidate, dexamfetamine and atomoxetine for the treatment of attention deficit hyperactivity disorder in children and adolescents. *Health technology assessment (Winchester, England)* 2006; 10(23): iii-iv, xiii-146.

34. Kyrgiou M, Salanti G, Pavlidis N, Paraskevaidis E, Ioannidis JP. Survival benefits with diverse chemotherapy regimens for ovarian cancer: meta-analysis of multiple treatments. *J Natl Cancer Inst* 2006; 98(22): 1655-63.

35. Pandor A, Eggington S, Paisley S, Tappenden P, Sutcliffe P. The clinical and cost-effectiveness of oxaliplatin and capecitabine for the adjuvant treatment of colon cancer: systematic review and economic evaluation. *Health technology assessment (Winchester, England)* 2006; 10(41): iii-iv, xi-xiv, 1-185.

36. Playford EG, Webster AC, Sorrell TC, Craig JC. Systematic review and meta-analysis of antifungal agents for preventing fungal infections in liver transplant recipients. *European journal of clinical microbiology & infectious diseases : official publication of the European Society of Clinical Microbiology* 2006; 25(9): 549-61.

37. Quan A, Chavanu K, Merkel J. A review of the efficacy of fixed-dose combinations olmesartan medoxomil/hydrochlorothiazide and amlodipine besylate/benazepril in factorial design studies. *American journal of cardiovascular drugs : drugs, devices, and other interventions* 2006; 6(2): 103-13.

38. Small LN, Lau J, Snydman DR. Preventing post-organ transplantation cytomegalovirus disease with ganciclovir: a meta-analysis comparing prophylactic and preemptive therapies. *Clinical infectious diseases : an official publication of the Infectious Diseases Society of America* 2006; 43(7): 869-80.

39. Stettler C, Allemann S, Egger M, Windecker S, Meier B, Diem P. Efficacy of drug eluting stents in patients with and without diabetes mellitus: indirect comparison of controlled trials. *Heart* 2006; 92(5): 650-7.

40. Woolacott N, Hawkins N, Mason A, et al. Etanercept and efalizumab for the treatment of psoriasis: a systematic review. *Health technology assessment (Winchester, England)* 2006; 10(46): 1-233, i-iv.

41. Wu P, Wilson K, Dimoulas P, Mills EJ. Effectiveness of smoking cessation therapies: a systematic review and meta-analysis. *BMC public health* 2006; 6: 300.

42. Zhou Z, Rahme E, Pilote L. Are statins created equal: evidence from randomized trials of pravastatin, simvastatin, and atorvastatin for cardiovascular disease prevention (Structured abstract). *American Heart Journal*, 2006. http://onlinelibrary.wiley.com/o/cochrane/cldare/articles/DARE-12006000856/frame.html (accessed.

43. Abou-Setta AM. What is the best site for embryo deposition? A systematic review and meta-analysis using direct and adjusted indirect comparisons. *Reproductive biomedicine online* 2007; 14(5): 611-9.

44. Berlie HD, Kalus JS, Jaber LA. Thiazolidinediones and the risk of edema: a meta-analysis (Structured abstract). *Diabetes Research and Clinical Practice*, 2007. http://onlinelibrary.wiley.com/o/cochrane/cldare/articles/DARE-12007001162/frame.html (accessed.

45. Bolen S, Feldman L, Vassy J, et al. Systematic review: Comparative effectiveness and safety of oral medications for type 2 diabetes Mellitus. *Annals of Internal Medicine* 2007; 147(6): 386-99.

46. Boonen S, Lips P, Bouillon R, Bischoff-Ferrari HA, Vanderschueren D, Haentjens P. Need for additional calcium to reduce the risk of hip fracture with vitamin d supplementation: evidence from a comparative metaanalysis of randomized controlled trials. *The Journal of clinical endocrinology and metabolism* 2007; 92(4): 1415-23.

47. Buscemi N, Vandermeer B, Friesen C, et al. The efficacy and safety of drug treatments for chronic insomnia in adults: a meta-analysis of RCTs. *J Gen Intern Med* 2007; 22(9): 1335-50.

48. Collins R, Fenwick E, Trowman R, et al. A systematic review and economic model of the clinical effectiveness and cost-effectiveness of docetaxel in combination with prednisone or prednisolone for the treatment of hormone-refractory metastatic prostate cancer (Structured abstract). *Health Technology Assessment*, 2007. http://onlinelibrary.wiley.com/o/cochrane/cldare/articles/DARE-12007008058/frame.html (accessed.

49. Connock M, Juarez-Garcia A, Jowett S, et al. Methadone and buprenorphine for the management of opioid dependence: a systematic review and economic evaluation. *Health technology assessment (Winchester, England)* 2007; 11(9): 1-171, iii-iv.

50. Elliott WJ, Meyer PM. Incident diabetes in clinical trials of antihypertensive drugs: a network meta-analysis. *Lancet* 2007; 369(9557): 201-7.

51. Golfinopoulos V, Salanti G, Pavlidis N, Ioannidis JP. Survival and disease-progression benefits with treatment regimens for advanced colorectal cancer: a meta-analysis. *The lancet oncology* 2007; 8(10): 898-911.

52. Lam SK, Owen A. Combined resynchronisation and implantable defibrillator therapy in left ventricular dysfunction: Bayesian network meta-analysis of randomised controlled trials. *BMJ (Clinical research ed)* 2007; 335(7626): 925.

53. Lee YH, Ji JD, Song GG. Adjusted indirect comparison of celecoxib versus rofecoxib on cardiovascular risk. *Rheumatology International* 2007; 27(5): 477-82.

54. McLeod C, Bagust A, Boland A, et al. Adalimumab, etanercept and infliximab for the treatment of ankylosing spondylitis: a systematic review and economic evaluation. *Health technology assessment (Winchester, England)* 2007; 11(28): 1-158, iii-iv.

55. Nixon R, Bansback N, Brennan A. The efficacy of inhibiting tumour necrosis factor alpha and interleukin 1 in patients with rheumatoid arthritis: a meta-analysis and adjusted indirect comparisons. *Rheumatology (Oxford, England)* 2007; 46(7): 1140-7.

56. Stettler C, Wandel S, Allemann S, et al. Outcomes associated with drug-eluting and bare-metal stents: a collaborative network meta-analysis. *Lancet* 2007; 370(9591): 937-48.

57. Tudur Smith C, Marson AG, Chadwick DW, Williamson PR. Multiple treatment comparisons in epilepsy monotherapy trials. *Trials* 2007; 8: 34.

58. Vandermeer BW, Buscemi N, Liang Y, Witmans M. Comparison of meta-analytic results of indirect, direct, and combined comparisons of drugs for chronic insomnia in adults: a case study. *Medical care* 2007; 45(10 Supl 2): S166-72.

59. Vestergaard P, Jorgensen NR, Mosekilde L, Schwarz P. Effects of parathyroid hormone alone or in combination with antiresorptive therapy on bone mineral density and fracture risk--a meta-analysis. *Osteoporosis international : a journal established as result of cooperation between the European Foundation for Osteoporosis and the National Osteoporosis Foundation of the USA* 2007; 18(1): 45-57.

60. Ward S, Simpson E, Davis S, Hind D, Rees A, Wilkinson A. Taxanes for the adjuvant treatment early breast cancer: systematic review and economic evaluation (Structured abstract). *Health Technology Assessment Database*, 2007. http://onlinelibrary.wiley.com/o/cochrane/clhta/articles/HTA-32007000627/frame.html (accessed.

61. Abdullah AK, Khan S. Relative oral corticosteroid-sparing effect of 7 inhaled corticosteroids in chronic asthma: a meta-analysis. *Annals of allergy, asthma & immunology : official publication of the American College of Allergy, Asthma, & Immunology* 2008; 101(1): 74-81.

62. Bekkering GE, Abou-Setta AM, Kleijnen J. The application of quantitative methods for identifying and exploring the presence of bias in systematic reviews: PDE-5 inhibitors for erectile dysfunction (Structured abstract). *International Journal of Impotence Research*, 2008. http://onlinelibrary.wiley.com/o/cochrane/cldare/articles/DARE-12008104519/frame.html (accessed.

63. Chou R, Carson S, Chan BK. Pegylated interferons for chronic hepatitis C virus infection: an indirect analysis of randomized trials. *Journal of viral hepatitis* 2008; 15(8): 551-70.

64. Coleman CI, Baker WL, Kluger J, White CM. Antihypertensive medication and their impact on cancer incidence: a mixed treatment comparison meta-analysis of randomized controlled trials. *Journal of hypertension* 2008; 26(4): 622-9.

65. Eisenberg MJ, Filion KB, Yavin D, et al. Pharmacotherapies for smoking cessation: a meta-analysis of randomized controlled trials. *CMAJ : Canadian Medical Association journal = journal de l'Association medicale canadienne* 2008; 179(2): 135-44.

66. Engelman E, Salengros JC, Barvais L. How much does pharmacologic prophylaxis reduce postoperative vomiting in children? Calculation of prophylaxis effectiveness and expected incidence of vomiting under treatment using Bayesian meta-analysis (Structured abstract). *Anesthesiology*, 2008. http://onlinelibrary.wiley.com/o/cochrane/cldare/articles/DARE-12009102401/frame.html (accessed.

67. Fakhoury W, Lockhart I, Kotchie RW, Aagren M, LeReun C. Indirect comparison of once daily insulin detemir and glargine in reducing weight gain and hypoglycaemic episodes when administered in addition to conventional oral anti-diabetic therapy in patients with type-2 diabetes. *Pharmacology* 2008; 82(2): 156-63.

68. Hansen RA, Gartlehner G, Webb AP, Morgan LC, Moore CG, Jonas DE. Efficacy and safety of donepezil, galantamine, and rivastigmine for the treatment of Alzheimer's disease: a systematic review and meta-analysis. *Clinical interventions in aging* 2008; 3(2): 211-25.

69. Hansen RA, Gaynes BN, Gartlehner G, Moore CG, Tiwari R, Lohr KN. Efficacy and tolerability of second-generation antidepressants in social anxiety disorder. *International clinical psychopharmacology* 2008; 23(3): 170-9.

70. Lee YH, Woo JH, Rho YH, Choi SJ, Ji JD, Song GG. Meta-analysis of the combination of TNF inhibitors plus MTX compared to MTX monotherapy, and the adjusted indirect comparison of TNF inhibitors in patients suffering from active rheumatoid arthritis. *Rheumatol Int* 2008; 28(6): 553-9.

71. Mauri D, Polyzos NP, Salanti G, Pavlidis N, Ioannidis JP. Multiple-treatments meta-analysis of chemotherapy and targeted therapies in advanced breast cancer. *J Natl Cancer Inst* 2008; 100(24): 1780-91.

72. Mills EJ, Rachlis B, Wu P, Devereaux PJ, Arora P, Perri D. Primary prevention of cardiovascular mortality and events with statin treatments: a network meta-analysis involving more than 65,000 patients. *J Am Coll Cardiol* 2008; 52(22): 1769-81.

73. Peterson K, McDonagh MS, Fu R. Comparative benefits and harms of competing medications for adults with attention-deficit hyperactivity disorder: a systematic review and indirect comparison meta-analysis. *Psychopharmacology (Berl)* 2008; 197(1): 1-11.

74. Quilici S, Abrams KR, Nicolas A, et al. Meta-analysis of the efficacy and tolerability of pramipexole versus ropinirole in the treatment of restless legs syndrome. *Sleep medicine* 2008; 9(7): 715-26.

75. Saad AA, Symmons DP, Noyce PR, Ashcroft DM. Risks and benefits of tumor necrosis factor-alpha inhibitors in the management of psoriatic arthritis: systematic review and metaanalysis of randomized controlled trials (Structured abstract). *Journal of Rheumatology*, 2008. http://onlinelibrary.wiley.com/o/cochrane/cldare/articles/DARE-12008105130/frame.html (accessed.

76. Snick HK, Collins JA, Evers JL. What is the most valid comparison treatment in trials of intrauterine insemination, timed or uninfluenced intercourse? A systematic review and meta-analysis of indirect evidence (Structured abstract). *Human Reproduction*, 2008. http://onlinelibrary.wiley.com/o/cochrane/cldare/articles/DARE-12009100949/frame.html (accessed.

77. Stettler C, Allemann S, Wandel S, et al. Drug eluting and bare metal stents in people with and without diabetes: collaborative network meta-analysis. *BMJ (Clinical research ed)* 2008; 337: a1331.

78. Sultana A, Ghaneh P, Cunningham D, Starling N, Neoptolemos JP, Smith CT. Gemcitabine based combination chemotherapy in advanced pancreatic cancer-indirect comparison. *Bmc Cancer* 2008; 8: 192.

79. Swart AM, Burdett S, Ledermann J, Mook P, Parmar MK. Why i.p. therapy cannot yet be considered as a standard of care for the first-line treatment of ovarian cancer: a systematic review. *Annals of oncology : official journal of the European Society for Medical Oncology / ESMO* 2008; 19(4): 688-95.

80. Testa L, Gaal WJ, Biondi-Zoccai GG, et al. Repeat thrombolysis or conservative therapy vs. rescue percutaneous coronary intervention for failed thrombolysis: systematic review and meta-analysis (Structured abstract). *QJM: an International Journal of Medicine*, 2008. http://onlinelibrary.wiley.com/o/cochrane/cldare/articles/DARE-12008106791/frame.html (accessed.

81. Thijs V, Lemmens R, Fieuws S. Network meta-analysis: simultaneous meta-analysis of common antiplatelet regimens after transient ischaemic attack or stroke. *Eur Heart J* 2008; 29(9): 1086-92.

82. Tonelli M, Klarenbach S, Wiebe N, Shrive F, Hemmelgarn B, Manns B. Erythropoiesis-stimulating agents for anemia of chronic kidney disease: systematic review and economic evaluation (Structured abstract). *Database of Abstracts of Reviews of Effects*, 2008. http://onlinelibrary.wiley.com/o/cochrane/cldare/articles/DARE-12009109183/frame.html (accessed.

83. Baker WL, Baker EL, Coleman CI. Pharmacologic treatments for chronic obstructive pulmonary disease: a mixed-treatment comparison meta-analysis. *Pharmacotherapy* 2009; 29(8): 891-905.

84. Bansback N, Sizto S, Sun H, Feldman S, Willian MK, Anis A. Efficacy of systemic treatments for moderate to severe plaque psoriasis: systematic review and meta-analysis. *Dermatology* 2009; 219(3): 209-18.

85. Chou R, Carson S, Chan BK. Gabapentin versus tricyclic antidepressants for diabetic neuropathy and post-herpetic neuralgia: discrepancies between direct and indirect meta-analyses of randomized controlled trials. *J Gen Intern Med* 2009; 24(2): 178-88.

86. Cipriani A, Furukawa TA, Salanti G, et al. Comparative efficacy and acceptability of 12 new-generation antidepressants: a multiple-treatments meta-analysis. *Lancet* 2009; 373(9665): 746-58.

87. Edwards SJ, Clarke MJ, Wordsworth S, Welton NJ. Carbapenems versus other beta-lactams in the treatment of hospitalised patients with infection: a mixed treatment comparison. *Curr Med Res Opin* 2009; 25(1): 251-61.

88. Edwards SJ, Lind T, Lundell L, Das R. Systematic review: standard- and double-dose proton pump inhibitors for the healing of severe erosive oesophagitis -- a mixed treatment comparison of randomized controlled trials. *Aliment Pharmacol Ther* 2009; 30(6): 547-56.

89. Edwards SJ, Smith CJ. Tolerability of atypical antipsychotics in the treatment of adults with schizophrenia or bipolar disorder: a mixed treatment comparison of randomized controlled trials. *Clin Ther* 2009; 31 Pt 1: 1345-59.

90. Golfinopoulos V, Pentheroudakis G, Salanti G, Nearchou AD, Ioannidis JP, Pavlidis N. Comparative survival with diverse chemotherapy regimens for cancer of unknown primary site: multiple-treatments meta-analysis. *Cancer Treat Rev* 2009; 35(7): 570-3.

91. Hofmeyr GJ, Gulmezoglu AM, Novikova N, Linder V, Ferreira S, Piaggio G. Misoprostol to prevent and treat postpartum haemorrhage: a systematic review and meta-analysis of maternal deaths and dose-related effects. *Bulletin of the World Health Organization* 2009; 87(9): 666-77.

92. Jansen JP, Bergman GJ, Huels J, Olson M. Prevention of vertebral fractures in osteoporosis: mixed treatment comparison of bisphosphonate therapies. *Curr Med Res Opin* 2009; 25(8): 1861-8.

93. Kotze A, Scally A, Howell S. Efficacy and safety of different techniques of paravertebral block for analgesia after thoracotomy: a systematic review and metaregression. *Br J Anaesth* 2009; 103(5): 626-36.

94. Lim E, Harris G, Patel A, Adachi I, Edmonds L, Song F. Preoperative versus postoperative chemotherapy in patients with resectable non-small cell lung cancer: systematic review and indirect comparison meta-analysis of randomized trials. *Journal of thoracic oncology : official publication of the International Association for the Study of Lung Cancer* 2009; 4(11): 1380-8.

95. Maas T, Kaper J, Sheikh A, et al. Mono and multifaceted inhalant and/or food allergen reduction interventions for preventing asthma in children at high risk of developing asthma. *The Cochrane database of systematic reviews* 2009; (3): CD006480.

96. Manzoli L, Salanti G, De Vito C, Boccia A, Ioannidis JP, Villari P. Immunogenicity and adverse events of avian influenza A H5N1 vaccine in healthy adults: multiple-treatments meta-analysis. *The Lancet infectious diseases* 2009; 9(8): 482-92.

97. Mills EJ, Perri D, Cooper C, et al. Antifungal treatment for invasive Candida infections: a mixed treatment comparison meta-analysis. *Annals of clinical microbiology and antimicrobials* 2009; 8: 23.

98. Mills EJ, Rachlis B, O'Regan C, Thabane L, Perri D. Metastatic renal cell cancer treatments: an indirect comparison meta-analysis. *Bmc Cancer* 2009; 9: 34.

99. Mills EJ, Wu P, Spurden D, Ebbert JO, Wilson K. Efficacy of pharmacotherapies for short-term smoking abstinance: a systematic review and meta-analysis. *Harm reduction journal* 2009; 6: 25.

100. Peuter OR, Lussana F, Peters RJ, Buller HR, Kamphuisen PW. A systematic review of selective and non-selective beta blockers for prevention of vascular events in patients with acute coronary syndrome or heart failure (Structured abstract). *Netherlands Journal of Medicine*, 2009. http://onlinelibrary.wiley.com/o/cochrane/cldare/articles/DARE-12010000018/frame.html (accessed.

101. Piccini JP, Hasselblad V, Peterson ED, Washam JB, Califf RM, Kong DF. Comparative efficacy of dronedarone and amiodarone for the maintenance of sinus rhythm in patients with atrial fibrillation. *J Am Coll Cardiol* 2009; 54(12): 1089-95.

102. Pignon JP, le Maitre A, Maillard E, Bourhis J. Meta-analysis of chemotherapy in head and neck cancer (MACH-NC): an update on 93 randomised trials and 17,346 patients. *Radiotherapy and oncology : journal of the European Society for Therapeutic Radiology and Oncology* 2009; 92(1): 4-14.

103. Puhan MA, Bachmann LM, Kleijnen J, Ter Riet G, Kessels AG. Inhaled drugs to reduce exacerbations in patients with chronic obstructive pulmonary disease: a network meta-analysis. *BMC Med* 2009; 7: 2.

104. Quilici S, Chancellor J, Lothgren M, et al. Meta-analysis of duloxetine vs. pregabalin and gabapentin in the treatment of diabetic peripheral neuropathic pain. *BMC neurology* 2009; 9: 6.

105. Richy FF, Banerjee S, Brabant Y, Helmers S. Levetiracetam extended release and levetiracetam immediate release as adjunctive treatment for partial-onset seizures: an indirect comparison of treatment-emergent adverse events using meta-analytic techniques. *Epilepsy & behavior : E&B* 2009; 16(2): 240-5.

106. Singh JA, Christensen R, Wells GA, et al. Biologics for rheumatoid arthritis: an overview of Cochrane reviews. *The Cochrane database of systematic reviews* 2009; (4): CD007848.

107. Strassmann R, Bausch B, Spaar A, Keijnen J, Braendli O, Puhan MA. Smoking cessation interventions in COPD: a network meta-analysis of randomised trials. *European Respiratory Journal* 2009; 34(3): 634-40.

108. Thompson Coon JS, Liu Z, Hoyle M, et al. Sunitinib and bevacizumab for first-line treatment of metastatic renal cell carcinoma: a systematic review and indirect comparison of clinical effectiveness (Structured abstract). *British Journal of Cancer*, 2009. http://onlinelibrary.wiley.com/o/cochrane/cldare/articles/DARE-12009106974/frame.html (accessed.

109. Trikalinos TA, Alsheikh-Ali AA, Tatsioni A, Nallamothu BK, Kent DM. Percutaneous coronary interventions for non-acute coronary artery disease: a quantitative 20-year synopsis and a network meta-analysis. *Lancet* 2009; 373(9667): 911-8.

110. van der Valk R, Webers CA, Lumley T, Hendrikse F, Prins MH, Schouten JS. A network meta-analysis combined direct and indirect comparisons between glaucoma drugs to rank effectiveness in lowering intraocular pressure. *J Clin Epidemiol* 2009; 62(12): 1279-83.

111. Welton NJ, Caldwell DM, Adamopoulos E, Vedhara K. Mixed treatment comparison meta-analysis of complex interventions: psychological interventions in coronary heart disease. *American journal of epidemiology* 2009; 169(9): 1158-65.

112. Benedict A, Verdian L, Maclaine G. The cost effectiveness of rufinamide in the treatment of Lennox-Gastaut syndrome in the UK. *Pharmacoeconomics* 2010; 28(3): 185-99.

113. Bergman GJ, Hochberg MC, Boers M, Wintfeld N, Kielhorn A, Jansen JP. Indirect comparison of tocilizumab and other biologic agents in patients with rheumatoid arthritis and inadequate response to disease-modifying antirheumatic drugs. *Seminars in arthritis and rheumatism* 2010; 39(6): 425-41.

114. Bolanos R, Francia J. Isoflavones versus hormone therapy for reduction of vertebral fracture risk: indirect comparison. *Menopause (New York, NY)* 2010; 17(6): 1201-5.

115. Dakin H, Fidler C, Harper C. Mixed treatment comparison meta-analysis evaluating the relative efficacy of nucleos(t)ides for treatment of nucleos(t)ide-naive patients with chronic hepatitis B. *Value in health : the journal of the International Society for Pharmacoeconomics and Outcomes Research* 2010; 13(8): 934-45.

116. Delahoy P, Thompson S, Marschner IC. Pregabalin versus gabapentin in partial epilepsy: a meta-analysis of dose-response relationships. *BMC neurology* 2010; 10: 104.

117. Germani G, Pleguezuelo M, Gurusamy K, Meyer T, Isgro G, Burroughs AK. Clinical outcomes of radiofrequency ablation, percutaneous alcohol and acetic acid injection for hepatocelullar carcinoma: a meta-analysis. *Journal of hepatology* 2010; 52(3): 380-8.

118. Hauser W, Petzke F, Sommer C. Comparative efficacy and harms of duloxetine, milnacipran, and pregabalin in fibromyalgia syndrome. *The journal of pain : official journal of the American Pain Society* 2010; 11(6): 505-21.

119. Imamura M, Abrams P, Bain C, et al. Systematic review and economic modelling of the effectiveness and cost-effectiveness of non-surgical treatments for women with stress urinary incontinence. *Health technology assessment (Winchester, England)* 2010; 14(40): 1-188, iii-iv.

120. Jansen JP, Gaugris S, Choy EH, Ostor A, Nash JT, Stam W. Cost effectiveness of etoricoxib versus celecoxib and non-selective NSAIDS in the treatment of ankylosing spondylitis. *Pharmacoeconomics* 2010; 28(4): 323-44.

121. Knight C, Howard P, Baker CL, Marton JP. The cost-effectiveness of an extended course (12+12 weeks) of varenicline compared with other available smoking cessation strategies in the United States: an extension and update to the BENESCO model. *Value in health : the journal of the International Society for Pharmacoeconomics and Outcomes Research* 2010; 13(2): 209-14.

122. Latthe PM, Singh P, Foon R, Toozs-Hobson P. Two routes of transobturator tape procedures in stress urinary incontinence: a meta-analysis with direct and indirect comparison of randomized trials. *BJU international* 2010; 106(1): 68-76.

123. Logman JFS, Stephens J, Heeg B, et al. Comparative effectiveness of antibiotics for the treatment of MRSA complicated skin and soft tissue infections (Structured abstract). *Current Medical Research and Opinion*, 2010. http://onlinelibrary.wiley.com/o/cochrane/cldare/articles/DARE-12010004682/frame.html (accessed.

124. McKenna C, Burch J, Suekarran S, et al. A systematic review and economic evaluation of the clinical effectiveness and cost-effectiveness of aldosterone antagonists for postmyocardial infarction heart failure. *Health Technology Assessment* 2010; 14(24): 1-+.

125. Meader N. A comparison of methadone, buprenorphine and alpha(2) adrenergic agonists for opioid detoxification: a mixed treatment comparison meta-analysis. *Drug and alcohol dependence* 2010; 108(1-2): 110-4.

126. Middleton LJ, Champaneria R, Daniels JP, et al. Hysterectomy, endometrial destruction, and levonorgestrel releasing intrauterine system (Mirena) for heavy menstrual bleeding: systematic review and meta-analysis of data from individual patients. *BMJ (Clinical research ed)* 2010; 341: c3929.

127. Nuijten M, Heigener DF, Bischoff HG, et al. Effectiveness of bevacizumab- and pemetrexed-cisplatin treatment for patients with advanced non-squamous non-small cell lung cancer. *Lung Cancer* 2010; 69 Suppl 1: S4-10.

128. Orme M, Collins S, Dakin H, Kelly S, Loftus J. Mixed treatment comparison and meta-regression of the efficacy and safety of prostaglandin analogues and comparators for primary open-angle glaucoma and ocular hypertension. *Curr Med Res Opin* 2010; 26(3): 511-28.

129. Owen A. Antithrombotic treatment for the primary prevention of stroke in patients with non valvular atrial fibrillation: a reappraisal of the evidence and network meta analysis. *Int J Cardiol* 2010; 142(3): 218-23.

130. Phung OJ, Scholle JM, Talwar M, Coleman CI. Effect of noninsulin antidiabetic drugs added to metformin therapy on glycemic control, weight gain, and hypoglycemia in type 2 diabetes. *Jama* 2010; 303(14): 1410-8.

131. Riemsma R, Forbes CA, Kessels A, et al. Systematic review of aromatase inhibitors in the first-line treatment for hormone sensitive advanced or metastatic breast cancer. *Breast Cancer Res Treat* 2010; 123(1): 9-24.

132. Riemsma R, Simons JP, Bashir Z, Gooch CL, Kleijnen J. Systematic Review of topotecan (Hycamtin) in relapsed small cell lung cancer. *Bmc Cancer* 2010; 10: 436.

133. Roskell NS, Lip GY, Noack H, Clemens A, Plumb JM. Treatments for stroke prevention in atrial fibrillation: a network meta-analysis and indirect comparisons versus dabigatran etexilate. *Thromb Haemost* 2010; 104(6): 1106-15.

134. Stowe R, Ives N, Clarke CE, et al. Evaluation of the efficacy and safety of adjuvant treatment to levodopa therapy in Parkinson s disease patients with motor complications. *The Cochrane database of systematic reviews* 2010; (7): CD007166.

135. Trkulja V, Kolundzic R. Rivaroxaban vs dabigatran for thromboprophylaxis after joint-replacement surgery: exploratory indirect comparison based on meta-analysis of pivotal clinical trials. *Croatian medical journal* 2010; 51(2): 113-23.

136. Tu YK, Woolston A, Faggion CM, Jr. Do bone grafts or barrier membranes provide additional treatment effects for infrabony lesions treated with enamel matrix derivatives? A network meta-analysis of randomized-controlled trials. *J Clin Periodontol* 2010; 37(1): 59-79.

137. Tzellos TG, Toulis KA, Goulis DG, et al. Gabapentin and pregabalin in the treatment of fibromyalgia: a systematic review and a meta-analysis. *J Clin Pharm Ther* 2010; 35(6): 639-56.

138. Uthman OA, Abdulmalik J. Comparative efficacy and acceptability of pharmacotherapeutic agents for anxiety disorders in children and adolescents: a mixed treatment comparison meta-analysis. *Curr Med Res Opin* 2010; 26(1): 53-9.

139. Verdian L, Yi Y. Cost-utility analysis of rufinamide versus topiramate and lamotrigine for the treatment of children with Lennox-Gastaut Syndrome in the United Kingdom. *Seizure : the journal of the British Epilepsy Association* 2010; 19(1): 1-11.

140. Vissers D, Stam W, Nolte T, Lenre M, Jansen J. Efficacy of intranasal fentanyl spray versus other opioids for breakthrough pain in cancer. *Curr Med Res Opin* 2010; 26(5): 1037-45.

141. Walsh T, Worthington HV, Glenny AM, Appelbe P, Marinho VC, Shi X. Fluoride toothpastes of different concentrations for preventing dental caries in children and adolescents. *The Cochrane database of systematic reviews* 2010; (1): CD007868.

142. Wandel S, Juni P, Tendal B, et al. Effects of glucosamine, chondroitin, or placebo in patients with osteoarthritis of hip or knee: network meta-analysis. *BMJ (Clinical research ed)* 2010; 341: c4675.

143. Wang H, Huang T, Jing J, et al. Effectiveness of different central venous catheters for catheter-related infections: a network meta-analysis. *The Journal of hospital infection* 2010; 76(1): 1-11.

144. Wolff RF, Bala MM, Westwood M, Kessels AG, Kleijnen J. 5% lidocaine medicated plaster in painful diabetic peripheral neuropathy (DPN): a systematic review. *Swiss medical weekly* 2010; 140(21-22): 297-306.

145. Woo G, Tomlinson G, Nishikawa Y, et al. Tenofovir and entecavir are the most effective antiviral agents for chronic hepatitis B: a systematic review and Bayesian meta-analyses. *Gastroenterology* 2010; 139(4): 1218-29.

146. Ahn M-J, Tsai C-M, Hsia T-C, et al. Cost-effectiveness of bevacizumab-based therapy versus cisplatin plus pemetrexed for the first-line treatment of advanced non-squamous NSCLC in Korea and Taiwan. *Asia-Pacific Journal of Clinical Oncology* 2011; 7: 22-33.

147. Anothaisintawee T, Attia J, Nickel JC, et al. Management of chronic prostatitis/chronic pelvic pain syndrome: a systematic review and network meta-analysis. *Jama* 2011; 305(1): 78-86.

148. Baldwin D, Woods R, Lawson R, Taylor D. Efficacy of drug treatments for generalised anxiety disorder: systematic review and meta-analysis. *BMJ (Clinical research ed)* 2011; 342: d1199.

149. Bangalore S, Kumar S, Kjeldsen SE, et al. Antihypertensive drugs and risk of cancer: network meta-analyses and trial sequential analyses of 324,168 participants from randomised trials. *The lancet oncology* 2011; 12(1): 65-82.

150. Beard SM, Roskell N, Le TK, et al. Cost effectiveness of duloxetine in the treatment of fibromyalgia in the United States. *Journal of medical economics* 2011; 14(4): 463-76.

151. Bekkering GE, Soares-Weiser K, Reid K, et al. Can morphine still be considered to be the standard for treating chronic pain? A systematic review including pair-wise and network meta-analyses. *Curr Med Res Opin* 2011; 27(7): 1477-91.

152. Biondi-Zoccai G, Lotrionte M, Agostoni P, et al. Adjusted indirect comparison meta-analysis of prasugrel versus ticagrelor for patients with acute coronary syndromes. *Int J Cardiol* 2011; 150(3): 325-31.

153. Blanchard P, Hill C, Guihenneuc-Jouyaux C, Baey C, Bourhis J, Pignon JP. Mixed treatment comparison meta-analysis of altered fractionated radiotherapy and chemotherapy in head and neck cancer. *J Clin Epidemiol* 2011; 64(9): 985-92.

154. Bolanos-Diaz R, Zavala-Gonzales JC, Mezones-Holguin E, Francia-Romero J. Soy extracts versus hormone therapy for reduction of menopausal hot flushes: indirect comparison. *Menopause (New York, NY)* 2011; 18(7): 825-9.

155. Bottomley JM, Taylor RS, Ryttov J. The effectiveness of two-compound formulation calcipotriol and betamethasone dipropionate gel in the treatment of moderately severe scalp psoriasis: a systematic review of direct and indirect evidence. *Curr Med Res Opin* 2011; 27(1): 251-68.

156. Cassese S, Piccolo R, Galasso G, De Rosa R, Pierri A, Piscione F. Manual versus mechanical thrombectomy in patients with acute myocardial infarction undergoing percutaneous coronary intervention: An adjusted indirect comparison of randomised trials. *European Heart Journal* 2011; 32: 412.

157. Chang JW, Thongprasert S, Wright E, et al. An indirect comparison of bevacizumab plus cisplatin-gemcitabine and cisplatin plus pemetrexed treatment for patients with advanced first-line non-squamous non-small cell lung cancer in East Asia. *Asia-Pacific journal of clinical oncology* 2011; 7 Suppl 2: 13-21.

158. Chou D, Lau D, Hermsmeyer J, Norvell D. Efficacy of interspinous device versus surgical decompression in the treatment of lumbar spinal stenosis: a modified network analysis (Structured abstract). *Database of Abstracts of Reviews of Effects*, 2011. http://onlinelibrary.wiley.com/o/cochrane/cldare/articles/DARE-12013007035/frame.html (accessed.

159. Choy E, Marshall D, Gabriel ZL, Mitchell SA, Gylee E, Dakin HA. A systematic review and mixed treatment comparison of the efficacy of pharmacological treatments for fibromyalgia. *Seminars in arthritis and rheumatism* 2011; 41(3): 335-45 e6.

160. Cipriani A, Barbui C, Salanti G, et al. Comparative efficacy and acceptability of antimanic drugs in acute mania: a multiple-treatments meta-analysis. *Lancet* 2011; 378(9799): 1306-15.

161. Cope S, Capkun-Niggli G, Gale R, Jardim JR, Jansen JP. Comparative efficacy of indacaterol 150 mug and 300 mug versus fixed-dose combinations of formoterol + budesonide or salmeterol + fluticasone for the treatment of chronic obstructive pulmonary disease--a network meta-analysis. *Int J Chron Obstruct Pulmon Dis* 2011; 6: 329-44.

162. Costa J, Fareleira F, Ascencao R, Borges M, Sampaio C, Vaz-Carneiro A. Clinical comparability of the new antiepileptic drugs in refractory partial epilepsy: a systematic review and meta-analysis. *Epilepsia* 2011; 52(7): 1280-91.

163. Cummins E, Asseburg C, Punekar YS, et al. Cost-effectiveness of infliximab for the treatment of active and progressive psoriatic arthritis. *Value in health : the journal of the International Society for Pharmacoeconomics and Outcomes Research* 2011; 14(1): 15-23.

164. Danchin N, Marzilli M, Parkhomenko A, Ribeiro JP. Efficacy comparison of trimetazidine with therapeutic alternatives in stable angina pectoris: a network meta-analysis. *Cardiology* 2011; 120(2): 59-72.

165. Devine EB, Alfonso-Cristancho R, Sullivan SD. Effectiveness of biologic therapies for rheumatoid arthritis: an indirect comparisons approach. *Pharmacotherapy* 2011; 31(1): 39-51.

166. Dranitsaris G, Jelincic V, Choe Y. Meta regression analysis to indirectly compare dalteparin to enoxaparin for the prevention of venous thromboembolic events following total hip replacement. *Thrombosis Journal* 2011; 9.

167. Fleeman N, Bagust A, Boland A, et al. Lapatinib and trastuzumab in combination with an aromatase inhibitor for the first-line treatment of metastatic hormone receptor-positive breast cancer which over-expresses human epidermal growth factor 2 (HER2): a systematic review and economic analysis. *Health technology assessment (Winchester, England)* 2011; 15(42): 1-93, iii-iv.

168. Freemantle N, Lafuente-Lafuente C, Mitchell S, Eckert L, Reynolds M. Mixed treatment comparison of dronedarone, amiodarone, sotalol, flecainide, and propafenone, for the management of atrial fibrillation. *Europace : European pacing, arrhythmias, and cardiac electrophysiology : journal of the working groups on cardiac pacing, arrhythmias, and cardiac cellular electrophysiology of the European Society of Cardiology* 2011; 13(3): 329-45.

169. Freemantle N, Tharmanathan P, Herbrecht R. Systematic review and mixed treatment comparison of randomized evidence for empirical, pre-emptive and directed treatment strategies for invasive mould disease. *The Journal of antimicrobial chemotherapy* 2011; 66 Suppl 1: i25-35.

170. Gartlehner G, Hansen RA, Morgan LC, et al. Comparative benefits and harms of second-generation antidepressants for treating major depressive disorder: an updated meta-analysis. *Ann Intern Med* 2011; 155(11): 772-85.

171. Gartlehner G, Hansen RA, Morgan LC, et al. Second-Generation Antidepressants in the Pharmacologic Treatment of Adult Depression: An Update of the 2007 Comparative Effectiveness Review. Rockville MD; 2011.

172. Gines J, Sabater E, Martorell C, Grau M, Monroy M, Casado MA. Efficacy of taxanes as adjuvant treatment of breast cancer: a review and meta-analysis of randomised clinical trials. *Clinical & translational oncology : official publication of the Federation of Spanish Oncology Societies and of the National Cancer Institute of Mexico* 2011; 13(7): 485-98.

173. Greenhalgh J, Bagust A, Boland A, et al. Clopidogrel and modified-release dipyridamole for the prevention of occlusive vascular events (review of Technology Appraisal No. 90): a systematic review and economic analysis. *Health technology assessment (Winchester, England)* 2011; 15(31): 1-178.

174. Gross JL, Kramer CK, Leitao CB, et al. Effect of antihyperglycemic agents added to metformin and a sulfonylurea on glycemic control and weight gain in type 2 diabetes: a network meta-analysis. *Ann Intern Med* 2011; 154(10): 672-9.

175. Gurusamy KS, Pissanou T, Pikhart H, Vaughan J, Burroughs AK, Davidson BR. Methods to decrease blood loss and transfusion requirements for liver transplantation. *The Cochrane database of systematic reviews* 2011; (12): CD009052.

176. Guyot P, Taylor P, Christensen R, et al. Abatacept with methotrexate versus other biologic agents in treatment of patients with active rheumatoid arthritis despite methotrexate: a network meta-analysis. *Arthritis Res Ther* 2011; 13(6): R204.

177. Halpin DM, Gray J, Edwards SJ, Morais J, Singh D. Budesonide/formoterol vs. salmeterol/fluticasone in COPD: a systematic review and adjusted indirect comparison of pneumonia in randomised controlled trials. *Int J Clin Pract* 2011; 65(7): 764-74.

178. Hartling L, Fernandes RM, Bialy L, et al. Steroids and bronchodilators for acute bronchiolitis in the first two years of life: systematic review and meta-analysis. *BMJ (Clinical research ed)* 2011; 342: d1714.

179. Hauser W, Petzke F, Uceyler N, Sommer C. Comparative efficacy and acceptability of amitriptyline, duloxetine and milnacipran in fibromyalgia syndrome: a systematic review with meta-analysis. *Rheumatology (Oxford, England)* 2011; 50(3): 532-43.

180. Hopkins RB, Goeree R, Pullenayegum E, et al. The relative efficacy of nine osteoporosis medications for reducing the rate of fractures in post-menopausal women (Structured abstract). *BMC Musculoskeletal Disorders*, 2011. http://onlinelibrary.wiley.com/o/cochrane/cldare/articles/DARE-12011006933/frame.html (accessed.

181. Ibrahim T, Qureshi A, Sutton AJ, Dias JJ. Surgical versus nonsurgical treatment of acute minimally displaced and undisplaced scaphoid waist fractures: pairwise and network meta-analyses of randomized controlled trials. *The Journal of hand surgery* 2011; 36(11): 1759-68 e1.

182. Jalota L, Kalira V, George E, et al. Prevention of pain on injection of propofol: systematic review and meta-analysis. *BMJ (Clinical research ed)* 2011; 342: d1110.

183. Jansen JP, Bergman GJ, Huels J, Olson M. The efficacy of bisphosphonates in the prevention of vertebral, hip, and nonvertebral-nonhip fractures in osteoporosis: a network meta-analysis. *Seminars in arthritis and rheumatism* 2011; 40(4): 275-84 e1-2.

184. Jones LJ, Craven PD, Attia J, Thakkinstian A, Wright I. Network meta-analysis of indomethacin versus ibuprofen versus placebo for PDA in preterm infants. *Archives of disease in childhood Fetal and neonatal edition* 2011; 96(1): F45-52.

185. Kalil AC, Mindru C, Florescu DF. Effectiveness of valganciclovir 900 mg versus 450 mg for cytomegalovirus prophylaxis in transplantation: direct and indirect treatment comparison meta-analysis. *Clinical infectious diseases : an official publication of the Infectious Diseases Society of America* 2011; 52(3): 313-21.

186. Klemp M, Tvete IF, Skomedal T, Gaasemyr J, Natvig B, Aursnes I. A review and Bayesian meta-analysis of clinical efficacy and adverse effects of 4 atypical neuroleptic drugs compared with haloperidol and placebo. *Journal of clinical psychopharmacology* 2011; 31(6): 698-704.

187. Kumar A, Hozo I, Wheatley K, Djulbegovic B. Thalidomide versus bortezomib based regimens as first-line therapy for patients with multiple myeloma: a systematic review. *American journal of hematology* 2011; 86(1): 18-24.

188. Launois R, Avouac B, Berenbaum F, et al. Comparison of certolizumab pegol with other anticytokine agents for treatment of rheumatoid arthritis: a multiple-treatment Bayesian metaanalysis. *The Journal of rheumatology* 2011; 38(5): 835-45.

189. Leung HW, Chan AL. Multikinase inhibitors in metastatic renal cell carcinoma: indirect comparison meta-analysis. *Clin Ther* 2011; 33(6): 708-16.

190. Levy AR, Johnston KM, Sambrook J, et al. Indirect comparison of the efficacy of cetuximab and cisplatin in squamous cell carcinoma of the head and neck. *Curr Med Res Opin* 2011; 27(12): 2253-9.

191. Lockhart IA, Orme ME, Mitchell SA. The efficacy of licensed-indication use of donepezil and memantine monotherapies for treating behavioural and psychological symptoms of dementia in patients with Alzheimer's disease: systematic review and meta-analysis. *Dementia and geriatric cognitive disorders extra* 2011; 1(1): 212-27.

192. Loke YK, Kwok CS. Dabigatran and rivaroxaban for prevention of venous thromboembolism--systematic review and adjusted indirect comparison. *J Clin Pharm Ther* 2011; 36(1): 111-24.

193. Makani H, Bangalore S, Romero J, Wever-Pinzon O, Messerli FH. Effect of renin-angiotensin system blockade on calcium channel blocker-associated peripheral edema. *The American journal of medicine* 2011; 124(2): 128-35.

194. Manzoli L, De Vito C, Salanti G, D'Addario M, Villari P, Ioannidis JP. Meta-analysis of the immunogenicity and tolerability of pandemic influenza A 2009 (H1N1) vaccines. *Plos One* 2011; 6(9): e24384.

195. Mariani J, Macchia A, Belziti C, et al. Noninvasive ventilation in acute cardiogenic pulmonary edema: a meta-analysis of randomized controlled trials. *Journal of cardiac failure* 2011; 17(10): 850-9.

196. Maund E, McDaid C, Rice S, Wright K, Jenkins B, Woolacott N. Paracetamol and selective and non-selective non-steroidal anti-inflammatory drugs for the reduction in morphine-related side-effects after major surgery: a systematic review. *Br J Anaesth* 2011; 106(3): 292-7.

197. McIntosh B, Cameron C, Singh SR, et al. Second-line therapy in patients with type 2 diabetes inadequately controlled with metformin monotherapy: a systematic review and mixed-treatment comparison meta-analysis. *Open medicine : a peer-reviewed, independent, open-access journal* 2011; 5(1): e35-48.

198. Mickisch GH, Schwander B, Escudier B, et al. Indirect treatment comparison of bevacizumab + interferon-alpha-2a vs tyrosine kinase inhibitors in first-line metastatic renal cell carcinoma therapy. *ClinicoEconomics and outcomes research : CEOR* 2011; 3: 19-27.

199. Mills EJ, Druyts E, Ghement I, Puhan MA. Pharmacotherapies for chronic obstructive pulmonary disease: a multiple treatment comparison meta-analysis. *Clin Epidemiol* 2011; 3: 107-29.

200. Mills EJ, Wu P, Chong G, et al. Efficacy and safety of statin treatment for cardiovascular disease: a network meta-analysis of 170,255 patients from 76 randomized trials. *QJM* 2011; 104(2): 109-24.

201. Nuijten MJ, Aultman R, Carpeno Jde C, et al. An indirect comparison of the efficacy of bevacizumab plus carboplatin and paclitaxel versus pemetrexed with cisplatin in patients with advanced or recurrent non-squamous adenocarcinoma non-small cell lung cancer. *Curr Med Res Opin* 2011; 27(11): 2193-201.

202. Numthavaj P, Thakkinstian A, Dejthevaporn C, Attia J. Corticosteroid and antiviral therapy for Bell's palsy: a network meta-analysis. *BMC neurology* 2011; 11: 1.

203. Padwal R, Klarenbach S, Wiebe N, et al. Bariatric surgery: a systematic review and network meta-analysis of randomized trials. *Obesity reviews : an official journal of the International Association for the Study of Obesity* 2011; 12(8): 602-21.

204. Phung OJ, Kahn SR, Cook DJ, Murad MH. Dosing frequency of unfractionated heparin thromboprophylaxis: a meta-analysis. *Chest* 2011; 140(2): 374-81.

205. Phung OJ, Sood NA, Sill BE, Coleman CI. Oral anti-diabetic drugs for the prevention of Type 2 diabetes. *Diabetic medicine : a journal of the British Diabetic Association* 2011; 28(8): 948-64.

206. Rheims S, Perucca E, Cucherat M, Ryvlin P. Factors determining response to antiepileptic drugs in randomized controlled trials. A systematic review and meta-analysis. *Epilepsia* 2011; 52(2): 219-33.

207. Riemsma R, Forbes C, Harker J, et al. Systematic review of tapentadol in chronic severe pain (Structured abstract). *Current Medical Research and Opinion*, 2011. http://onlinelibrary.wiley.com/o/cochrane/cldare/articles/DARE-12011006326/frame.html (accessed.

208. Rodgers M, Soares M, Epstein D, Yang H, Fox D, Eastwood A. Bevacizumab in combination with a taxane for the first-line treatment of HER2-negative metastatic breast cancer (Structured abstract). *Health Technology Assessment Database*, 2011. http://onlinelibrary.wiley.com/o/cochrane/clhta/articles/HTA-32011000768/frame.html (accessed.

209. Roskell NS, Beard SM, Zhao Y, Le TK. A meta-analysis of pain response in the treatment of fibromyalgia. *Pain practice : the official journal of World Institute of Pain* 2011; 11(6): 516-27.

210. Salliot C, Finckh A, Katchamart W, et al. Indirect comparisons of the efficacy of biological antirheumatic agents in rheumatoid arthritis in patients with an inadequate response to conventional disease-modifying antirheumatic drugs or to an anti-tumour necrosis factor agent: a meta-analysis. *Ann Rheum Dis* 2011; 70(2): 266-71.

211. Sanches AC, Correr CJ, Venson R, Pontarolo R. Revisiting the efficacy of long-acting insulin analogues on adults with type 1 diabetes using mixed-treatment comparisons. *Diabetes Res Clin Pract* 2011; 94(3): 333-9.

212. Sciarretta S, Palano F, Tocci G, Baldini R, Volpe M. Antihypertensive treatment and development of heart failure in hypertension: a Bayesian network meta-analysis of studies in patients with hypertension and high cardiovascular risk. *Archives of internal medicine* 2011; 171(5): 384-94.

213. Singh JA, Wells GA, Christensen R, et al. Adverse effects of biologics: a network meta-analysis and Cochrane overview. *The Cochrane database of systematic reviews* 2011; (2): CD008794.

214. Sjoquist KM, Burmeister BH, Smithers BM, et al. Survival after neoadjuvant chemotherapy or chemoradiotherapy for resectable oesophageal carcinoma: an updated meta-analysis. *The lancet oncology* 2011; 12(7): 681-92.

215. Smith B, Peterson K, Fu R, McDonagh M, Thakurta S. Drug Class Review: Drugs for Fibromyalgia: Final Original Report. Portland OR: Oregon Health & Science University.; 2011.

216. Squires H, Simpson E, Meng Y, et al. A systematic review and economic evaluation of cilostazol, naftidrofuryl oxalate, pentoxifylline and inositol nicotinate for the treatment of intermittent claudication in people with peripheral arterial disease. *Health technology assessment (Winchester, England)* 2011; 15(40): 1-210.

217. Stowe R, Ives N, Clarke CE, et al. Meta-analysis of the comparative efficacy and safety of adjuvant treatment to levodopa in later Parkinson's disease. *Movement disorders : official journal of the Movement Disorder Society* 2011; 26(4): 587-98.

218. Tosh JC, Wailoo AJ, Scott DL, Deighton CM. Cost-effectiveness of combination nonbiologic disease-modifying antirheumatic drug strategies in patients with early rheumatoid arthritis. *The Journal of rheumatology* 2011; 38(8): 1593-600.

219. Trelle S, Reichenbach S, Wandel S, et al. Cardiovascular safety of non-steroidal anti-inflammatory drugs: network meta-analysis. *BMJ (Clinical research ed)* 2011; 342: c7086.

220. Tropeano AI, Saleh N, Hawajri N, Macquin-Mavier I, Maison P. Do all antihypertensive drugs improve carotid intima-media thickness? A network meta-analysis of randomized controlled trials. *Fundamental & clinical pharmacology* 2011; 25(3): 395-404.

221. Turkstra E, Ng SK, Scuffham PA. A mixed treatment comparison of the short-term efficacy of biologic disease modifying anti-rheumatic drugs in established rheumatoid arthritis. *Curr Med Res Opin* 2011; 27(10): 1885-97.

222. van de Kerkhof P, de Peuter R, Ryttov J, Jansen JP. Mixed treatment comparison of a two-compound formulation (TCF) product containing calcipotriol and betamethasone dipropionate with other topical treatments in psoriasis vulgaris. *Curr Med Res Opin* 2011; 27(1): 225-38.

223. Van den Bruel A, Gailly J, Devriese S, Welton NJ, Shortt AJ, Vrijens F. The protective effect of ophthalmic viscoelastic devices on endothelial cell loss during cataract surgery: a meta-analysis using mixed treatment comparisons. *The British journal of ophthalmology* 2011; 95(1): 5-10.

224. Vieira MC, Kumar RN, Jansen JP. Comparative effectiveness of efavirenz, protease inhibitors, and raltegravir-based regimens as first-line treatment for HIV-infected adults: a mixed treatment comparison. *HIV clinical trials* 2011; 12(4): 175-89.

225. Virgili G, Novielli N, Menchini F, Murro V, Giacomelli G. Pharmacological treatments for neovascular age-related macular degeneration: can mixed treatment comparison meta-analysis be useful? *Current drug targets* 2011; 12(2): 212-20.

226. Vissers DC, Lenre M, Tolley K, Jakobsson J, Sendersky V, Jansen JP. An economic evaluation of short-acting opioids for treatment of breakthrough pain in patients with cancer. *Value in health : the journal of the International Society for Pharmacoeconomics and Outcomes Research* 2011; 14(2): 274-81.

227. Vlaar PJ, Mahmoud KD, Holmes DR, Jr., et al. Culprit vessel only versus multivessel and staged percutaneous coronary intervention for multivessel disease in patients presenting with ST-segment elevation myocardial infarction: a pairwise and network meta-analysis. *J Am Coll Cardiol* 2011; 58(7): 692-703.

228. Wiebe N, Padwal R, Field C, Marks S, Jacobs R, Tonelli M. A systematic review on the effect of sweeteners on glycemic response and clinically relevant outcomes. *BMC Med* 2011; 9: 123.

229. Wolff RF, Bala MM, Westwood M, Kessels AG, Kleijnen J. 5% lidocaine-medicated plaster vs other relevant interventions and placebo for post-herpetic neuralgia (PHN): a systematic review. *Acta neurologica Scandinavica* 2011; 123(5): 295-309.

230. Zeng L, Luo R, Zhang L. Efficacy of high-dose ACTH versus low-dose ACTH in infantile spasms: A meta-analysis with direct and indirect comparison of randomized trials. *Journal of Pediatric Neurology* 2011; 9(2): 141-9.

231. Ziogas DC, Voulgarelis M, Zintzaras E. A network meta-analysis of randomized controlled trials of induction treatments in acute myeloid leukemia in the elderly. *Clin Ther* 2011; 33(3): 254-79.

232. Alberton M, Wu P, Druyts E, Briel M, Mills EJ. Adverse events associated with individual statin treatments for cardiovascular disease: an indirect comparison meta-analysis. *QJM* 2012; 105(2): 145-57.

233. Alkhafaji AA, Trinquart L, Baron G, Desvarieux M, Ravaud P. Impact of evergreening on patients and health insurance: a meta analysis and reimbursement cost analysis of citalopram/escitalopram antidepressants. *BMC Med* 2012; 10: 142.

234. Alonso-Coello P, Zhou Q, Guyatt G. Home-monitoring of oral anticoagulation vs. dabigatran An indirect comparison. *Thrombosis and Haemostasis* 2012; 108(4): 647-53.

235. Ara R, Blake L, Gray L, et al. What is the clinical effectiveness and cost-effectiveness of using drugs in treating obese patients in primary care? A systematic review. *Health technology assessment (Winchester, England)* 2012; 16(5): iii-xiv, 1-195.

236. Ara R, Pandor A, Stevens J, et al. Prescribing high-dose lipid-lowering therapy early to avoid subsequent cardiovascular events: is this a cost-effective strategy? *European journal of preventive cardiology* 2012; 19(3): 474-83.

237. Asseburg C, Peura P, Oksanen T, Turunen J, Purmonen T, Martikainen J. Cost-effectiveness of oral triptans for acute migraine: mixed treatment comparison. *International journal of technology assessment in health care* 2012; 28(4): 382-9.

238. Baker EL, Coleman CI, Reinhart KM, et al. Effect of Biologic Agents on Non-PASI Outcomes in Moderate-to-Severe Plaque Psoriasis: Systematic Review and Meta-Analyses. *Dermatology and therapy* 2012; 2(1): 9.

239. Baker WL, Phung OJ. Systematic review and adjusted indirect comparison meta-analysis of oral anticoagulants in atrial fibrillation. *Circulation Cardiovascular quality and outcomes* 2012; 5(5): 711-9.

240. Bally M, Dendukuri N, Sinclair A, Ahern SP, Poisson M, Brophy J. A network meta-analysis of antibiotics for treatment of hospitalised patients with suspected or proven meticillin-resistant Staphylococcus aureus infection. *International journal of antimicrobial agents* 2012; 40(6): 479-95.

241. Bangalore S, Kumar S, Fusaro M, et al. Short- and long-term outcomes with drug-eluting and bare-metal coronary stents: a mixed-treatment comparison analysis of 117 762 patient-years of follow-up from randomized trials. *Circulation* 2012; 125(23): 2873-91.

242. Bangalore S, Kumar S, Fusaro M, et al. Outcomes with various drug eluting or bare metal stents in patients with diabetes mellitus: mixed treatment comparison analysis of 22,844 patient years of follow-up from randomised trials. *BMJ (Clinical research ed)* 2012; 345: e5170.

243. Bash LD, Buono JL, Davies GM, et al. Systematic review and meta-analysis of the efficacy of cardioversion by vernakalant and comparators in patients with atrial fibrillation. *Cardiovascular drugs and therapy / sponsored by the International Society of Cardiovascular Pharmacotherapy* 2012; 26(2): 167-79.

244. Bond M, Rogers G, Peters J, et al. The effectiveness and cost-effectiveness of donepezil, galantamine, rivastigmine and memantine for the treatment of Alzheimer's disease (review of Technology Appraisal No. 111): a systematic review and economic model. *Health Technology Assessment* 2012; 16(21): 1-+.

245. Bracale U, Melillo P, Pignata G, et al. Which is the best laparoscopic approach for inguinal hernia repair: TEP or TAPP? A systematic review of the literature with a network meta-analysis. *Surgical endoscopy* 2012; 26(12): 3355-66.

246. Buser N, Ivic S, Kessler TM, Kessels AG, Bachmann LM. Efficacy and adverse events of antimuscarinics for treating overactive bladder: network meta-analyses. *Eur Urol* 2012; 62(6): 1040-60.

247. Caldeira D, Alarcao J, Vaz-Carneiro A, Costa J. Risk of pneumonia associated with use of angiotensin converting enzyme inhibitors and angiotensin receptor blockers: systematic review and meta-analysis. *BMJ (Clinical research ed)* 2012; 345: e4260.

248. Carter B, Fedorowicz Z. Antiemetic treatment for acute gastroenteritis in children: an updated Cochrane systematic review with meta-analysis and mixed treatment comparison in a Bayesian framework. *Bmj Open* 2012; 2(4).

249. Casey ET, Murad MH, Zumaeta-Garcia M, et al. Treatment of acute iliofemoral deep vein thrombosis. *J Vasc Surg* 2012; 55(5): 1463-73.

250. Cassese S, Ndrepepa G, King LA, Tada T, Fusaro M, Kastrati A. Two zotarolimus-eluting stent generations: a meta-analysis of 12 randomised trials versus other limus-eluting stents and an adjusted indirect comparison. *Heart* 2012; 98(22): 1632-40.

251. Chang KV, Chen SY, Chen WS, Tu YK, Chien KL. Comparative effectiveness of focused shock wave therapy of different intensity levels and radial shock wave therapy for treating plantar fasciitis: a systematic review and network meta-analysis. *Archives of physical medicine and rehabilitation* 2012; 93(7): 1259-68.

252. Chen YF, Madan J, Welton N, et al. Effectiveness and cost-effectiveness of computer and other electronic aids for smoking cessation: a systematic review and network meta-analysis. *Health technology assessment (Winchester, England)* 2012; 16(38): 1-205, iii-v.

253. Cheng MM, Goulart B, Veenstra DL, Blough DK, Devine EB. A network meta-analysis of therapies for previously untreated chronic lymphocytic leukemia. *Cancer Treat Rev* 2012; 38(8): 1004-11.

254. Cohen A, Drost P, Marchant N, et al. The efficacy and safety of pharmacological prophylaxis of venous thromboembolism following elective knee or hip replacement: systematic review and network meta-analysis. *Clinical and applied thrombosis/hemostasis : official journal of the International Academy of Clinical and Applied Thrombosis/Hemostasis* 2012; 18(6): 611-27.

255. Cohen D, Bonnot O, Bodeau N, Consoli A, Laurent C. Adverse effects of second-generation antipsychotics in children and adolescents: a Bayesian meta-analysis (Structured abstract). *Database of Abstracts of Reviews of Effects*, 2012. http://onlinelibrary.wiley.com/o/cochrane/cldare/articles/DARE-12012027166/frame.html (accessed.

256. Coleman KA, Xavier VY, Palmer TL, Meaney JV, Radalj LM, Canny LM. An indirect comparison of the efficacy and safety of desvenlafaxine and venlafaxine using placebo as the common comparator. *CNS spectrums* 2012; 17(3): 131-41.

257. Cooper CL, Druyts E, Thorlund K, et al. Boceprevir and telaprevir for the treatment of chronic hepatitis C genotype 1 infection: an indirect comparison meta-analysis. *Therapeutics and clinical risk management* 2012; 8: 105-30.

258. Cooper KL, Fitzgerald P, Dillingham K, Helme K, Akehurst R. Romiplostim and eltrombopag for immune thrombocytopenia: methods for indirect comparison. *International journal of technology assessment in health care* 2012; 28(3): 249-58.

259. Cooper NJ, Kendrick D, Achana F, et al. Network meta-analysis to evaluate the effectiveness of interventions to increase the uptake of smoke alarms. *Epidemiologic reviews* 2012; 34(1): 32-45.

260. Cope S, Capkun-Niggli G, Gale R, et al. Efficacy of once-daily indacaterol relative to alternative bronchodilators in COPD: a patient-level mixed treatment comparison. *Value in health : the journal of the International Society for Pharmacoeconomics and Outcomes Research* 2012; 15(3): 524-33.

261. Cope S, Kraemer M, Zhang J, Capkun-Niggli G, Jansen JP. Efficacy of indacaterol 75 mug versus fixed-dose combinations of formoterol-budesonide or salmeterol-fluticasone for COPD: a network meta-analysis. *Int J Chron Obstruct Pulmon Dis* 2012; 7: 415-20.

262. Cope S, Zhang J, Williams J, Jansen JP. Efficacy of once-daily indacaterol 75 mug relative to alternative bronchodilators in COPD: a study level and a patient level network meta-analysis. *BMC Pulm Med* 2012; 12: 29.

263. Corcoran T, Rhodes JE, Clarke S, Myles PS, Ho KM. Perioperative fluid management strategies in major surgery: a stratified meta-analysis. *Anesthesia and analgesia* 2012; 114(3): 640-51.

264. Cure S, Diels J, Gavart S, Bianic F, Jones E. Efficacy of telaprevir and boceprevir in treatment-naive and treatment-experienced genotype 1 chronic hepatitis C patients: an indirect comparison using Bayesian network meta-analysis. *Curr Med Res Opin* 2012; 28(11): 1841-56.

265. Daniels JP, Middleton LJ, Champaneria R, et al. Second generation endometrial ablation techniques for heavy menstrual bleeding: network meta-analysis. *BMJ (Clinical research ed)* 2012; 344: e2564.

266. Del Santo F, Maratea D, Fadda V, Trippoli S, Messori A. Treatments for relapsing-remitting multiple sclerosis: summarising current information by network meta-analysis. *Eur J Clin Pharmacol* 2012; 68(4): 441-8.

267. Dequen P, Lorigan P, Jansen JP, van Baardewijk M, Ouwens MJ, Kotapati S. Systematic review and network meta-analysis of overall survival comparing 3 mg/kg ipilimumab with alternative therapies in the management of pretreated patients with unresectable stage III or IV melanoma. *Oncologist* 2012; 17(11): 1376-85.

268. Desai RJ, Hansen RA, Rao JK, et al. Mixed treatment comparison of the treatment discontinuations of biologic disease-modifying antirheumatic drugs in adults with rheumatoid arthritis. *The Annals of pharmacotherapy* 2012; 46(11): 1491-505.

269. Dewilde S, Hawkins N. Investigating incoherence gives insight: clopidogrel is equivalent to extended-release dipyridamole plus aspirin in secondary stroke prevention. *J Clin Epidemiol* 2012; 65(8): 835-45.

270. Donahue KE, Jonas DE, Hansen RA, et al. Drug Therapy for Rheumatoid Arthritis in Adults: An Update. Rockville MD; 2012.

271. Dranitsaris G, Jelincic V, Choe Y. Meta-regression analysis to indirectly compare prophylaxis with dalteparin or enoxaparin in patients at high risk for venous thromboembolic events. *Clinical and applied thrombosis/hemostasis : official journal of the International Academy of Clinical and Applied Thrombosis/Hemostasis* 2012; 18(3): 233-42.

272. Dumville JC, Soares MO, O'Meara S, Cullum N. Systematic review and mixed treatment comparison: dressings to heal diabetic foot ulcers. *Diabetologia* 2012; 55(7): 1902-10.

273. Dunkley AJ, Charles K, Gray LJ, Camosso-Stefinovic J, Davies MJ, Khunti K. Effectiveness of interventions for reducing diabetes and cardiovascular disease risk in people with metabolic syndrome: systematic review and mixed treatment comparison meta-analysis. *Diabetes, obesity & metabolism* 2012; 14(7): 616-25.

274. Ernest PJ, Viechtbauer W, Schouten JS, et al. The influence of the assessment method on the incidence of visual field progression in glaucoma: a network meta-analysis. *Acta ophthalmologica* 2012; 90(1): 10-9.

275. Favalli EG, Pregnolato F, Biggioggero M, Meroni PL. The role of biologic agents in damage progression in rheumatoid arthritis: Indirect comparison of data coming from randomized clinical trials. *Therapeutic Advances in Musculoskeletal Disease* 2012; 4(4): 213-23.

276. Ford JA, Elders A, Shyangdan D, Royle P, Waugh N. The relative clinical effectiveness of ranibizumab and bevacizumab in diabetic macular oedema: an indirect comparison in a systematic review (Structured abstract). *BMJ (Clinical research ed)*, 2012. http://onlinelibrary.wiley.com/o/cochrane/cldare/articles/DARE-12012037128/frame.html (accessed.

277. Fortin P, Mintzes B, Innes M. A systematic review of intravitreal bevacizumab for the treatment of diabetic macular edema (Structured abstract). *Health Technology Assessment Database*, 2012. http://onlinelibrary.wiley.com/o/cochrane/clhta/articles/HTA-32012000710/frame.html (accessed.

278. Fox BD, Kahn SR, Langleben D, Eisenberg MJ, Shimony A. Efficacy and safety of novel oral anticoagulants for treatment of acute venous thromboembolism: direct and adjusted indirect meta-analysis of randomised controlled trials. *BMJ (Clinical research ed)* 2012; 345: e7498.

279. Fretheim A, Odgaard-Jensen J, Brors O, et al. Comparative effectiveness of antihypertensive medication for primary prevention of cardiovascular disease: systematic review and multiple treatments meta-analysis. *BMC Med* 2012; 10: 33.

280. Gallego-Galisteo M, Villa-Rubio A, Alegre-del Rey E, Marquez-Fernandez E, Ramos-Baez JJ. Indirect comparison of biological treatments in refractory rheumatoid arthritis. *J Clin Pharm Ther* 2012; 37(3): 301-7.

281. Gomez-Outes A, Terleira-Fernandez AI, Suarez-Gea ML, Vargas-Castrillon E. Dabigatran, rivaroxaban, or apixaban versus enoxaparin for thromboprophylaxis after total hip or knee replacement: systematic review, meta-analysis, and indirect treatment comparisons. *BMJ (Clinical research ed)* 2012; 344: e3675.

282. Gray LJ, Cooper N, Dunkley A, et al. A systematic review and mixed treatment comparison of pharmacological interventions for the treatment of obesity. *Obesity reviews : an official journal of the International Association for the Study of Obesity* 2012; 13(6): 483-98.

283. Guyot P, Taylor PC, Christensen R, et al. Indirect treatment comparison of abatacept with methotrexate versus other biologic agents for active rheumatoid arthritis despite methotrexate therapy in the United kingdom. *The Journal of rheumatology* 2012; 39(6): 1198-206.

284. Haas DM, Caldwell DM, Kirkpatrick P, McIntosh JJ, Welton NJ. Tocolytic therapy for preterm delivery: systematic review and network meta-analysis. *BMJ (Clinical research ed)* 2012; 345: e6226.

285. Harenberg J, Marx S, Dahl OE, et al. Interpretation of endpoints in a network meta-analysis of new oral anticoagulants following total hip or total knee replacement surgery. *Thromb Haemost* 2012; 108(5): 903-12.

286. Harenberg J, Marx S, Diener HC, et al. Comparison of efficacy and safety of dabigatran, rivaroxaban and apixaban in patients with atrial fibrillation using network meta-analysis. *International angiology : a journal of the International Union of Angiology* 2012; 31(4): 330-9.

287. Hutton B, Joseph L, Fergusson D, Mazer CD, Shapiro S, Tinmouth A. Risks of harms using antifibrinolytics in cardiac surgery: systematic review and network meta-analysis of randomised and observational studies. *BMJ (Clinical research ed)* 2012; 345: e5798.

288. Jost WH, Friede M, Schnitker J. Indirect meta-analysis of randomised placebo-controlled clinical trials on rasagiline and selegiline in the symptomatic treatment of Parkinson's disease (Structured abstract). *Database of Abstracts of Reviews of Effects*, 2012. http://onlinelibrary.wiley.com/o/cochrane/cldare/articles/DARE-12013008870/frame.html (accessed.

289. Kansal AR, Sharma M, Bradley-Kennedy C, et al. Dabigatran versus rivaroxaban for the prevention of stroke and systemic embolism in atrial fibrillation in Canada. Comparative efficacy and cost-effectiveness. *Thromb Haemost* 2012; 108(4): 672-82.

290. Knottnerus BJ, Grigoryan L, Geerlings SE, et al. Comparative effectiveness of antibiotics for uncomplicated urinary tract infections: network meta-analysis of randomized trials. *Family practice* 2012; 29(6): 659-70.

291. Kruidenier LM, Viechtbauer W, Nicolai SP, Buller H, Prins MH, Teijink JA. Treatment for intermittent claudication and the effects on walking distance and quality of life. *Vascular* 2012; 20(1): 20-35.

292. Kwok CS, Arthur AK, Anibueze CI, Singh S, Cavallazzi R, Loke YK. Risk of Clostridium difficile infection with acid suppressing drugs and antibiotics: meta-analysis. *The American journal of gastroenterology* 2012; 107(7): 1011-9.

293. Lang SH, Manning N, Armstrong N, et al. Treatment with tirofiban for acute coronary syndrome (ACS): a systematic review and network analysis. *Curr Med Res Opin* 2012; 28(3): 351-70.

294. Lee EK, Revil C, Ngoh CA, et al. Clinical and cost effectiveness of bevacizumab + FOLFIRI combination versus FOLFIRI alone as first-line treatment of metastatic colorectal cancer in South Korea. *Clin Ther* 2012; 34(6): 1408-19.

295. Liao WC, Tu YK, Wu MS, et al. Balloon dilation with adequate duration is safer than sphincterotomy for extracting bile duct stones: a systematic review and meta-analyses. *Clinical gastroenterology and hepatology : the official clinical practice journal of the American Gastroenterological Association* 2012; 10(10): 1101-9.

296. Lin VW, Ringold S, Devine EB. Comparison of Ustekinumab With Other Biological Agents for the Treatment of Moderate to Severe Plaque Psoriasis: A Bayesian Network Meta-analysis. *Archives of dermatology* 2012; 148(12): 1403-10.

297. Lip GHY, Kongnakorn T, Phatak H, et al. Cost-effectiveness of apixaban against other novel oral anticoagulants (NOACs) for stroke prevention in atrial fibrillation patients. *European Heart Journal* 2012; 33: 54.

298. Lip GYH, Larsen TB, Skjoth F, Rasmussen LH. Indirect Comparisons of New Oral Anticoagulant Drugs for Efficacy and Safety When Used for Stroke Prevention in Atrial Fibrillation. *Journal of the American College of Cardiology* 2012; 60(8): 738-46.

299. Littlewood KJ, Higashi K, Jansen JP, et al. A network meta-analysis of the efficacy of inhaled antibiotics for chronic Pseudomonas infections in cystic fibrosis. *Journal of cystic fibrosis : official journal of the European Cystic Fibrosis Society* 2012; 11(5): 419-26.

300. Liu SC, Tu YK, Chien MN, Chien KL. Effect of antidiabetic agents added to metformin on glycaemic control, hypoglycaemia and weight change in patients with type 2 diabetes: a network meta-analysis. *Diabetes, obesity & metabolism* 2012; 14(9): 810-20.

301. Liu Y, Wu EQ, Bensimon AG, et al. Cost per responder associated with biologic therapies for Crohn's disease, psoriasis, and rheumatoid arthritis. *Advances in therapy* 2012; 29(7): 620-34.

302. Mak KH. Coronary and mortality risk of novel oral antithrombotic agents: a meta-analysis of large randomised trials. *Bmj Open* 2012; 2(5).

303. Martyn-St James M, Glanville J, McCool R, et al. The efficacy and safety of retigabine and other adjunctive treatments for refractory partial epilepsy: a systematic review and indirect comparison. *Seizure : the journal of the British Epilepsy Association* 2012; 21(9): 665-78.

304. Maund E, Craig D, Suekarran S, et al. Management of frozen shoulder: a systematic review and cost-effectiveness analysis. *Health technology assessment (Winchester, England)* 2012; 16(11): 1-264.

305. McIntosh B, Cameron C, Singh SR, Yu C, Dolovich L, Houlden R. Choice of therapy in patients with type 2 diabetes inadequately controlled with metformin and a sulphonylurea: a systematic review and mixed-treatment comparison meta-analysis. *Open medicine : a peer-reviewed, independent, open-access journal* 2012; 6(2): e62-74.

306. Mhaskar R, Redzepovic J, Wheatley K, et al. Bisphosphonates in multiple myeloma: a network meta-analysis. *The Cochrane database of systematic reviews* 2012; 5: CD003188.

307. Migliore A, Broccoli S, Bizzi E, Lagana B. Indirect comparison of the effects of anti-TNF biological agents in patients with ankylosing spondylitis by means of a mixed treatment comparison performed on efficacy data from published randomised, controlled trials. *Journal of medical economics* 2012; 15(3): 473-80.

308. Migliore A, Broccoli S, Massafra U, Bizzi E, Frediani B. Mixed-treatment comparison of anabolic (teriparatide and PTH 1-84) therapies in women with severe osteoporosis. *Curr Med Res Opin* 2012; 28(3): 467-73.

309. Mills EJ, Wu P, Lockhart I, Thorlund K, Puhan M, Ebbert JO. Comparisons of high-dose and combination nicotine replacement therapy, varenicline, and bupropion for smoking cessation: a systematic review and multiple treatment meta-analysis. *Ann Med* 2012; 44(6): 588-97.

310. Morgan LC, Gartlehner G, Richard HA. Comparative effectiveness of second generation antidepressants in the pharmacologic treatment of adult depression. *European Psychiatry* 2012; 27.

311. Murad MH, Drake MT, Mullan RJ, et al. Clinical review. Comparative effectiveness of drug treatments to prevent fragility fractures: a systematic review and network meta-analysis. *The Journal of clinical endocrinology and metabolism* 2012; 97(6): 1871-80.

312. Orme ME, Macgilchrist KS, Mitchell S, Spurden D, Bird A. Systematic review and network meta-analysis of combination and monotherapy treatments in disease-modifying antirheumatic drug-experienced patients with rheumatoid arthritis: analysis of American College of Rheumatology criteria scores 20, 50, and 70. *Biologics : targets & therapy* 2012; 6: 429-64.

313. Palmerini T, Biondi-Zoccai G, Della Riva D, et al. Stent thrombosis with drug-eluting and bare-metal stents: evidence from a comprehensive network meta-analysis. *Lancet* 2012; 379(9824): 1393-402.

314. Perez RU, Castillo Munoz MA, Navarro Caballero JA, Marquez Pelaez S. Protease inhibitors (Boceprevir and telaprevir) in the treatment of chronic HCV infection: relative efficacy, safety and efficiency (Structured abstract). *Health Technology Assessment Database*, 2012. http://onlinelibrary.wiley.com/o/cochrane/clhta/articles/HTA-32012000838/frame.html (accessed.

315. Pichenot M, Deuffic-Burban S, Cuzin L, Yazdanpanah Y. Efficacy of new antiretroviral drugs in treatment-experienced HIV-infected patients: a systematic review and meta-analysis of recent randomized controlled trials. *HIV medicine* 2012; 13(3): 148-55.

316. Qi WX, Tang LN, He AN, Shen Z, Lin F, Yao Y. Erlotinib and pemetrexed as maintenance therapy for advanced non-small-cell lung cancer: a systematic review and indirect comparison. *Curr Med Res Opin* 2012; 28(4): 643-50.

317. Ramsberg J, Asseburg C, Henriksson M. Effectiveness and cost-effectiveness of antidepressants in primary care: a multiple treatment comparison meta-analysis and cost-effectiveness model. *Plos One* 2012; 7(8): e42003.

318. Rasmussen LH, Larsen TB, Graungaard T, Skjoth F, Lip GY. Primary and secondary prevention with new oral anticoagulant drugs for stroke prevention in atrial fibrillation: indirect comparison analysis. *BMJ (Clinical research ed)* 2012; 345: e7097.

319. Reich K, Burden AD, Eaton JN, Hawkins NS. Efficacy of biologics in the treatment of moderate to severe psoriasis: a network meta-analysis of randomized controlled trials. *The British journal of dermatology* 2012; 166(1): 179-88.

320. Riemsma R, Forbes CA, Amonkar MM, et al. Systematic review of lapatinib in combination with letrozole compared with other first-line treatments for hormone receptor positive(HR+) and HER2+ advanced or metastatic breast cancer(MBC). *Curr Med Res Opin* 2012; 28(8): 1263-79.

321. Roskell NS, Zimovetz EA, Rycroft CE, Eckert BJ, Tyas DA. Annualized relapse rate of first-line treatments for multiple sclerosis: a meta-analysis, including indirect comparisons versus fingolimod. *Curr Med Res Opin* 2012; 28(5): 767-80.

322. Roush GC, Holford TR, Guddati AK. Chlorthalidone compared with hydrochlorothiazide in reducing cardiovascular events: systematic review and network meta-analyses. *Hypertension* 2012; 59(6): 1110-7.

323. Schmitz S, Adams R, Walsh CD, Barry M, FitzGerald O. A mixed treatment comparison of the efficacy of anti-TNF agents in rheumatoid arthritis for methotrexate non-responders demonstrates differences between treatments: a Bayesian approach. *Ann Rheum Dis* 2012; 71(2): 225-30.

324. Schmucker C, Ehlken C, Agostini HT, et al. A safety review and meta-analyses of bevacizumab and ranibizumab: off-label versus goldstandard. *Plos One* 2012; 7(8): e42701.

325. Schoels M, Aletaha D, Smolen JS, Wong JB. Comparative effectiveness and safety of biological treatment options after tumour necrosis factor alpha inhibitor failure in rheumatoid arthritis: systematic review and indirect pairwise meta-analysis (Structured abstract). *Annals of the Rheumatic Diseases*, 2012. http://onlinelibrary.wiley.com/o/cochrane/cldare/articles/DARE-12012035687/frame.html (accessed.

326. Soini EJ, Hallinen TA, Puolakka K, Vihervaara V, Kauppi MJ. Cost-effectiveness of adalimumab, etanercept, and tocilizumab as first-line treatments for moderate-to-severe rheumatoid arthritis. *Journal of medical economics* 2012; 15(2): 340-51.

327. Stam W, Jansen J, Taylor S. Efficacy of etoricoxib, celecoxib, lumiracoxib, non-selective NSAIDs, and acetaminophen in osteoarthritis: a mixed treatment comparison. *The open rheumatology journal* 2012; 6: 6-20.

328. Steiner S, Moertl D, Chen L, Coyle D, Wells GA. Network meta-analysis of prasugrel, ticagrelor, high- and standard-dose clopidogrel in patients scheduled for percutaneous coronary interventions. *Thromb Haemost* 2012; 108(2): 318-27.

329. Stevens JW, Simpson E, Harnan S, et al. Systematic review of the efficacy of cilostazol, naftidrofuryl oxalate and pentoxifylline for the treatment of intermittent claudication. *The British journal of surgery* 2012; 99(12): 1630-8.

330. Sun F, Yu K, Wu S, et al. Cardiovascular safety and glycemic control of glucagon-like peptide-1 receptor agonists for type 2 diabetes mellitus: a pairwise and network meta-analysis. *Diabetes Res Clin Pract* 2012; 98(3): 386-95.

331. Sun F, Yu K, Yang Z, et al. Impact of GLP-1 receptor agonists on major gastrointestinal disorders for type 2 diabetes mellitus: a mixed treatment comparison meta-analysis. *Experimental diabetes research* 2012; 2012: 230624.

332. Szegedi A, Verweij P, van Duijnhoven W, Mackle M, Cazorla P, Fennema H. Meta-analyses of the efficacy of asenapine for acute schizophrenia: comparisons with placebo and other antipsychotics. *The Journal of clinical psychiatry* 2012; 73(12): 1533-40.

333. Tang DH, Malone DC. A network meta-analysis on the efficacy of serotonin type 3 receptor antagonists used in adults during the first 24 hours for postoperative nausea and vomiting prophylaxis. *Clin Ther* 2012; 34(2): 282-94.

334. Testa L, Agnifili M, Latini RA, et al. Adjusted indirect comparison of new oral anticoagulants for stroke prevention in atrial fibrillation. *QJM* 2012; 105(10): 949-57.

335. Thakkinstian A, Attia J, Anothaisintawee T, Nickel JC. alpha-blockers, antibiotics and anti-inflammatories have a role in the management of chronic prostatitis/chronic pelvic pain syndrome. *BJU international* 2012; 110(7): 1014-22.

336. Thorlund K, Druyts E, Avina-Zubieta JA, Mills EJ. Anti-tumor necrosis factor (TNF) drugs for the treatment of psoriatic arthritis: an indirect comparison meta-analysis. *Biologics : targets & therapy* 2012; 6: 417-27.

337. Tomlinson CL, Patel S, Meek C, et al. Physiotherapy intervention in Parkinson's disease: systematic review and meta-analysis. *BMJ (Clinical research ed)* 2012; 345: e5004.

338. Tu YK, Needleman I, Chambrone L, Lu HK, Faggion CM, Jr. A Bayesian network meta-analysis on comparisons of enamel matrix derivatives, guided tissue regeneration and their combination therapies. *J Clin Periodontol* 2012; 39(3): 303-14.

339. van der Mark LB, Lyklema PH, Geskus RB, et al. A systematic review with attempted network meta-analysis of asthma therapy recommended for five to eighteen year olds in GINA steps three and four. *BMC Pulm Med* 2012; 12: 63.

340. Vejakama P, Thakkinstian A, Lertrattananon D, Ingsathit A, Ngarmukos C, Attia J. Reno-protective effects of renin-angiotensin system blockade in type 2 diabetic patients: a systematic review and network meta-analysis. *Diabetologia* 2012; 55(3): 566-78.

341. Wang SY, Chu H, Shamliyan T, et al. Network meta-analysis of margin threshold for women with ductal carcinoma in situ. *J Natl Cancer Inst* 2012; 104(7): 507-16.

342. Whegang Youdom S, Samson A, Basco LK, Thalabard JC. Multiple treatment comparisons in a series of anti-malarial trials with an ordinal primary outcome and repeated treatment evaluations. *Malaria journal* 2012; 11: 147.

343. Wolff RF, Aune D, Truyers C, et al. Systematic review of efficacy and safety of buprenorphine versus fentanyl or morphine in patients with chronic moderate to severe pain. *Curr Med Res Opin* 2012; 28(5): 833-45.

344. Woods B, Paracha N, Scott DA, Thatcher N. Raltitrexed plus cisplatin is cost-effective compared with pemetrexed plus cisplatin in patients with malignant pleural mesothelioma. *Lung Cancer* 2012; 75(2): 261-7.

345. Youn JH, Lord J, Hemming K, Girling A, Buxton M. Bayesian meta-analysis on medical devices: application to implantable cardioverter defibrillators. *International journal of technology assessment in health care* 2012; 28(2): 115-24.

346. Zagmutt FJ, Tarrants ML. Indirect Comparisons of Adverse Events and Dropout Rates in Early Parkinson's Disease Trials of Pramipexole, Ropinirole, and Rasagiline. *International Journal of Neuroscience* 2012; 122(7): 345-53.

347. Zintzaras E, Doxani C, Mprotsis T, Schmid CH, Hadjigeorgiou GM. Network analysis of randomized controlled trials in multiple sclerosis. *Clin Ther* 2012; 34(4): 857-69 e9.

348. Akbar A, Abu Dayyeh BK, Baron TH, Wang Z, Altayar O, Murad MH. Rectal nonsteroidal anti-inflammatory drugs are superior to pancreatic duct stents in preventing pancreatitis after endoscopic retrograde cholangiopancreatography: a network meta-analysis. *Clinical gastroenterology and hepatology : the official clinical practice journal of the American Gastroenterological Association* 2013; 11(7): 778-83.

349. Akshintala VS, Hutfless SM, Colantuoni E, et al. Systematic review with network meta-analysis: pharmacological prophylaxis against post-ERCP pancreatitis. *Aliment Pharmacol Ther* 2013; 38(11-12): 1325-37.

350. Alba E, Ciruelos E, Lopez R, et al. Cost-utility analysis of nanoparticle albumin-bound paclitaxel versus paclitaxel in monotherapy in pretreated metastatic breast cancer in Spain. *Expert Review of Pharmacoeconomics & Outcomes Research* 2013; 13(3): 381-+.

351. Assiri A, Al-Majzoub O, Kanaan AO, Donovan JL, Silva M. Mixed treatment comparison meta-analysis of aspirin, warfarin, and new anticoagulants for stroke prevention in patients with nonvalvular atrial fibrillation. *Clin Ther* 2013; 35(7): 967-84 e2.

352. Bakalos G, Miligkos M, Doxani C, Mpoulimari I, Rodopoulou P, Zintzaras E. Assessing the relative effectiveness and tolerability of treatments in small cell lung cancer: a network meta-analysis. *Cancer epidemiology* 2013; 37(5): 675-82.

353. Bangalore S, Amoroso N, Fusaro M, Kumar S, Feit F. Outcomes with various drug-eluting or bare metal stents in patients with ST-segment-elevation myocardial infarction: a mixed treatment comparison analysis of trial level data from 34 068 patient-years of follow-up from randomized trials. *Circulation Cardiovascular interventions* 2013; 6(4): 378-90.

354. Bangalore S, Toklu B, Amoroso N, et al. Bare metal stents, durable polymer drug eluting stents, and biodegradable polymer drug eluting stents for coronary artery disease: mixed treatment comparison meta-analysis. *BMJ (Clinical research ed)* 2013; 347: f6625.

355. Bansal M, Farrugia A, Balboni S, Martin G. Relative survival benefit and morbidity with fluids in severe sepsis - a network meta-analysis of alternative therapies. *Current drug safety* 2013; 8(4): 236-45.

356. Barth J, Munder T, Gerger H, et al. Comparative efficacy of seven psychotherapeutic interventions for patients with depression: a network meta-analysis. *PLoS medicine* 2013; 10(5): e1001454.

357. Bicket MC, Gupta A, Brown CHt, Cohen SP. Epidural injections for spinal pain: a systematic review and meta-analysis evaluating the "control" injections in randomized controlled trials. *Anesthesiology* 2013; 119(4): 907-31.

358. Biondi-Zoccai G, Malavasi V, D'Ascenzo F, et al. Comparative effectiveness of novel oral anticoagulants for atrial fibrillation: evidence from pair-wise and warfarin-controlled network meta-analyses. *HSR proceedings in intensive care & cardiovascular anesthesia* 2013; 5(1): 40-54.

359. Bittl JA, He Y, Jacobs AK, Yancy CW, Normand SL. Bayesian methods affirm the use of percutaneous coronary intervention to improve survival in patients with unprotected left main coronary artery disease. *Circulation* 2013; 127(22): 2177-85.

360. Bodalia PN, Grosso AM, Sofat R, et al. Comparative efficacy and tolerability of anti-epileptic drugs for refractory focal epilepsy: systematic review and network meta-analysis reveals the need for long term comparator trials. *Br J Clin Pharmacol* 2013; 76(5): 649-67.

361. Braun SR, Gregor B, Tran US. Comparing bona fide psychotherapies of depression in adults with two meta-analytical approaches. *Plos One* 2013; 8(6): e68135.

362. Brigo F, Igwe SC, Nardone R, Tezzon F, Bongiovanni LG, Trinka E. A common reference-based indirect comparison meta-analysis of intravenous valproate versus intravenous phenobarbitone for convulsive status epilepticus. *Epileptic disorders : international epilepsy journal with videotape* 2013; 15(3): 314-23.

363. Brown T, Pilkington G, Bagust A, et al. Clinical effectiveness and cost-effectiveness of first-line chemotherapy for adult patients with locally advanced or metastatic non-small cell lung cancer: a systematic review and economic evaluation. *Health technology assessment (Winchester, England)* 2013; 17(31): 1-278.

364. Buti J, Baccini M, Nieri M, La Marca M, Pini-Prato GP. Bayesian network meta-analysis of root coverage procedures: ranking efficacy and identification of best treatment. *J Clin Periodontol* 2013; 40(4): 372-86.

365. Cahill K, Stevens S, Perera R, Lancaster T. Pharmacological interventions for smoking cessation: an overview and network meta-analysis. *The Cochrane database of systematic reviews* 2013; 5: CD009329.

366. Castellucci LA, Cameron C, Le Gal G, et al. Efficacy and safety outcomes of oral anticoagulants and antiplatelet drugs in the secondary prevention of venous thromboembolism: systematic review and network meta-analysis. *BMJ (Clinical research ed)* 2013; 347: f5133.

367. Cawston H, Davie A, Paget MA, Skljarevski V, Happich M. Efficacy of duloxetine versus alternative oral therapies: An indirect comparison of randomised clinical trials in chronic low back pain. *European Spine Journal* 2013; 22(9): 1996-2009.

368. Chatterjee S, Biondi-Zoccai G, Abbate A, et al. Benefits of beta blockers in patients with heart failure and reduced ejection fraction: network meta-analysis. *BMJ (Clinical research ed)* 2013; 346: f55.

369. Chatterjee S, Ghose A, Sharma A, Guha G, Mukherjee D, Frankel R. Comparing newer oral anti-platelets prasugrel and ticagrelor in reduction of ischemic events-evidence from a network meta-analysis. *Journal of thrombosis and thrombolysis* 2013; 36(3): 223-32.

370. Chatterjee S, Sardar P, Biondi-Zoccai G, Kumbhani DJ. New Oral Anticoagulants and the Risk of Intracranial Hemorrhage: Traditional and Bayesian Meta-analysis and Mixed Treatment Comparison of Randomized Trials of New Oral Anticoagulants in Atrial Fibrillation. *JAMA neurology* 2013.

371. Chatterjee S, Sardar P, Mukherjee D, Lichstein E, Aikat S. Timing and route of amiodarone for prevention of postoperative atrial fibrillation after cardiac surgery: a network regression meta-analysis. *Pacing and clinical electrophysiology : PACE* 2013; 36(8): 1017-23.

372. Conde-Agudelo A, Romero R, Nicolaides K, et al. Vaginal progesterone vs. cervical cerclage for the prevention of preterm birth in women with a sonographic short cervix, previous preterm birth, and singleton gestation: a systematic review and indirect comparison metaanalysis. *American journal of obstetrics and gynecology* 2013; 208(1): 42 e1- e18.

373. Contin P, Goossen K, Grummich K, et al. ENERgized vessel sealing systems versus CONventional hemostasis techniques in thyroid surgery--the ENERCON systematic review and network meta-analysis. *Langenbeck's archives of surgery / Deutsche Gesellschaft fur Chirurgie* 2013; 398(8): 1039-56.

374. Cooper C, Lester R, Thorlund K, et al. Direct-acting antiviral therapies for hepatitis C genotype 1 infection: a multiple treatment comparison meta-analysis. *QJM* 2013; 106(2): 153-63.

375. Cope S, Donohue JF, Jansen JP, et al. Comparative efficacy of long-acting bronchodilators for COPD--a network meta-analysis. *Respir Res* 2013; 14: 100.

376. Cope S, Ouwens MJ, Jansen JP, Schmid P. Progression-free survival with fulvestrant 500 mg and alternative endocrine therapies as second-line treatment for advanced breast cancer: a network meta-analysis with parametric survival models. *Value in health : the journal of the International Society for Pharmacoeconomics and Outcomes Research* 2013; 16(2): 403-17.

377. Corbett MS, Rice SJ, Madurasinghe V, et al. Acupuncture and other physical treatments for the relief of pain due to osteoarthritis of the knee: network meta-analysis. *Osteoarthritis and cartilage / OARS, Osteoarthritis Research Society* 2013; 21(9): 1290-8.

378. Coyle D, Coyle K, Cameron C, et al. Cost-effectiveness of new oral anticoagulants compared with warfarin in preventing stroke and other cardiovascular events in patients with atrial fibrillation. *Value in health : the journal of the International Society for Pharmacoeconomics and Outcomes Research* 2013; 16(4): 498-506.

379. Das R, Cope S, Ouwens M, Turner P, Howlett M. Economic evaluation of fulvestrant 500 mg versus generic nonsteroidal aromatase inhibitors in patients with advanced breast cancer in the United Kingdom. *Clin Ther* 2013; 35(3): 246-60 e5.

380. Datto C, Hellmund R, Siddiqui MK. Efficacy and tolerability of naproxen/esomeprazole magnesium tablets compared with non-specific NSAIDs and COX-2 inhibitors: A systematic review and network analyses. *Open Access Rheumatology: Research and Reviews* 2013; 5.

381. Dogliotti A, Paolasso E, Giugliano RP. Current and new oral antithrombotics in non-valvular atrial fibrillation: a network meta-analysis of 79 808 patients. *Heart* 2013.

382. Dong YH, Lin HH, Shau WY, Wu YC, Chang CH, Lai MS. Comparative safety of inhaled medications in patients with chronic obstructive pulmonary disease: systematic review and mixed treatment comparison meta-analysis of randomised controlled trials. *Thorax* 2013; 68(1): 48-56.

383. Dranitsaris G, Schmitz S, Broom RJ. Small molecule targeted therapies for the second-line treatment for metastatic renal cell carcinoma: a systematic review and indirect comparison of safety and efficacy. *Journal of cancer research and clinical oncology* 2013; 139(11): 1917-26.

384. Dretzke J, Meadows A, Novielli N, Huissoon A, Fry-Smith A, Meads C. Subcutaneous and sublingual immunotherapy for seasonal allergic rhinitis: a systematic review and indirect comparison. *The Journal of allergy and clinical immunology* 2013; 131(5): 1361-6.

385. Faggion CM, Jr., Chambrone L, Listl S, Tu YK. Network meta-analysis for evaluating interventions in implant dentistry: the case of peri-implantitis treatment. *Clinical implant dentistry and related research* 2013; 15(4): 576-88.

386. Fang Y, Ding Y, Guo Q, Xing J, Long Y, Zong Z. Radioiodine therapy for patients with differentiated thyroid cancer after thyroidectomy: Direct comparison and network meta-analyses. *J Endocrinol Invest* 2013; 36(10): 896-902.

387. Filippini G, Del Giovane C, Vacchi L, et al. Immunomodulators and immunosuppressants for multiple sclerosis: a network meta-analysis. *The Cochrane database of systematic reviews* 2013; 6: CD008933.

388. Ford JA, Jones R, Elders A, et al. Denosumab for treatment of bone metastases secondary to solid tumours: systematic review and network meta-analysis. *European journal of cancer (Oxford, England : 1990)* 2013; 49(2): 416-30.

389. Freemantle N, Cooper C, Diez-Perez A, et al. Results of indirect and mixed treatment comparison of fracture efficacy for osteoporosis treatments: a meta-analysis. *Osteoporosis international : a journal established as result of cooperation between the European Foundation for Osteoporosis and the National Osteoporosis Foundation of the USA* 2013; 24(1): 209-17.

390. Fusaro M, Cassese S, Ndrepepa G, et al. Paclitaxel-coated balloon or primary bare nitinol stent for revascularization of femoropopliteal artery: a meta-analysis of randomized trials versus uncoated balloon and an adjusted indirect comparison. *Int J Cardiol* 2013; 168(4): 4002-9.

391. Gagne JJ, Bykov K, Choudhry NK, Toomey TJ, Connolly JG, Avorn J. Effect of smoking on comparative efficacy of antiplatelet agents: systematic review, meta-analysis, and indirect comparison. *BMJ (Clinical research ed)* 2013; 347: f5307.

392. Galvan-Banqueri M, Marin Gil R, Santos Ramos B, Bautista Paloma FJ. Biological treatments for moderate-to-severe psoriasis: indirect comparison. *J Clin Pharm Ther* 2013; 38(2): 121-30.

393. Gao L, Xia L, Zhao FL, Li SC. Clinical efficacy and safety of the newer antiepileptic drugs as adjunctive treatment in adults with refractory partial-onset epilepsy: a meta-analysis of randomized placebo-controlled trials. *Epilepsy research* 2013; 103(1): 31-44.

394. Garas G, Okabayashi K, Ashrafian H, et al. Which hemostatic device in thyroid surgery? A network meta-analysis of surgical technologies. *Thyroid : official journal of the American Thyroid Association* 2013; 23(9): 1138-50.

395. Goralczyk AD, Cameron S, Amanzada A. Treatment of chronic HCV genotype 1 infection with telaprevir: a Bayesian mixed treatment comparison of fixed-length and response-guided treatment regimens in treatment-naive and -experienced patients. *BMC gastroenterology* 2013; 13: 148.

396. Gross JL, Rogers J, Polhamus D, et al. A novel model-based meta-analysis to indirectly estimate the comparative efficacy of two medications: an example using DPP-4 inhibitors, sitagliptin and linagliptin, in treatment of type 2 diabetes mellitus. *Bmj Open* 2013; 3(3).

397. Gupta AK, Paquet M. Network meta-analysis of the outcome 'participant complete clearance' in nonimmunosuppressed participants of eight interventions for actinic keratosis: a follow-up on a Cochrane review. *The British journal of dermatology* 2013; 169(2): 250-9.

398. Hadjigeorgiou GM, Doxani C, Miligkos M, et al. A network meta-analysis of randomized controlled trials for comparing the effectiveness and safety profile of treatments with marketing authorization for relapsing multiple sclerosis. *J Clin Pharm Ther* 2013.

399. Hoaglin DC, Filonenko A, Glickman ME, Wasiak R, Gidwani R. Use of mixed-treatment-comparison methods in estimating efficacy of treatments for heavy menstrual bleeding. *Eur J Med Res* 2013; 18: 17.

400. Hochberg MC, Berry S, Broglio K, et al. Mixed treatment comparison of efficacy and tolerability of biologic agents in patients with rheumatoid arthritis. *Curr Med Res Opin* 2013; 29(10): 1213-22.

401. Holme O, Bretthauer M, Fretheim A, Odgaard-Jensen J, Hoff G. Flexible sigmoidoscopy versus faecal occult blood testing for colorectal cancer screening in asymptomatic individuals. *The Cochrane database of systematic reviews* 2013; 9: CD009259.

402. Howell N, Senanayake E, Freemantle N, Pagano D. Putting the record straight on aprotinin as safe and effective: results from a mixed treatment meta-analysis of trials of aprotinin. *The Journal of thoracic and cardiovascular surgery* 2013; 145(1): 234-40.

403. Igarashi A, Kuwabara H, Fahrbach K, Schenkel B. Cost-efficacy comparison of biological therapies for patients with moderate to severe psoriasis in Japan. *The Journal of dermatological treatment* 2013; 24(5): 351-5.

404. Jandhyala R, Fullarton JR, Bennett MI. Efficacy of rapid-onset oral fentanyl formulations vs. oral morphine for cancer-related breakthrough pain: a meta-analysis of comparative trials. *J Pain Symptom Manage* 2013; 46(4): 573-80.

405. Jonas DE, Cusack K, Forneris CA, et al. Psychological and Pharmacological Treatments for Adults With Posttraumatic Stress Disorder (PTSD). Rockville MD; 2013.

406. Jones WS, Schmit KM, Vemulapalli S, et al. Treatment Strategies for Patients With Peripheral Artery Disease. Rockville MD; 2013.

407. Karabis A, Lindner L, Mocarski M, Huisman E, Greening A. Comparative efficacy of aclidinium versus glycopyrronium and tiotropium, as maintenance treatment of moderate to severe COPD patients: a systematic review and network meta-analysis. *Int J Chron Obstruct Pulmon Dis* 2013; 8: 405-23.

408. Khan N, Shah D, Tongbram V, Verdian L, Hawkins N. The efficacy and tolerability of perampanel and other recently approved anti-epileptic drugs for the treatment of refractory partial onset seizure: a systematic review and Bayesian network meta-analysis. *Curr Med Res Opin* 2013; 29(8): 1001-13.

409. Kieran J, Schmitz S, O'Leary A, et al. The relative efficacy of boceprevir and telaprevir in the treatment of hepatitis C virus genotype 1. *Clinical infectious diseases : an official publication of the Infectious Diseases Society of America* 2013; 56(2): 228-35.

410. Kinnaird T, Medic G, Casella G, et al. Relative efficacy of bivalirudin versus heparin monotherapy in patients with ST-segment elevation myocardial infarction treated with primary percutaneous coronary intervention: a network meta-analysis. *Journal of blood medicine* 2013; 4: 129-40.

411. Krogh TP, Bartels EM, Ellingsen T, et al. Comparative effectiveness of injection therapies in lateral epicondylitis: a systematic review and network meta-analysis of randomized controlled trials. *The American journal of sports medicine* 2013; 41(6): 1435-46.

412. Kwak HJ, Kim JY, Kim YB, Min SK, Moon BK. Pharmacological prevention of rocuronium-induced injection pain or withdrawal movements: a meta-analysis. *Journal of anesthesia* 2013; 27(5): 742-9.

413. Kwok CS, Pradhan S, Yeong JK, Loke YK. Relative effects of two different enoxaparin regimens as comparators against newer oral anticoagulants: meta-analysis and adjusted indirect comparison. *Chest* 2013; 144(2): 593-600.

414. Landoni G, Greco T, Biondi-Zoccai G, et al. Anaesthetic drugs and survival: a Bayesian network meta-analysis of randomized trials in cardiac surgery. *Br J Anaesth* 2013; 111(6): 886-96.

415. Larkin J, Paine A, Tumur I, et al. Second-line treatments for the management of advanced renal cell carcinoma: systematic review and meta-analysis. *Expert opinion on pharmacotherapy* 2013; 14(1): 27-39.

416. Lee VW, Schwander B, Lee VH. Effectiveness and cost-effectiveness of erlotinib versus gefitinib in first-line treatment of epidermal growth factor receptor-activating mutation-positive non-small-cell lung cancer patients in Hong Kong. *Hong Kong medical journal = Xianggang yi xue za zhi / Hong Kong Academy of Medicine* 2013.

417. Lema Zuluaga GL, Serna Agudelo RE, Zuleta Tobon JJ. Preservation solutions for liver transplantation in adults: celsior versus custodiol: a systematic review and meta-analysis with an indirect comparison of randomized trials. *Transplantation proceedings* 2013; 45(1): 25-32.

418. Leucht S, Cipriani A, Spineli L, et al. Comparative efficacy and tolerability of 15 antipsychotic drugs in schizophrenia: a multiple-treatments meta-analysis. *Lancet* 2013; 382(9896): 951-62.

419. Levi Marpillat N, Macquin-Mavier I, Tropeano AI, Bachoud-Levi AC, Maison P. Antihypertensive classes, cognitive decline and incidence of dementia: a network meta-analysis. *Journal of hypertension* 2013; 31(6): 1073-82.

420. Liao WC, Chien KL, Lin YL, et al. Adjuvant treatments for resected pancreatic adenocarcinoma: a systematic review and network meta-analysis. *The lancet oncology* 2013; 14(11): 1095-103.

421. Lin PY, Cheng YW, Chu CY, Chien KL, Lin CP, Tu YK. In-office treatment for dentin hypersensitivity: a systematic review and network meta-analysis. *J Clin Periodontol* 2013; 40(1): 53-64.

422. Liu J, Dong J, Wang L, Su Y, Yan P, Sun S. Comparative efficacy and acceptability of antidepressants in Parkinson's disease: a network meta-analysis. *Plos One* 2013; 8(10): e76651.

423. Maggard-Gibbons M, Maglione M, Livhits M, et al. Bariatric surgery for weight loss and glycemic control in nonmorbidly obese adults with diabetes: a systematic review. *Jama* 2013; 309(21): 2250-61.

424. Malloy RJ, Kanaan AO, Silva MA, Donovan JL. Evaluation of antiplatelet agents for secondary prevention of stroke using mixed treatment comparison meta-analysis. *Clin Ther* 2013; 35(10): 1490-500 e7.

425. Mavranezouli I, Meader N, Cape J, Kendall T. The cost effectiveness of pharmacological treatments for generalized anxiety disorder. *Pharmacoeconomics* 2013; 31(4): 317-33.

426. Mealing S, Barcena L, Hawkins N, et al. The relative efficacy of imatinib, dasatinib and nilotinib for newly diagnosed chronic myeloid leukemia: a systematic review and network meta-analysis. *Experimental hematology & oncology* 2013; 2(1): 5.

427. Meissner K, Fassler M, Rucker G, et al. Differential effectiveness of placebo treatments: a systematic review of migraine prophylaxis. *JAMA internal medicine* 2013; 173(21): 1941-51.

428. Migliore A, Broccoli S, Massafra U, Cassol M, Frediani B. Ranking antireabsorptive agents to prevent vertebral fractures in postmenopausal osteoporosis by mixed treatment comparison meta-analysis. *European review for medical and pharmacological sciences* 2013; 17(5): 658-67.

429. Mitchell SA, Simon TA, Raza S, et al. The efficacy and safety of oral anticoagulants in warfarin-suitable patients with nonvalvular atrial fibrillation: systematic review and meta-analysis. *Clinical and applied thrombosis/hemostasis : official journal of the International Academy of Clinical and Applied Thrombosis/Hemostasis* 2013; 19(6): 619-31.

430. Naci H, Brugts J, Ades T. Comparative tolerability and harms of individual statins: a study-level network meta-analysis of 246 955 participants from 135 randomized, controlled trials. *Circulation Cardiovascular quality and outcomes* 2013; 6(4): 390-9.

431. Naci H, Brugts JJ, Fleurence R, Ades AE. Dose-comparative effects of different statins on serum lipid levels: a network meta-analysis of 256,827 individuals in 181 randomized controlled trials. *European journal of preventive cardiology* 2013; 20(4): 658-70.

432. Naci H, Brugts JJ, Fleurence R, Ades AE. Comparative effects of statins on major cerebrovascular events: a multiple-treatments meta-analysis of placebo-controlled and active-comparator trials. *QJM* 2013; 106(4): 299-306.

433. Naci H, Brugts JJ, Fleurence R, Tsoi B, Toor H, Ades AE. Comparative benefits of statins in the primary and secondary prevention of major coronary events and all-cause mortality: a network meta-analysis of placebo-controlled and active-comparator trials. *European journal of preventive cardiology* 2013; 20(4): 641-57.

434. Naci H, Ioannidis JPA. Comparative effectiveness of exercise and drug interventions on mortality outcomes: metaepidemiological study. *BMJ (Clinical research ed)* 2013; 347: f5577.

435. Naudet F, Millet B, Charlier P, Reymann JM, Maria AS, Falissard B. Which placebo to cure depression? A thought-provoking network meta-analysis. *BMC Med* 2013; 11: 230.

436. Navarese EP, Buffon A, Andreotti F, et al. Meta-analysis of impact of different types and doses of statins on new-onset diabetes mellitus. *The American journal of cardiology* 2013; 111(8): 1123-30.

437. Navarese EP, Tandjung K, Claessen B, et al. Safety and efficacy outcomes of first and second generation durable polymer drug eluting stents and biodegradable polymer biolimus eluting stents in clinical practice: comprehensive network meta-analysis. *BMJ (Clinical research ed)* 2013; 347: f6530.

438. Ney JP, Devine EB, Watanabe JH, Sullivan SD. Comparative efficacy of oral pharmaceuticals for the treatment of chronic peripheral neuropathic pain: meta-analysis and indirect treatment comparisons. *Pain medicine (Malden, Mass)* 2013; 14(5): 706-19.

439. Nuesch E, Hauser W, Bernardy K, Barth J, Juni P. Comparative efficacy of pharmacological and non-pharmacological interventions in fibromyalgia syndrome: network meta-analysis. *Ann Rheum Dis* 2013; 72(6): 955-62.

440. O'Connor AM, Coetzee JF, da Silva N, Wang C. A mixed treatment comparison meta-analysis of antibiotic treatments for bovine respiratory disease. *Prev Vet Med* 2013; 110(2): 77-87.

441. Ollendorf DA, Colby JA, Pearson SD. Comparative effectiveness of anti-VEGF agents for diabetic macular edema. *International journal of technology assessment in health care* 2013; 29(4): 392-401.

442. Otten MH, Anink J, Spronk S, van Suijlekom-Smit LW. Efficacy of biological agents in juvenile idiopathic arthritis: a systematic review using indirect comparisons. *Ann Rheum Dis* 2013; 72(11): 1806-12.

443. Palmerini T, Biondi-Zoccai G, Della Riva D, et al. Clinical outcomes with drug-eluting and bare-metal stents in patients with ST-segment elevation myocardial infarction: evidence from a comprehensive network meta-analysis. *J Am Coll Cardiol* 2013; 62(6): 496-504.

444. Palmerini T, Biondi-Zoccai G, Riva DD, et al. Risk of stroke with percutaneous coronary intervention compared with on-pump and off-pump coronary artery bypass graft surgery: Evidence from a comprehensive network meta-analysis. *American heart journal* 2013; 165(6): 910-7 e14.

445. Palmieri C, Fullarton JR, Brown J. Comparative efficacy of bisphosphonates in metastatic breast and prostate cancer and multiple myeloma: a mixed-treatment meta-analysis. *Clinical cancer research : an official journal of the American Association for Cancer Research* 2013; 19(24): 6863-72.

446. Pandor A, Gomersall T, Stevens JW, et al. Remote monitoring after recent hospital discharge in patients with heart failure: a systematic review and network meta-analysis. *Heart* 2013; 99(23): 1717-26.

447. Pandor A, Thokala P, Gomersall T, et al. Home telemonitoring or structured telephone support programmes after recent discharge in patients with heart failure: systematic review and economic evaluation. *Health technology assessment (Winchester, England)* 2013; 17(32): 1-207, v-vi.

448. Pink J, Pirmohamed M, Hughes DA. Comparative Effectiveness of Dabigatran, Rivaroxaban, Apixaban, and Warfarin in the Management of Patients With Nonvalvular Atrial Fibrillation. *Clinical Pharmacology & Therapeutics* 2013; 94(2): 269-76.

449. Rashiq S, Vandermeer B, Abou-Setta AM, Beaupre LA, Jones CA, Dryden DM. Efficacy of supplemental peripheral nerve blockade for hip fracture surgery: multiple treatment comparison. *Canadian journal of anaesthesia = Journal canadien d'anesthesie* 2013; 60(3): 230-43.

450. Ribeiro RA, Ziegelmann PK, Duncan BB, et al. Impact of statin dose on major cardiovascular events: a mixed treatment comparison meta-analysis involving more than 175,000 patients. *Int J Cardiol* 2013; 166(2): 431-9.

451. Robertson C, Close A, Fraser C, et al. Relative effectiveness of robot-assisted and standard laparoscopic prostatectomy as alternatives to open radical prostatectomy for treatment of localised prostate cancer: a systematic review and mixed treatment comparison meta-analysis. *BJU international* 2013; 112(6): 798-812.

452. Rotta I, Ziegelmann PK, Otuki MF, Riveros BS, Bernardo NL, Correr CJ. Efficacy of topical antifungals in the treatment of dermatophytosis: a mixed-treatment comparison meta-analysis involving 14 treatments. *JAMA dermatology* 2013; 149(3): 341-9.

453. Rudroju N, Bansa D, Talakokkula ST, et al. Comparative efficacy and safety of six antidepressants and anticonvulsants in painful diabetic neuropathy: a network meta-analysis. *Pain physician* 2013; 16(6): E705-14.

454. Samarasekera EJ, Sawyer L, Wonderling D, Tucker R, Smith CH. Topical therapies for the treatment of plaque psoriasis: systematic review and network meta-analyses. *The British journal of dermatology* 2013; 168(5): 954-67.

455. Sanches ACC, Correr CJ, Venson R, et al. Insulin analogues Versus Human Insulin in Type 1 diabetes: Direct and indirect meta-analyses of efficacy and safety. *Brazilian Journal of Pharmaceutical Sciences* 2013; 49(3): 501-9.

456. Sardar P, Chatterjee S, Wu WC, et al. New oral anticoagulants are not superior to warfarin in secondary prevention of stroke or transient ischemic attacks, but lower the risk of intracranial bleeding: insights from a meta-analysis and indirect treatment comparisons. *Plos One* 2013; 8(10): e77694.

457. Schoenberg MB, Marx S, Kersten JF, et al. Laparoscopic Heller myotomy versus endoscopic balloon dilatation for the treatment of achalasia: a network meta-analysis. *Ann Surg* 2013; 258(6): 943-52.

458. Schwingshackl L, Dias S, Strasser B, Hoffmann G. Impact of different training modalities on anthropometric and metabolic characteristics in overweight/obese subjects: a systematic review and network meta-analysis. *Plos One* 2013; 8(12): e82853.

459. Scott DA, Boye KS, Timlin L, Clark JF, Best JH. A network meta-analysis to compare glycaemic control in patients with type 2 diabetes treated with exenatide once weekly or liraglutide once daily in comparison with insulin glargine, exenatide twice daily or placebo. *Diabetes, obesity & metabolism* 2013; 15(3): 213-23.

460. Shamliyan TA, Choi JY, Ramakrishnan R, et al. Preventive pharmacologic treatments for episodic migraine in adults. *J Gen Intern Med* 2013; 28(9): 1225-37.

461. Shi KQ, Liu WY, Pan ZZ, et al. Secondary prophylaxis of variceal bleeding for cirrhotic patients: a multiple-treatments meta-analysis. *European journal of clinical investigation* 2013.

462. SJ E, V H, L N, N T. Lithium or an atypical antipsychotic drug in the management of treatment-resistant depression: a systematic review and economic evaluation. *Health technology assessment (Winchester, England)* 2013; 17(54): 1-190.

463. Skoetz N, Trelle S, Rancea M, et al. Effect of initial treatment strategy on survival of patients with advanced-stage Hodgkin's lymphoma: a systematic review and network meta-analysis. *The lancet oncology* 2013; 14(10): 943-52.

464. Snedecor SJ, Sudharshan L, Cappelleri JC, et al. Systematic review and comparison of pharmacologic therapies for neuropathic pain associated with spinal cord injury. *Journal of Pain Research* 2013; 6: 539-47.

465. Stegeman BH, de Bastos M, Rosendaal FR, et al. Different combined oral contraceptives and the risk of venous thrombosis: systematic review and network meta-analysis. *BMJ (Clinical research ed)* 2013; 347: f5298.

466. Sundaresh V, Brito JP, Wang Z, et al. Comparative effectiveness of therapies for Graves' hyperthyroidism: a systematic review and network meta-analysis. *The Journal of clinical endocrinology and metabolism* 2013; 98(9): 3671-7.

467. Terasawa T, Trikalinos NA, Djulbegovic B, Trikalinos TA. Comparative efficacy of first-line therapies for advanced-stage chronic lymphocytic leukemia: a multiple-treatment meta-analysis. *Cancer Treat Rev* 2013; 39(4): 340-9.

468. Tomlinson CL, Patel S, Meek C, et al. Physiotherapy versus placebo or no intervention in Parkinson's disease. *The Cochrane database of systematic reviews* 2013; 9: CD002817.

469. Uthman OA, van der Windt DA, Jordan JL, et al. Exercise for lower limb osteoarthritis: systematic review incorporating trial sequential analysis and network meta-analysis. *BMJ (Clinical research ed)* 2013; 347: f5555.

470. Wiens A, Lenzi L, Venson R, et al. Comparative efficacy of oral nucleoside or nucleotide analog monotherapy used in chronic hepatitis B: a mixed-treatment comparison meta-analysis. *Pharmacotherapy* 2013; 33(2): 144-51.

471. Wu HY, Huang JW, Lin HJ, et al. Comparative effectiveness of renin-angiotensin system blockers and other antihypertensive drugs in patients with diabetes: systematic review and bayesian network meta-analysis. *BMJ (Clinical research ed)* 2013; 347: f6008.

472. Wu MS, Tan SC, Xiong T. Indirect comparison of randomised controlled trials: comparative efficacy of dexlansoprazole vs. esomeprazole in the treatment of gastro-oesophageal reflux disease. *Aliment Pharmacol Ther* 2013; 38(2): 190-201.

473. Yang Q, Wei Y, Chen YX, Zhou SW, Jiang ZM, Xie DR. Indirect comparison showed survival benefit from adjuvant chemoradiotherapy in completely resected gastric cancer with D2 lymphadenectomy. *Gastroenterology Research and Practice* 2013; 2013.

474. Yuan J, Zhang R, Yang Z, et al. Comparative effectiveness and safety of oral phosphodiesterase type 5 inhibitors for erectile dysfunction: a systematic review and network meta-analysis. *Eur Urol* 2013; 63(5): 902-12.

475. Zaccara G, Giovannelli F, Maratea D, Fadda V, Verrotti A. Neurological adverse events of new generation sodium blocker antiepileptic drugs. Meta-analysis of randomized, double-blinded studies with eslicarbazepine acetate, lacosamide and oxcarbazepine. *Seizure : the journal of the British Epilepsy Association* 2013; 22(7): 528-36.

476. Zou Y, Sheng Z, Niu S, Wang H, Yu J, Xu J. Lenalidomide versus thalidomide based regimens as first-line therapy for patients with multiple myeloma. *Leukemia & lymphoma* 2013; 54(10): 2219-25.

477. Afghani E, Akshintala VS, Khashab MA, et al. 5-Fr vs. 3-Fr pancreatic stents for the prevention of post-ERCP pancreatitis in high-risk patients: a systematic review and network meta-analysis. *Endoscopy* 2014; 46(7): 573-9.

478. Agapova M, Devine EB, Nguyen H, Wolf FM, Inoue LYT. Using indirect comparisons to compare interventions within a Cochrane review: A tool for comparative effectiveness research. *Journal of Comparative Effectiveness Research* 2014; 3(4): 345-57.

479. Alotaibi G, Alsaleh K, Wu C, McMurtry MS. Dabigatran, rivaroxaban and apixaban for extended venous thromboembolism treatment: network meta-analysis. *International Angiology* 2014; 33(4): 301-8.

480. Ashby RL, Gabe R, Ali S, et al. VenUS IV (Venous leg Ulcer Study IV) null Compression hosiery compared with compression bandaging in the treatment of venous leg ulcers: A randomised controlled trial, mixed-treatment comparison and decision-analytic model. *Health Technology Assessment* 2014; 18(57): i-xxvii+1-293.

481. Bachelot T, McCool R, Duffy S, et al. Comparative efficacy of everolimus plus exemestane versus fulvestrant for hormone-receptor-positive advanced breast cancer following progression/recurrence after endocrine therapy: a network meta-analysis. *Breast Cancer Research and Treatment* 2014; 143(1): 125-33.

482. Bangalore S, Toklu B, Feit F. Outcomes With Coronary Artery Bypass Graft Surgery Versus Percutaneous Coronary Intervention for Patients With Diabetes Mellitus Can Newer Generation Drug-Eluting Stents Bridge the Gap? *Circulation-Cardiovascular Interventions* 2014; 7(4): 518-+.

483. Bangalore S, Toklu B, Kotwal A, et al. Anticoagulant therapy during primary percutaneous coronary intervention for acute myocardial infarction: a meta-analysis of randomized trials in the era of stents and P2Y(12) inhibitors. *Bmj-British Medical Journal* 2014; 349.

484. Bateman ED, Esser D, Chirila C, et al. Magnitude of effect of asthma treatments on Asthma Quality of Life Questionnaire and Asthma Control Questionnaire scores: Systematic review and network meta-analysis. *Journal of Allergy and Clinical Immunology* 2014.

485. Biondi-Zoccai G, Lotrionte M, Thomsen HS, et al. Nephropathy after administration of iso-osmolar and low-osmolar contrast media: Evidence from a network meta-analysis. *International Journal of Cardiology* 2014; 172(2): 375-80.

486. Biondi-Zoccai G, Peruzzi M, Abbate A, et al. Network meta-analysis on the comparative effectiveness and safety of transcatheter aortic valve implantation with CoreValve or Sapien devices versus surgical replacement. *Database of Abstracts of Reviews of Effects*, 2014. http://onlinelibrary.wiley.com/o/cochrane/cldare/articles/DARE-12014071786/frame.html (accessed.

487. Caldeira D, Pinto FJ, Ferreira JJ. Dyspnea and reversibility profile of P2Y12 antagonists: Systematic review of new antiplatelet drugs. *American Journal of Cardiovascular Drugs* 2014; 14(4): 303-11.

488. Cameron C, Coyle D, Richter T, et al. Systematic review and network meta-analysis comparing antithrombotic agents for the prevention of stroke and major bleeding in patients with atrial fibrillation. *Bmj Open* 2014; 4(6).

489. Carroll C, Hummel S, Leaviss J, et al. Systematic review, network meta-analysis and exploratory cost-effectiveness model of randomized trials of minimally invasive techniques versus surgery for varicose veins. *British Journal of Surgery* 2014; 101(9): 1040-52.

490. Carter P, Achana F, Troughton J, Gray LJ, Khunti K, Davies MJ. A Mediterranean diet improves HbA1c but not fasting blood glucose compared to alternative dietary strategies: a network meta-analysis. *Journal of Human Nutrition and Dietetics* 2014; 27(3): 280-97.

491. Caruba T, Katsahian S, Schramm C, et al. Treatment for Stable Coronary Artery Disease: A Network Meta-Analysis of Cost-Effectiveness Studies. *Plos One* 2014; 9(6).

492. Castellucci LA, Cameron C, Le Gal G, et al. Clinical and Safety Outcomes Associated With Treatment of Acute Venous Thromboembolism A Systematic Review and Meta-analysis. *Jama-Journal of the American Medical Association* 2014; 312(11): 1122-35.

493. Cates CJ, Wieland LS, Oleszczuk M, Kew KM. Safety of regular formoterol or salmeterol in adults with asthma: an overview of Cochrane reviews. *Cochrane Database of Systematic Reviews* 2014; (2).

494. Cawson MR, Mitchell SA, Knight C, et al. Systematic review, network meta-analysis and economic evaluation of biological therapy for the management of active psoriatic arthritis. *Bmc Musculoskeletal Disorders* 2014; 15.

495. Chambers JD, Winn A, Zhong Y, Olchanski N, Cangelosi MJ. Potential Role of Network Meta-Analysis in Value-Based Insurance Design. *American Journal of Managed Care* 2014; 20(8): 641-U170.

496. Chan K, Shah K, Lien K, Coyle D, Lam H, Ko YJ. A Bayesian Meta-Analysis of Multiple Treatment Comparisons of Systemic Regimens for Advanced Pancreatic Cancer. *Plos One* 2014; 9(10).

497. Chen Y, Zhang Y, Tang Y, Huang X, Xie Y. Long-term clinical efficacy and safety of adding cilostazol to dual antiplatelet therapy for patients undergoing PCI: a meta-analysis of randomized trials with adjusted indirect comparisons. *Current Medical Research and Opinion*, 2014. http://onlinelibrary.wiley.com/o/cochrane/cldare/articles/DARE-12013058556/frame.html (accessed.

498. Chilton M, Dunkley A, Carter P, Davies MJ, Khunti K, Gray LJ. The effect of antiobesity drugs on waist circumference: a mixed treatment comparison. *Diabetes Obesity & Metabolism* 2014; 16(3): 237-47.

499. Colmenare H, Fernandez C, Escaned J. Impact of technological developments in drug-eluting stents on patient-focused outcomes: A pooled direct and indirect comparison of randomised trials comparing first- And second-generation drug-eluting stents. *EuroIntervention* 2014; 10(8): 942-52.

500. Cornely OA, Nathwani D, Ivanescu C, Odufowora-Sita O, Retsa P, Odeyemi IA. Clinical efficacy of fidaxomicin compared with vancomycin and metronidazole in Clostridium difficile infections: a meta-analysis and indirect treatment comparison. *The Journal of antimicrobial chemotherapy* 2014; 69(11): 2892-900.

501. Craddy P, Palin HJ, Johnson KI. Comparative Effectiveness of Dipeptidylpeptidase-4 Inhibitors in Type 2 Diabetes: A Systematic Review and Mixed Treatment Comparison. *Diabetes Therapy* 2014; 5(1): 1-41.

502. Cucherat M, Stalmans I, Rouland JF. Relative efficacy and safety of preservative-free latanoprost (T2345) for the treatment of open-angle glaucoma and ocular hypertension: an adjusted Indirect comparison meta-analysis of randomized clinical trials. *J Glaucoma* 2014; 23(1): e69-75.

503. Dahal A, Bellows BK, Sonpavde G, et al. Incidence of severe nephrotoxicity with cisplatin based on renal function eligibility criteria: Indirect comparison meta-analysis. *American Journal of Clinical Oncology: Cancer Clinical Trials* 2014.

504. Dai N, Xu D, Zhang J, et al. Different beta-blockers and initiation time in patients undergoing noncardiac surgery: a meta-analysis. *The American journal of the medical sciences* 2014; 347(3): 235-44.

505. Dai XY, Wang HT, Jing Z, Fu P. The effect of a dual combination of noninsulin antidiabetic drugs on lipids: a systematic review and network meta-analysis. *Current Medical Research and Opinion* 2014; 30(9): 1777-86.

506. Dal Molin A, Allara E, Montani D, et al. Flushing the central venous catheter: is heparin necessary? *Journal of Vascular Access* 2014; 15(4): 241-8.

507. Danese S, Fiorino G, Peyrin-Biroulet L, et al. Biological Agents for Moderately to Severely Active Ulcerative Colitis A Systematic Review and Network Meta-analysis. *Annals of Internal Medicine* 2014; 160(10): 704-+.

508. de Bastos M, Stegeman BH, Rosendaal FR, et al. Combined oral contraceptives: venous thrombosis. *Cochrane Database of Systematic Reviews* 2014; (3).

509. Dequen P, Sutton AJ, Scott DA, Abrams KR. Searching for Indirect Evidence and Extending the Network of Studies for Network Meta-Analysis: Case Study in Venous Thromboembolic Events Prevention Following Elective Total Knee Replacement Surgery. *Value in Health* 2014; 17(4): 416-23.

510. Desamericq G, Schurhoff F, Meary A, et al. Long-term neurocognitive effects of antipsychotics in schizophrenia: a network meta-analysis. *European Journal of Clinical Pharmacology* 2014; 70(2): 127-34.

511. DeSantis SM, Zhu HR. A Bayesian Mixed-Treatment Comparison Meta-analysis of Treatments for Alcohol Dependence and Implications for Planning Future Trials. *Medical Decision Making* 2014; 34(7): 899-910.

512. Dong J, Gao L, Lu W, Xu Z, Zheng J. Pharmacological interventions for acceleration of the onset time of rocuronium: A meta-analysis. *PLoS ONE* 2014; 9(12).

513. Dooley C, Kaur R, Sobieraj DM. Comparison of the efficacy and safety of low molecular weight heparins for venous thromboembolism prophylaxis in medically ill patients. *Current Medical Research and Opinion* 2014; 30(3): 367-80.

514. Dranitsaris G, Ellis AK. Sublingual or subcutaneous immunotherapy for seasonal allergic rhinitis: An indirect analysis of efficacy, safety and cost. *Journal of Evaluation in Clinical Practice* 2014.

515. Dranitsaris G, Kaura S. Lenalidomide versus bortezomib: An indirect comparison. *International Journal of Hematologic Oncology* 2014; 3(2): 131-6.

516. Ebrahim S, Mollon B, Bance S, Busse JW, Bhandari M. Low-intensity pulsed ultrasonography versus electrical stimulation for fracture healing: a systematic review and network meta-analysis. *Canadian Journal of Surgery* 2014; 57(3): E105-E18.

517. Ellis AG, Reginster JY, Luo XM, et al. Indirect comparison of bazedoxifene vs oral bisphosphonates for the prevention of vertebral fractures in postmenopausal osteoporotic women. *Current Medical Research and Opinion* 2014; 30(8): 1617-26.

518. Ellis AG, Reginster JY, Luo XM, et al. Bazedoxifene versus Oral Bisphosphonates for the Prevention of Nonvertebral Fractures in Postmenopausal Women with Osteoporosis at Higher Risk of Fracture: A Network Meta-Analysis. *Value in Health* 2014; 17(4): 424-32.

519. Evans JDW, Morris PJ, Knight SR. Antifungal Prophylaxis in Liver Transplantation: A Systematic Review and Network Meta-Analysis. *American Journal of Transplantation* 2014; 14(12): 2765-76.

520. Faggion CM, Listl S, Fruhauf N, Chang HJ, Tu YK. A systematic review and Bayesian network meta-analysis of randomized clinical trials on non-surgical treatments for peri-implantitis. *Journal of Clinical Periodontology* 2014; 41(10): 1015-25.

521. Florescu DF, Qiu F, Schmidt CM, Kalil AC. A Direct and Indirect Comparison Meta-Analysis on the Efficacy of Cytomegalovirus Preventive Strategies in Solid Organ Transplant. *Clinical Infectious Diseases* 2014; 58(6): 785-803.

522. Ford JA, Shyangdan D, Uthman OA, Lois N, Waugh N. Drug treatment of macular oedema secondary to central retinal vein occlusion: a network meta-analysis. *Bmj Open* 2014; 4(7).

523. Fournier M, Germe M, Theobald K, Scholz GH, Lehmacher W. Indirect comparison of lixisenatide versus neutral protamine Hagedorn insulin as add-on to metformin and sulphonylurea in patients with type 2 diabetes mellitus. *German medical science : GMS e-journal* 2014; 12: Doc14.

524. Freeman KA, Riley A, Duke DC, Fu RW. Systematic Review and Meta-Analysis of Behavioral Interventions for Fecal Incontinence With Constipation. *Journal of Pediatric Psychology* 2014; 39(8): 887-902.

525. Fu WB, Guo HY, Guo JP, et al. Relative efficacy and safety of direct oral anticoagulants in patients with atrial fibrillation by network meta-analysis. *Journal of Cardiovascular Medicine* 2014; 15(12): 873-9.

526. Furukawa TA, Noma H, Caldwell DM, et al. Waiting list may be a nocebo condition in psychotherapy trials: a contribution from network meta-analysis. *Acta Psychiatrica Scandinavica* 2014; 130(3): 181-92.

527. Gerger H, Munder T, Gemperli A, et al. Integrating fragmented evidence by network meta-analysis: relative effectiveness of psychological interventions for adults with post-traumatic stress disorder. *Psychological Medicine* 2014; 44(15): 3151-64.

528. Goodacre S, Stevens JW, Pandor A, et al. Prehospital Noninvasive Ventilation for Acute Respiratory Failure: Systematic Review, Network Meta-analysis, and Individual Patient Data Meta-analysis. *Academic Emergency Medicine* 2014; 21(9): 960-70.

529. Goring SM, Hawkins N, Wygant G, et al. Dapagliflozin compared with other oral anti-diabetes treatments when added to metformin monotherapy: a systematic review and network meta-analysis (Provisional abstract). *Diabetes Obesity and Metabolism*, 2014. http://onlinelibrary.wiley.com/o/cochrane/cldare/articles/DARE-12013067941/frame.html (accessed.

530. Goring SM, Levy AR, Ghement I, et al. A network meta-analysis of the efficacy of belatacept, cyclosporine and tacrolimus for immunosuppression therapy in adult renal transplant recipients. *Current Medical Research and Opinion* 2014; 30(8): 1473-87.

531. Gould AL, Unniachan S, Wu D. Indirect treatment comparison between fixed-dose-combinations of amlodipine/losartan and amlodipine/valsartan in blood pressure control. *International Journal of Clinical Practice* 2014; 68(2): 163-72.

532. Graudal N, Hubeck-Graudal T, Tarp S, Christensen R, Jurgens G. Effect of Combination Therapy on Joint Destruction in Rheumatoid Arthritis: A Network Meta-Analysis of Randomized Controlled Trials. *Plos One* 2014; 9(9).

533. Gresham GK, Wells GA, Gill S, Cameron C, Jonker DJ. Chemotherapy regimens for advanced pancreatic cancer: a systematic review and network meta-analysis. *Bmc Cancer* 2014; 14.

534. Griebeler ML, Morey-Vargas OL, Brito JP, et al. Pharmacologic Interventions for Painful Diabetic Neuropathy An Umbrella Systematic Review and Comparative Effectiveness Network Meta-analysis. *Annals of Internal Medicine* 2014; 161(9): 639-U138.

535. Gupta AK, Charrette A. The efficacy and safety of 5 alpha-reductase inhibitors in androgenetic alopecia: a network meta-analysis and benefit-risk assessment of finasteride and dutasteride. *Journal of Dermatological Treatment* 2014; 25(2): 156-61.

536. Gupta AK, Daigle D, Lyons DCA. Network Meta-analysis of Treatments for Chronic Plaque Psoriasis in Canada. *Journal of Cutaneous Medicine and Surgery* 2014; 18(6): 371-8.

537. Gupta AK, Daigle D, Paquet M. Therapies for onychomycosis: a systematic review and network meta-analysis of mycological cure *Database of Abstracts of Reviews of Effects*, 2014. http://onlinelibrary.wiley.com/o/cochrane/cldare/articles/DARE-12014045322/frame.html (accessed.

538. Hazlewood GS, Metzler C, Tomlinson GA, et al. Non-biologic remission maintenance therapy in adult patients with ANCA-associated vasculitis: A systematic review and network meta-analysis. *Joint Bone Spine* 2014; 81(4): 337-41.

539. Hirschl M, Kundi M. New oral anticoagulants in the treatment of acute venous thromboembolism - a systematic review with indirect comparisons. *VASA Zeitschrift fur Gefasskrankheiten* 2014; 43(5): 353-64.

540. Honyashiki M, Furukawa TA, Noma H, et al. Specificity of CBT for Depression: A Contribution from Multiple Treatments Meta-analyses. *Cognitive Therapy and Research* 2014; 38(3): 249-60.

541. Hutchinson M, Fox RJ, Havrdova E, et al. Efficacy and safety of BG-12 (dimethyl fumarate) and other disease-modifying therapies for the treatment of relapsing-remitting multiple sclerosis: a systematic review and mixed treatment comparison. *Current Medical Research and Opinion* 2014; 30(4): 613-27.

542. Jansen JP, Buckley F, Dejonckheere F, Ogale S. Comparative efficacy of biologics as monotherapy and in combination with methotrexate on patient reported outcomes (PROs) in rheumatoid arthritis patients with an inadequate response to conventional DMARDs - a systematic review and network meta-analysis. *Health and Quality of Life Outcomes* 2014; 12.

543. Johnston BC, Kanters S, Bandayrel K, et al. Comparison of Weight Loss Among Named Diet Programs in Overweight and Obese Adults A Meta-analysis. *Jama-Journal of the American Medical Association* 2014; 312(9): 923-33.

544. Kalil AC, Florescu MC, Grant W, et al. Risk of serious opportunistic infections after solid organ transplantation: interleukin-2 receptor antagonists versus polyclonal antibodies. A meta-analysis. *Expert review of anti-infective therapy* 2014; 12(7): 881-96.

545. Kang N, Sobieraj DM. Indirect treatment comparison of new oral anticoagulants for the treatment of acute venous thromboembolism. *Thrombosis Research* 2014; 133(6): 1145-51.

546. Kang SH, Park KW, Kang DY, et al. Biodegradable-polymer drug-eluting stents vs. bare metal stents vs. durable-polymer drug-eluting stents: a systematic review and Bayesian approach network meta-analysis. *European Heart Journal* 2014; 35(17): 1147-+.

547. Katsanos K, Spiliopoulos S, Karunanithy N, Krokidis M, Sabharwal T, Taylor P. Bayesian network meta-analysis of nitinol stents, covered stents, drug-eluting stents, and drug-coated balloons in the femoropopliteal artery. *Journal of Vascular Surgery* 2014; 59(4): 1123-+.

548. Kew KM, Dias S, Cates CJ. Long-acting inhaled therapy (beta-agonists, anticholinergics and steroids) for COPD: a network meta-analysis. *Cochrane Database of Systematic Reviews* 2014; (3).

549. Kew KM, Seniukovich A. Inhaled steroids and risk of pneumonia for chronic obstructive pulmonary disease. *The Cochrane database of systematic reviews* 2014; 3: CD010115.

550. Kim HL, Lee MY, Park SY, et al. Comparative effectiveness of cycling of tumor necrosis factor-alpha (TNF-alpha) inhibitors versus switching to non-TNF biologics in rheumatoid arthritis patients with inadequate response to TNF-alpha inhibitor using a Bayesian approach. *Archives of Pharmacal Research* 2014; 37(5): 662-70.

551. Kriston L, Wolff A, Westphal A, Holzel LP, Harter M. Efficacy and acceptability of acute treatments for persistent depressive disorder: a network meta-analysis. *Depression and Anxiety*, 2014. http://onlinelibrary.wiley.com/o/cochrane/cldare/articles/DARE-12014007919/frame.html (accessed.

552. Kumagai K, Rouvelas I, Tsai JA, et al. Meta-analysis of postoperative morbidity and perioperative mortality in patients receiving neoadjuvant chemotherapy or chemoradiotherapy for resectable oesophageal and gastro-oesophageal junctional cancers. *The British journal of surgery* 2014; 101(4): 321-38.

553. Laporte S, Chapelle C, Bertoletti L, et al. Indirect comparison meta-analysis of two enoxaparin regimens in patients undergoing major orthopaedic surgery Impact on the interpretation of thromboprophylactic effects of new anticoagulant drugs. *Thrombosis and Haemostasis* 2014; 112(3): 503-10.

554. Le Cleach L, Trinquart L, Do G, et al. Oral antiviral therapy for prevention of genital herpes outbreaks in immunocompetent and nonpregnant patients. *Cochrane Database of Systematic Reviews*, 2014. http://onlinelibrary.wiley.com/doi/10.1002/14651858.CD009036.pub2/abstract (accessed.

555. Leaviss J, Sullivan W, Ren S, et al. What is the clinical effectiveness and cost-effectiveness of cytisine compared with varenicline for smoking cessation? A systematic review and economic evaluation. *Health Technology Assessment* 2014; 18(33): 1-+.

556. Lee SW, Chaiyakunapruk N, Chong HY, Liong ML. Comparative efficacy and safety of various treatment procedures for lower pole renal calculi: a systematic review and network meta-analysis. *Database of Abstracts of Reviews of Effects*, 2014. http://onlinelibrary.wiley.com/o/cochrane/cldare/articles/DARE-12014067803/frame.html (accessed.

557. Leibovici-Weissman Ya, Neuberger A, Bitterman R, Sinclair D, Salam Mohammed A, Paul M. Antimicrobial drugs for treating cholera. *Cochrane Database of Systematic Reviews*, 2014. http://onlinelibrary.wiley.com/doi/10.1002/14651858.CD008625.pub2/abstract (accessed.

558. Leung HWC, Chan ALF, Lin SJ. Indirect comparisons of efficacy and safety between seven newer targeted agents for metastatic renal cell carcinoma: A network meta-analysis of randomised clinical trials. *Molecular and Clinical Oncology* 2014; 2(5): 858-64.

559. Li L, Tian JH, Tian HL, Sun R, Wang Q, Yang KH. The Efficacy and Safety of Different Kinds of Laparoscopic Cholecystectomy: A Network Meta Analysis of 43 Randomized Controlled Trials. *Plos One* 2014; 9(2).

560. Li LT, Hicks SC, Davila JA, et al. Circular closure is associated with the lowest rate of surgical site infection following stoma reversal: a systematic review and multiple treatment meta-analysis. *Colorectal Disease* 2014; 16(6): 406-16.

561. Liang WH, Wu X, Fang WF, et al. Network Meta-Analysis of Erlotinib, Gefitinib, Afatinib and Icotinib in Patients with Advanced Non-Small-Cell Lung Cancer Harboring EGFR Mutations. *Plos One* 2014; 9(2).

562. Lin L, Zhao YJ, Chew PTK, et al. Comparative Efficacy and Tolerability of Topical Prostaglandin Analogues for Primary Open-Angle Glaucoma and Ocular Hypertension. *Annals of Pharmacotherapy* 2014; 48(12): 1585-93.

563. Lin PY, Chen HS, Wang YH, Tu YK. Primary molar pulpotomy: A systematic review and network meta-analysis. *Journal of Dentistry* 2014; 42(9): 1060-77.

564. Liu Y, Yan R, Song AQ, et al. Aliskiren/Amlodipine vs. Aliskiren/Hydrochlorothiazide in Hypertension: Indirect Meta-Analysis of Trials Comparing the Two Combinations vs. Monotherapy. *American Journal of Hypertension* 2014; 27(2): 268-78.

565. Llorca PM, Lancon C, Brignone M, et al. Relative efficacy and tolerability of vortioxetine versus selected antidepressants by indirect comparisons of similar clinical studies. *Current Medical Research and Opinion* 2014; 30(12): 2589-606.

566. Loke YK, Pradhan S, Yeong JK, Kwok CS. Comparative coronary risks of apixaban, rivaroxaban and dabigatran: a meta-analysis and adjusted indirect comparison. *British Journal of Clinical Pharmacology*, 2014. http://onlinelibrary.wiley.com/o/cochrane/cldare/articles/DARE-12014019200/frame.html (accessed.

567. Loveman E, Copley VR, Colquitt JL, et al. The effectiveness and cost-effectiveness of treatments for idiopathic pulmonary fibrosis: systematic review, network meta-analysis and health economic evaluation. *Bmc Pharmacology & Toxicology* 2014; 15.

568. Loymans RJB, Gemperli A, Cohen J, et al. Comparative effectiveness of long term drug treatment strategies to prevent asthma exacerbations: network meta-analysis. *Bmj-British Medical Journal* 2014; 348.

569. Ma DF, Zhang ZJ, Zhang XR, Li LJ. Comparative efficacy, acceptability, and safety of medicinal, cognitive-behavioral therapy, and placebo treatments for acute major depressive disorder in children and adolescents: a multiple-treatments meta-analysis. *Current Medical Research and Opinion* 2014; 30(6): 971-95.

570. Maman K, Aballea S, Nazir J, et al. Comparative Efficacy and Safety of Medical Treatments for the Management of Overactive Bladder: A Systematic Literature Review and Mixed Treatment Comparison. *European Urology* 2014; 65(4): 755-65.

571. Mayo-Wilson E, Dias S, Mavranezouli I, et al. Psychological and pharmacological interventions for social anxiety disorder in adults: a systematic review and network meta-analysis. *Lancet Psychiatry* 2014; 1(5): 368-76.

572. Mealing S, Ghement I, Hawkins N, et al. The importance of baseline viral load when assessing relative efficacy in treatment-naive HBeAg-positive chronic hepatitis B: a systematic review and network meta-analysis *Systematic Reviews*, 2014. http://onlinelibrary.wiley.com/o/cochrane/cldare/articles/DARE-12014023437/frame.html (accessed.

573. Mercier F, Claret L, Prins K, Bruno R. A Model-Based Meta-analysis to Compare Efficacy and Tolerability of Tramadol and Tapentadol for the Treatment of Chronic Non-Malignant Pain. *Pain and Therapy* 2014; 3(1): 31-44.

574. Messori A, Fadda V, Maratea D, et al. Biological drugs for the treatment of moderate-to-severe psoriasis by subcutaneous route: determining statistical equivalence according to evidence-based methods. *Clin Drug Investig* 2014; 34(8): 593-8.

575. Messori A, Fadda V, Maratea D, Trippoli S, Marinai C. Anti-reabsorptive agents in women with osteoporosis: determining statistical equivalence according to evidence-based methods. *Journal of Endocrinological Investigation* 2014; 37(8): 769-73.

576. Michna E, Cheng WY, Korves C, et al. Systematic literature review and meta-analysis of the efficacy and safety of prescription opioids, including abuse-deterrent formulations, in non-cancer pain management. *Pain medicine (Malden, Mass)* 2014; 15(1): 79-92.

577. Mills EJ, Lester R, Thorlund K, et al. Interventions to promote adherence to antiretroviral therapy in Africa: A network meta-analysis. *The Lancet HIV* 2014; 1(3): e104-e11.

578. Mills EJ, Thorlund K, Eapen S, Wu P, Prochaska JJ. Cardiovascular Events Associated With Smoking Cessation Pharmacotherapies A Network Meta-Analysis. *Circulation* 2014; 129(1): 28-+.

579. Miura T, Noma H, Furukawa TA, et al. Comparative efficacy and tolerability of pharmacological treatments in the maintenance treatment of bipolar disorder: a systematic review and network meta-analysis. *Lancet Psychiatry* 2014; 1(5): 351-9.

580. Myers J, Wielage RC, Han B, et al. The efficacy of duloxetine, non-steroidal anti-inflammatory drugs, and opioids in osteoarthritis: A systematic literature review and meta-analysis. *BMC Musculoskeletal Disorders* 2014; 15(1).

581. Naci H, Dias S, Ades AE. Industry sponsorship bias in research findings: a network meta-analysis of LDL cholesterol reduction in randomised trials of statins. *Bmj-British Medical Journal* 2014; 349.

582. Nagayama A, Hayashida T, Jinno H, et al. Comparative Effectiveness of Neoadjuvant Therapy for HER2-Positive Breast Cancer: A Network Meta-Analysis. *Jnci-Journal of the National Cancer Institute* 2014; 106(9).

583. Nielsen PB, Lane DA, Rasmussen LH, Lip GYH, Larsen TB. Renal function and non-vitamin K oral anticoagulants in comparison with warfarin on safety and efficacy outcomes in atrial fibrillation patients: a systemic review and meta-regression analysis. *Clinical Research in Cardiology* 2014.

584. Nussbaumer B, Morgan LC, Reichenpfader U, et al. Comparative Efficacy and Risk of Harms of Immediate- versus Extended-Release Second-Generation Antidepressants: A Systematic Review with Network Meta-Analysis. *Cns Drugs* 2014; 28(8): 699-712.

585. Oba Y, Lone NA. Mortality benefit of vasopressor and inotropic agents in septic shock: A Bayesian network meta-analysis of randomized controlled trials. *Journal of Critical Care* 2014; 29(5): 706-10.

586. Oba Y, Lone NA. Comparative efficacy of inhaled corticosteroid and long-acting beta agonist combinations in preventing COPD exacerbations: a Bayesian network meta-analysis. *International Journal of Chronic Obstructive Pulmonary Disease* 2014; 9: 469-79.

587. Orme M, Fenici P, Lomon ID, Wygant G, Townsend R, Roudaut M. A systematic review and mixed-treatment comparison of dapagliflozin with existing anti-diabetes treatments for those with type 2 diabetes mellitus inadequately controlled by sulfonylurea monotherapy. *Diabetology & Metabolic Syndrome* 2014; 6.

588. Palmer SC, Saglimbene V, Mavridis D, et al. Erythropoiesis-stimulating agents for anaemia in adults with chronic kidney disease: a network meta-analysis. *Cochrane Database of Systematic Reviews* 2014; (12).

589. Palmerini T, Biondi-Zoccai G, Della Riva D, et al. Clinical Outcomes With Bioabsorbable Polymer-Versus Durable Polymer-Based Drug-Eluting and Bare-Metal Stents Evidence From a Comprehensive Network Meta-Analysis. *Journal of the American College of Cardiology* 2014; 63(4): 299-307.

590. Panagiotopoulou N, Nethra S, Karavolos S, Ahmad G, Karabis A, Burls A. Uterine-sparing minimally invasive interventions in women with uterine fibroids: a systematic review and indirect treatment comparison meta-analysis. *Acta Obstetricia Et Gynecologica Scandinavica* 2014; 93(9): 858-67.

591. Patel DA, Snedecor SJ, Tang WY, et al. 48-Week Efficacy and Safety of Dolutegravir Relative to Commonly Used Third Agents in Treatment-Naive HIV-1-Infected Patients: A Systematic Review and Network Meta-Analysis. *Plos One* 2014; 9(9).

592. Pechlivanoglou P, Le HH, Daenen S, Snowden JA, Postma MJ. Mixed treatment comparison of prophylaxis against invasive fungal infections in neutropenic patients receiving therapy for haematological malignancies: a systematic review. *Journal of Antimicrobial Chemotherapy* 2014; 69(1): 1-11.

593. Peruzzi M, De Luca L, Thomsen HS, et al. A Network Meta-Analysis on Randomized Trials Focusing on the Preventive Effect of Statins on Contrast-Induced Nephropathy. *Biomed Research International* 2014.

594. Philip F, Agarwal S, Bunte MC, et al. Stent Thrombosis With Second-Generation Drug-Eluting Stents Compared With Bare-Metal Stents Network Meta-Analysis of Primary Percutaneous Coronary Intervention Trials in ST-Segment-Elevation Myocardial Infarction. *Circulation-Cardiovascular Interventions* 2014; 7(1): 49-61.

595. Piccolo R, Galasso G, Piscione F, et al. Meta-Analysis of Randomized Trials Comparing the Effectiveness of Different Strategies for the Treatment of Drug-Eluting Stent Restenosis. *American Journal of Cardiology* 2014; 114(9): 1339-46.

596. Popat S, Mok T, Yang JCH, et al. Afatinib in the treatment of EGFR mutation-positive NSCLC - A network meta-analysis. *Lung Cancer* 2014; 85(2): 230-8.

597. Price R, MacLennan G, Glen J, Su DC. Selective digestive or oropharyngeal decontamination and topical oropharyngeal chlorhexidine for prevention of death in general intensive care: systematic review and network meta-analysis. *Bmj-British Medical Journal* 2014; 348.

598. Regnier S, Malcolm W, Allen F, Wright J, Bezlyak V. Efficacy of Anti-VEGF and Laser Photocoagulation in the Treatment of Visual Impairment due to Diabetic Macular Edema: A Systematic Review and Network Meta-Analysis. *Plos One* 2014; 9(7).

599. Reichenpfader U, Gartlehner G, Morgan LC, et al. Sexual Dysfunction associated with Second-Generation Antidepressants in Patients with Major Depressive Disorder: Results from a Systematic Review with Network Meta-Analysis. *Drug Safety* 2014; 37(1): 19-31.

600. Rheinheimer J, Ziegelmann PK, Carlessi R, et al. Different digestion enzymes used for human pancreatic islet isolation: A mixed treatment comparison (MTC) meta-analysis. *Islets* 2014; 6(4).

601. Rochwerg B, Alhazzani W, Sindi A, et al. Fluid Resuscitation in Sepsis A Systematic Review and Network Meta-analysis. *Annals of Internal Medicine* 2014; 161(5): 347-+.

602. Rollins BM, Silva MA, Donovan JL, Kanaan AO. Evaluation of Oral Anticoagulants for the Extended Treatment of Venous Thromboembolism Using a Mixed-Treatment Comparison, Meta-Analytic Approach. *Clinical Therapeutics* 2014; 36(10): 1454-64.

603. Roskell NS, Anzueto A, Hamilton A, Disse B, Becker K. Once-daily long-acting beta-agonists for chronic obstructive pulmonary disease: an indirect comparison of olodaterol and indacaterol. *International Journal of Chronic Obstructive Pulmonary Disease* 2014; 9.

604. Roskell NS, Setyawan J, Zimovetz EA, Hodgkins P. Systematic evidence synthesis of treatments for ADHD in children and adolescents: indirect treatment comparisons of lisdexamfetamine with methylphenidate and atomoxetine. *Current Medical Research and Opinion* 2014; 30(8): 1673-85.

605. Schwingshackl L, Missbach B, Dias S, Konig J, Hoffmann G. Impact of different training modalities on glycaemic control and blood lipids in patients with type 2 diabetes: a systematic review and network meta-analysis. *Diabetologia* 2014; 57(9): 1789-97.

606. Sekine L, Morais VD, Lima KM, Onsten TG, Ziegelmann PK, Ribeiro RA. Conventional and high-dose daunorubicin and idarubicin in acute myeloid leukaemia remission induction treatment: a mixed treatment comparison meta-analysis of 7258 patients *Database of Abstracts of Reviews of Effects*, 2014. http://onlinelibrary.wiley.com/o/cochrane/cldare/articles/DARE-12014065780/frame.html (accessed.

607. Shams T, Firwana B, Habib F, et al. SSRIs for Hot Flashes: A Systematic Review and Meta-Analysis of Randomized Trials. *Journal of General Internal Medicine* 2014; 29(1): 204-13.

608. Sheng Z, Zhang Y. EGFR-TKIs combined with chemotherapy versus EGFR-TKIs single agent as first-line treatment for molecularly selected patients with non-small cell lung cancer. *Medical Oncology* 2014; 32(1): 1-7.

609. Signorovitch J, Ayyagari R, Reichmann WM, Wu EQ, Chen L. Major molecular response during the first year of dasatinib, imatinib or nilotinib treatment for newly diagnosed chronic myeloid leukemia: A network meta-analysis. *Cancer Treatment Reviews* 2014; 40(2): 285-92.

610. Simillis C, Li TJ, Vaughan J, Becker LA, Davidson BR, Gurusamy KS. Methods to decrease blood loss during liver resection: a network meta-analysis. *Cochrane Database of Systematic Reviews* 2014; (4).

611. Singh S, Garg SK, Pardi DS, Wang Z, Murad MH, Loftus EV. Comparative Efficacy of Biologic Therapy in Biologic-Naive Patients With Crohn Disease: A Systematic Review and Network Meta-analysis. *Mayo Clinic Proceedings* 2014; 89(12): 1621-35.

612. Snedecor SJ, Sudharshan L, Cappelleri JC, et al. Systematic review and meta-analysis of pharmacological therapies for pain associated with postherpetic neuralgia and less common neuropathic conditions. *International Journal of Clinical Practice* 2014; 68(7): 900-18.

613. Snedecor SJ, Sudharshan L, Cappelleri JC, Sadosky A, Mehta S, Botteman M. Systematic Review and Meta-Analysis of Pharmacological Therapies for Painful Diabetic Peripheral Neuropathy. *Pain Practice* 2014; 14(2): 167-84.

614. Stagg HR, Zenner D, Harris RJ, Munoz L, Lipman MC, Abubakar I. Treatment of Latent Tuberculosis Infection A Network Meta-analysis. *Annals of Internal Medicine* 2014; 161(6): 419-U80.

615. Stidham RW, Lee TC, Higgins PD, et al. Systematic review with network meta-analysis: the efficacy of anti-tumour necrosis factor-alpha agents for the treatment of ulcerative colitis *Alimentary Pharmacology and Therapeutics*, 2014. http://onlinelibrary.wiley.com/o/cochrane/cldare/articles/DARE-12014012910/frame.html (accessed.

616. Stidham RW, Lee TCH, Higgins PDR, et al. Systematic review with network meta-analysis: the efficacy of anti-TNF agents for the treatment of Crohn's disease. *Alimentary Pharmacology & Therapeutics* 2014; 39(12): 1349-62.

617. Sun Y, van Valkenhoef G, Morel T. A mixed treatment comparison of gabapentin enacarbil, pramipexole, ropinirole and rotigotine in moderate-to-severe restless legs syndrome. *Current Medical Research and Opinion* 2014; 30(11): 2267-78.

618. Tadrous M, Wong L, Mamdani MM, et al. Comparative gastrointestinal safety of bisphosphonates in primary osteoporosis: a network meta-analysis. *Osteoporosis International* 2014; 25(4): 1225-35.

619. Takeuchi M. Bayesian network meta-analysis suggests a similar effectiveness between a monovalent and a pentavalent rotavirus vaccine A preliminary report of re-analyses of data from a Cochrane Database Systematic Review. *Human Vaccines & Immunotherapeutics* 2014; 10(5): 1421-4.

620. Tan PS, Haaland B, Montero AJ, Kyriakopoulos CE, Lopes G. Hormonal therapeutics enzalutamide and abiraterone acetate in the treatment of metastatic castration-resistant prostate cancer (mCRPC) post-docetaxel-an indirect comparison. *Clinical Medicine Insights: Oncology* 2014; 8: 29-36.

621. Taylor DM, Cornelius V, Smith L, Young AH. Comparative efficacy and acceptability of drug treatments for bipolar depression: a multiple-treatments meta-analysis. *Acta Psychiatrica Scandinavica* 2014; 130(6): 452-69.

622. Thom HHZ, Capkun G, Nixon RM, Ferreira A. Indirect comparisons of ranibizumab and dexamethasone in macular oedema secondary to retinal vein occlusion. *Bmc Medical Research Methodology* 2014; 14.

623. Thorlund K, Druyts E, Mills EJ, Fedorak RN, Marshall JK. Adalimumab versus infliximab for the treatment of moderate to severe ulcerative colitis in adult patients naive to anti-TNF therapy: An indirect treatment comparison meta-analysis. *Journal of Crohn's & colitis* 2014.

624. Thorlund K, Mills EJ, Wu P, et al. Comparative efficacy of triptans for the abortive treatment of migraine: A multiple treatment comparison meta-analysis. *Cephalalgia* 2014; 34(4): 258-67.

625. Thorlund K, Wu P, Druyts E, Eapen S, Mills EJ. Nonergot dopamine-receptor agonists for treating Parkinson's disease - a network meta-analysis. *Neuropsychiatric Disease and Treatment* 2014; 10: 767-76.

626. Toulis KA, Hemming K, Stergianos S, Nirantharakumar K, Bilezikian JP. beta-Adrenergic receptor antagonists and fracture risk: a meta-analysis of selectivity, gender, and site-specific effects. *Osteoporosis international : a journal established as result of cooperation between the European Foundation for Osteoporosis and the National Osteoporosis Foundation of the USA* 2014; 25(1): 121-9.

627. Tricco AC, Antony J, Khan PA, et al. Safety and effectiveness of dipeptidyl peptidase-4 inhibitors versus intermediate-acting insulin or placebo for patients with type 2 diabetes failing two oral antihyperglycaemic agents: a systematic review and network meta-analysis. *Bmj Open* 2014; 4(12).

628. Tricco AC, Ashoor HM, Antony J, et al. Safety, effectiveness, and cost effectiveness of long acting versus intermediate acting insulin for patients with type 1 diabetes: systematic review and network meta-analysis. *Bmj-British Medical Journal* 2014; 349.

629. Tu B, Rich B, Labos C, Brophy JM. Coronary Revascularization in Diabetic Patients A Systematic Review and Bayesian Network Meta-analysis. *Annals of Internal Medicine* 2014; 161(10): 724-U179.

630. Vegter S, Tolley K. A Network Meta-Analysis of the Relative Efficacy of Treatments for Actinic Keratosis of the Face or Scalp in Europe. *Plos One* 2014; 9(6).

631. Verdecchia P, Angeli F, Lip GYH, Reboldi G. Edoxaban in the Evolving Scenario of Non Vitamin K Antagonist Oral Anticoagulants Imputed Placebo Analysis and Multiple Treatment Comparisons. *Plos One* 2014; 9(6).

632. Wang HT, Yuan JQ, Hu XL, Tao K, Liu JQ, Hu DH. The effectiveness and safety of avanafil for erectile dysfunction: a systematic review and meta-analysis. *Current Medical Research and Opinion* 2014; 30(8): 1565-71.

633. Wang JC, Tian JH, Ge L, Gan YH, Yang KH. Which is the Best Chinese Herb Injection Based on the FOLFOX Regimen for Gastric Cancer? A Network Meta-analysis of Randomized Controlled Trials. *Asian Pacific Journal of Cancer Prevention* 2014; 15(12): 4795-800.

634. Wang X, Huang S, Qi HB. Comparative efficacy and acceptability of seven augmentation agents for treatment-resistant depression: A multiple-treatments meta-analysis. *South African Journal of Psychiatry* 2014; 20(3): 71-6.

635. Wang XH, Wang X, Li S, Meng Z, Liu T, Zhang XH. Comparative Effectiveness of Oral Drug Therapies for Lower Urinary Tract Symptoms due to Benign Prostatic Hyperplasia: A Systematic Review and Network Meta-Analysis. *Plos One* 2014; 9(9).

636. Wertli MM, Kessels AG, Perez RS, Bachmann LM, Brunner F. Rational pain management in complex regional pain syndrome 1 (CRPS 1)--a network meta-analysis. *Pain medicine (Malden, Mass)* 2014; 15(9): 1575-89.

637. Windecker S, Stortecky S, Stefanini GG, et al. Revascularisation versus medical treatment in patients with stable coronary artery disease: network meta-analysis. *Bmj-British Medical Journal* 2014; 348.

638. Xiong TB, Turner RM, Wei YH, Neal DE, Lyratzopoulos G, Higgins JPT. Comparative efficacy and safety of treatments for localised prostate cancer: an application of network meta-analysis. *Bmj Open* 2014; 4(5).

639. Yang BW, Shi JP, Chen X, Ma B, Sun H. Efficacy and Safety of Therapies for Acute Ischemic Stroke in China: A Network Meta-Analysis of 13289 Patients from 145 Randomized Controlled Trials. *Plos One* 2014; 9(2).

640. Yang ZP, Ye XF, Wu Q, Wu KC, Fan DM. A network meta-analysis on the efficacy of 5-aminosalicylates, immunomodulators and biologics for the prevention of postoperative recurrence in Crohn's disease. *International Journal of Surgery* 2014; 12(5): 516-22.

641. Ye YC, Xie HZ, Zeng Y, Zhao XL, Tian Z, Zhang SY. Optimal Oral Antithrombotic Regimes for Patients with Acute Coronary Syndrome: A Network Meta-Analysis. *Plos One* 2014; 9(3).

642. Zaccara G, Giovannelli F, Bell GS, Sander JW. Network meta-analyses of antiepileptic drug efficacy and tolerability in drug-resistant focal epilepsies: a clinical perspective. *European Journal of Clinical Pharmacology* 2014; 70(6): 647-54.

643. Zafari Z, Thorlund K, FitzGerald JM, Marra CA, Sadatsafavi M. Network vs. Pairwise Meta-Analyses: A Case Study of the Impact of an Evidence-Synthesis Paradigm on Value of Information Outcomes. *Pharmacoeconomics* 2014; 32(10): 995-1004.

644. Zagmutt FJ, Carroll CA. Meta-analysis of adverse events in recent randomized clinical trials for dimethil fumarate, glatiramer acetate, and teriflunomide for the treatment of relapsing forms of multiple sclerosis *Database of Abstracts of Reviews of Effects*, 2014. http://onlinelibrary.wiley.com/o/cochrane/cldare/articles/DARE-12014067693/frame.html (accessed.

645. Zeng C, Li H, Yang T, et al. Effectiveness of continuous and pulsed ultrasound for the management of knee osteoarthritis: a systematic review and network meta-analysis. *Osteoarthritis and Cartilage* 2014; 22(8): 1090-9.

646. Zeppetella G, Davies A, Eijgelshoven I, Jansen JP. A Network Meta-Analysis of the Efficacy of Opioid Analgesics for the Management of Breakthrough Cancer Pain Episodes. *Journal of Pain and Symptom Management* 2014; 47(4): 772-+.

647. Zhang Y, Sheng J, Kang S, et al. Patients with exon 19 deletion were associated with longer progression-free survival compared to those with L858R mutation after first-line EGFR-TKIs for advanced non-small cell lung cancer: a meta-analysis. *PLoS One* 2014; 9(9): e107161.

648. Zhang YW, Zhang YL, Pan H, et al. Chemotherapy for patients with gastric cancer after complete resection: A network meta-analysis. *World Journal of Gastroenterology* 2014; 20(2): 584-92.

649. Zheng H, Barnett AG, Merollini K, et al. Control strategies to prevent total hip replacement-related infections: a systematic review and mixed treatment comparison. *Bmj Open* 2014; 4(3).

650. Zhu GQ, Shi KQ, You J, et al. Systematic review with network meta-analysis: adjuvant therapy for resected biliary tract cancer. *Alimentary Pharmacology & Therapeutics* 2014; 40(7): 759-70.

651. Zhu Z, Zhang J, Liu Y, Chen M, Guo P, Li K. Efficacy and toxicity of external-beam radiation therapy for localised prostate cancer: a network meta-analysis. *British Journal of Cancer* 2014; 110(10): 2396-404.

652. Ziakas PD, Zervou FN, Zacharioudakis IM, Mylonakis E. Graft-Versus-Host Disease Prophylaxis after Transplantation: A Network Meta-Analysis. *Plos One* 2014; 9(12).

653. Zintzaras E, Miligkos M, Ziakas P, et al. Assessment of the Relative Effectiveness and Tolerability of Treatments of Type 2 Diabetes Mellitus: A Network Meta-analysis. *Clinical Therapeutics* 2014; 36(10): 1443-53.

654. Achana FA, Sutton AJ, Kendrick D, et al. The Effectiveness of Different Interventions to Promote Poison Prevention Behaviours in Households with Children: A Network Meta-Analysis. *Plos One* 2015; 10(4).

655. Alfirevic Z, Keeney E, Dowswell T, et al. Labour induction with prostaglandins: a systematic review and network meta-analysis. *Bmj-British Medical Journal* 2015; 350.

656. Antoniou SA, Morales-Conde S, Antoniou GA, Pointner R, Granderath FA. Single-incision laparoscopic cholecystectomy with curved versus linear instruments assessed by systematic review and network meta-analysis of randomized trials. *Surgical Endoscopy and Other Interventional Techniques* 2015.

657. Arirachakaran A, Sangkaew C, Kongtharvonskul J. Patellofemoral resurfacing and patellar denervation in primary total knee arthroplasty. *Knee Surgery Sports Traumatology Arthroscopy* 2015; 23(6): 1770-81.

658. Bannuru RR, Schmid CH, Kent DM, Vaysbrot EE, Wong JB, McAlindon TE. Comparative Effectiveness of Pharmacologic Interventions for Knee Osteoarthritis A Systematic Review and Network Meta-analysis. *Annals of Internal Medicine* 2015; 162(1): 46-U189.

659. Benedetto U, Raja SG, Albanese A, Amrani M, Biondi-Zoccai G, Frati G. Searching for the second best graft for coronary artery bypass surgery: a network meta-analysis of randomized controlled trials. *European Journal of Cardio-Thoracic Surgery* 2015; 47(1): 59-65.

660. Bow EJ, Vanness DJ, Slavin M, et al. Systematic review and mixed treatment comparison meta-analysis of randomized clinical trials of primary oral antifungal prophylaxis in allogeneic hematopoietic cell transplant recipients. *Bmc Infectious Diseases* 2015; 15.

661. Buckley F, Finckh A, Huizinga TWJ, Dejonckheere F, Jansen JP. Comparative efficacy of novel DMARDs as monotherapy and in combination with methotrexate in rheumatoid arthritis patients with inadequate response to conventional DMARDs: A network meta-analysis. *Journal of Managed Care Pharmacy* 2015; 21(5): 409-23.

662. Bulluck H, Kwok CS, Ryding AD, Loke YK. Safety of short-term dual antiplatelet therapy after drug-eluting stents: An updated meta-analysis with direct and adjusted indirect comparison of randomized control trials. *International Journal of Cardiology* 2015; 181: 331-9.

663. Chen L, Staubli SEL, Schneider MP, et al. Phosphodiesterase 5 Inhibitors for the Treatment of Erectile Dysfunction: A Trade-off Network Meta-analysis. *European Urology* 2015.

664. Chen LX, Li YL, Ning GZ, et al. Comparative Efficacy and Tolerability of Three Treatments in Old People with Osteoporotic Vertebral Compression Fracture: A Network Meta-Analysis and Systematic Review. *Plos One* 2015; 10(4).

665. Chen LX, Zhou ZR, Li YL, et al. Comparison of Bone Mineral Density in Lumbar Spine and Fracture Rate among Eight Drugs in Treatments of Osteoporosis in Men: A Network Meta-Analysis. *Plos One* 2015; 10(5).

666. Chen YP, Wang ZX, Chen L, et al. A Bayesian network meta-analysis comparing concurrent chemoradiotherapy followed by adjuvant chemotherapy, concurrent chemoradiotherapy alone and radiotherapy alone in patients with locoregionally advanced nasopharyngeal carcinoma. *Annals of Oncology* 2015; 26(1): 205-11.

667. Cure S, Bianic F, Espinas C, Hardy H, Rosenblatt L, Juday T. Systematic literature review and meta-analysis of renal function in human immunodeficiency virus (HIV)-infected patients treated with atazanavir (ATV)-based regimens. *PLoS ONE* 2015; 10(5).

668. Dong W, Goost H, Lin XB, et al. Treatments for Shoulder Impingement Syndrome A PRISMA Systematic Review and Network Meta-Analysis. *Medicine* 2015; 94(10).

669. Druyts E, Lorenzi M, Toor K, Thorlund K, Mills EJ. Network meta-analysis of direct-acting antivirals in combination with peginterferon-ribavirin for previously untreated patients with hepatitis C genotype 1 infection. *Qjm-an International Journal of Medicine* 2015; 108(4): 299-306.

670. Dumville Jo C, McFarlane E, Edwards P, Lipp A, Holmes A, Liu Z. Preoperative skin antiseptics for preventing surgical wound infections after clean surgery. *Cochrane Database of Systematic Reviews*, 2015. http://onlinelibrary.wiley.com/doi/10.1002/14651858.CD003949.pub4/abstract (accessed.

671. Edwards SJ, Barton S, Thurgar E, Trevor N. Topotecan, pegylated liposomal doxorubicin hydrochloride, paclitaxel, trabectedin and gemcitabine for advanced recurrent or refractory ovarian cancer: a systematic review and economic evaluation. *Health Technology Assessment* 2015; 19(7): 1-+.

672. Elmunzer BJ, Singal AG, Sussman JB, et al. Comparing the effectiveness of competing tests for reducing colorectal cancer mortality: a network meta-analysis. *Gastrointestinal Endoscopy* 2015; 81(3): 700-U642.

673. Fadda V, Maratea D, Trippoli S, Messori A. Gastrointestinal and renal side effects of bisphosphonates: differentiating between no proof of difference and proof of no difference. *Journal of Endocrinological Investigation* 2015; 38(2): 189-92.

674. Foote CJ, Guyatt GH, Vignesh KN, et al. Which Surgical Treatment for Open Tibial Shaft Fractures Results in the Fewest Reoperations? A Network Meta-analysis. *Clinical Orthopaedics and Related Research* 2015; 473(7): 2179-92.

675. Gao L, Zhao FL, Li SC. Efficacy and Safety of Thrombin-Receptor Antagonist (Atopaxar and Vorapaxar) in Patients with Acute Coronary Syndrome or Coronary Artery Disease-A Meta-Analysis of Randomized Controlled Trials. *Value in Health Regional Issues* 2015; 6: 22-32.

676. Generali D, Venturini S, Rognoni C, et al. A network meta-analysis of everolimus plus exemestane versus chemotherapy in the first- and second-line treatment of estrogen receptor-positive metastatic breast cancer. *Breast Cancer Research and Treatment* 2015; 152(1): 95-117.

677. Greco T, Calabro MG, Covello RD, et al. A Bayesian network meta-analysis on the effect of inodilatory agents on mortality. *British Journal of Anaesthesia* 2015; 114(5): 746-56.

678. Gu S, Shi J, Tang Z, et al. Comparison of glucose lowering effect of metformin and acarbose in type 2 diabetes mellitus: A meta-analysis. *PLoS ONE* 2015; 10(5).

679. Gupta T, Kannan S, Ghosh-Laskar S, Agarwal JP. Concomitant chemoradiotherapy versus altered fractionation radiotherapy in the radiotherapeutic management of locoregionally advanced head and neck squamous cell carcinoma: An adjusted indirect comparison meta-analysis. *Head and Neck-Journal for the Sciences and Specialties of the Head and Neck* 2015; 37(5): 670-6.

680. Ha EJ, Baek JH, Kim KW, et al. Comparative efficacy of radiofrequency and laser ablation for the treatment of benign thyroid nodules: Systematic review including traditional pooling and Bayesian network meta-analysis. *Journal of Clinical Endocrinology and Metabolism* 2015; 100(5): 1903-11.

681. Haspinger ER, Agustoni F, Torri V, et al. Is there evidence for different effects among EGFR-TKIs? Systematic review and meta-analysis of EGFR tyrosine kinase inhibitors (TKIs) versus chemotherapy as first-line treatment for patients harboring EGFR mutations. *Critical Reviews in Oncology/Hematology* 2015; 94(2): 213-27.

682. Hazlewood GS, Rezaie A, Borman M, et al. Comparative Effectiveness of Immunosuppressants and Biologics for Inducing and Maintaining Remission in Crohn's Disease: A Network Meta-analysis. *Gastroenterology* 2015; 148(2): 344-U134.

683. Huai ZY, Xian WF, Jiang LC, Chen WX. Submucosal Injection Solution for Endoscopic Resection in Gastrointestinal Tract: A Traditional and Network Meta-Analysis. *Gastroenterology Research and Practice* 2015.

684. Huang X, Xu B. Efficacy and Safety of Tacrolimus versus Pimecrolimus for the Treatment of Atopic Dermatitis in Children: A Network Meta-Analysis. *Dermatology* 2015.

685. Hubbard S, Cooper N, Kendrick D, et al. Network meta-analysis to evaluate the effectiveness of interventions to prevent falls in children under age 5 years. *Injury Prevention* 2015; 21(2): 98-108.

686. Huttner FJ, Tenckhoff S, Jensen K, et al. Meta-analysis of reconstruction techniques after low anterior resection for rectal cancer. *British Journal of Surgery* 2015; 102(7): 735-45.

687. Jiang YF, Chen XY, Ding T, Wang XF, Zhu ZN, Su SW. Comparative efficacy and safety of OADs in management of GDM: Network meta-analysis of randomized controlled trials. *Journal of Clinical Endocrinology and Metabolism* 2015; 100(5): 2071-80.

688. Kelly ME, Spolverato G, Le GN, et al. Synchronous Colorectal Liver Metastasis: A Network Meta-Analysis Review Comparing Classical, Combined, and Liver-First Surgical Strategies. *Journal of Surgical Oncology* 2015; 111(3): 341-51.

689. Kongtharvonskul J, Anothaisintawee T, McEvoy M, Attia J, Woratanarat P, Thakkinstian A. Efficacy and safety of glucosamine, diacerein, and NSAIDs in osteoarthritis knee: a systematic review and network meta-analysis. *European Journal of Medical Research* 2015; 20.

690. Kotb A, Cameron C, Hsieh S, Wells G. Comparative Effectiveness of Different Forms of Telemedicine for Individuals with Heart Failure (HF): A Systematic Review and Network Meta-Analysis. *Plos One* 2015; 10(2).

691. Kumagai K, Rouvelas I, Tsai JA, et al. Survival benefit and additional value of preoperative chemoradiotherapy in resectable gastric and gastro-oesophageal junction cancer: A direct and adjusted indirect comparison meta-analysis. *Ejso* 2015; 41(3): 282-94.

692. Kwon JK, Cho KS, Oh CK, et al. The beneficial effect of alpha-blockers for ureteral stent-related discomfort: Systematic review and network meta-analysis for alfuzosin versus tamsulosin versus placebo. *BMC Urology* 2015.

693. Ladyzynski P, Molik M, Foltynski P. A network meta-analysis of progression free survival and overall survival in first-line treatment of chronic lymphocytic leukemia. *Cancer Treatment Reviews* 2015; 41(2): 77-93.

694. Lee JM, Park J, Kang J, et al. Comparison Among Drug-Eluting Balloon, Drug-Eluting Stent, and Plain Balloon Angioplasty for the Treatment of In-Stent Restenosis A Network Meta-Analysis of 11 Randomized, Controlled Trials. *Jacc-Cardiovascular Interventions* 2015; 8(3): 382-94.

695. Lee JM, Park J, Kang J, et al. The efficacy and safety of mechanical hemodynamic support in patients undergoing high-risk percutaneous coronary intervention with or without cardiogenic shock: Bayesian approach network meta-analysis of 13 randomized controlled trials. *International Journal of Cardiology* 2015; 184: 36-46.

696. Lee YH, Bae SC, Song GG. Comparative efficacy and safety of tofacitinib, with or without methotrexate, in patients with active rheumatoid arthritis: a Bayesian network meta-analysis of randomized controlled trials. *Rheumatology International* 2015.

697. Lewis RA, Williams NH, Sutton AJ, et al. Comparative clinical effectiveness of management strategies for sciatica: systematic review and network meta-analyses. *Spine Journal* 2015; 15(6): 1461-77.

698. Lhermusier T, Lipinski MJ, Tantry US, et al. Meta-Analysis of Direct and Indirect Comparison of Ticagrelor and Prasugrel Effects on Platelet Reactivity. *American Journal of Cardiology* 2015; 115(6): 716-23.

699. Linde K, Kriston L, Rucker G, et al. Efficacy and Acceptability of Pharmacological Treatments for Depressive Disorders in Primary Care: Systematic Review and Network Meta-Analysis. *Annals of Family Medicine* 2015; 13(1): 69-79.

700. Liu M, Zhang H, Du BX, et al. Neurokinin-1 Receptor Antagonists in Preventing Postoperative Nausea and Vomiting A Systematic Review and Meta-Analysis. *Medicine* 2015; 94(19).

701. Loveman E, Copley VR, Scott DA, Colquitt JL, Clegg AJ, O'Reilly KMA. Comparing new treatments for idiopathic pulmonary fibrosis - a network meta-analysis. *Bmc Pulmonary Medicine* 2015; 15.

702. Maxwell Lara J, Zochling J, Boonen A, et al. TNF-alpha inhibitors for ankylosing spondylitis. *Cochrane Database of Systematic Reviews*, 2015. http://onlinelibrary.wiley.com/doi/10.1002/14651858.CD005468.pub2/abstract (accessed.

703. Mazaki T, Ishii Y, Murai I. Immunoenhancing Enteral and Parenteral Nutrition for Gastrointestinal Surgery A Multiple-treatments Meta-analysis. *Annals of Surgery* 2015; 261(4): 662-9.

704. Mearns ES, Sobieraj DM, White CM, et al. Comparative Efficacy and Safety of Antidiabetic Drug Regimens Added to Metformin Monotherapy in Patients with Type 2 Diabetes: A Network Meta-Analysis. *Plos One* 2015; 10(4).

705. Mei WQ, Hu HZ, Liu Y, Li ZC, Wang WG. Infliximab is superior to other biological agents for treatment of active ulcerative colitis: A meta-analysis. *World Journal of Gastroenterology* 2015; 21(19): 6044-51.

706. Messori A, Fadda V, Maratea D, Trippoli S. First-line treatments for chronic lymphocytic leukaemia: interpreting efficacy data by network meta-analysis. *Annals of Hematology* 2015; 94(6): 1003-9.

707. Messori A, Fadda V, Maratea D, Trippoli S, Marinai C. Testing the therapeutic equivalence of novel oral anticoagulants for thromboprophylaxis in orthopedic surgery and for prevention of stroke in atrial fibrillation. *International Journal of Clinical Pharmacology and Therapeutics* 2015; 53(3): 211-9.

708. Migliore A, Bizzi E, Bernardi M, Diamanti AP, Lagana B, Petrella L. Indirect Comparison Between Subcutaneous Biologic Agents in Ankylosing Spondylitis. *Clinical Drug Investigation* 2015; 35(1): 23-9.

709. Moja L, Danese S, Fiorino G, Del Giovane C, Bonovas S. Systematic review with network meta-analysis: comparative efficacy and safety of budesonide and mesalazine (mesalamine) for Crohn's disease. *Alimentary Pharmacology & Therapeutics* 2015; 41(11): 1055-65.

710. Nelson H, Cartier S, Allen-Ramey F, Lawton S, Calderon MA. Network Meta-analysis Shows Commercialized Subcutaneous and Sublingual Grass Products Have Comparable Efficacy. *Journal of Allergy and Clinical Immunology-in Practice* 2015; 3(2): 256-+.

711. Nielsen RE, Levander S, Kjaersdam Telleus G, Jensen SOW, Ostergaard Christensen T, Leucht S. Second-generation antipsychotic effect on cognition in patients with schizophrenia-a meta-analysis of randomized clinical trials. *Acta Psychiatrica Scandinavica* 2015; 131(3): 185-96.

712. Oba Y, Lone NA. Comparative efficacy of long-acting muscarinic antagonists in preventing COPD exacerbations: a network meta-analysis and meta-regression. *Therapeutic Advances in Respiratory Disease* 2015; 9(1): 3-15.

713. Oh GH, Yu JC, Choi KS, Joo EJ, Jeong SH. Simultaneous Comparison of Efficacy and Tolerability of Second-Generation Antipsychotics in Schizophrenia: Mixed-Treatment Comparison Analysis Based on Head-to-Head Trial Data. *Psychiatry Investigation* 2015; 12(1): 46-54.

714. Palmer SC, Mavridis D, Navarese E, et al. Comparative efficacy and safety of blood pressure-lowering agents in adults with diabetes and kidney disease: a network meta-analysis. *Lancet* 2015; 385(9982): 2047-56.

715. Palmerini T, Benedetto U, Bacchi-Reggiani L, et al. Mortality in patients treated with extended duration dual antiplatelet therapy after drug-eluting stent implantation: a pairwise and Bayesian network meta-analysis of randomised trials. *Lancet* 2015; 385(9985): 2371-82.

716. Palmerini T, Benedetto U, Biondi-Zoccai G, et al. Long-Term Safety of Drug-Eluting and Bare-Metal Stents Evidence From a Comprehensive Network Meta-Analysis. *Journal of the American College of Cardiology* 2015; 65(23): 2496-507.

717. Palmerini T, Sangiorgi D, Valgimigli M, et al. Short- Versus Long-Term Dual Antiplatelet Therapy After Drug-Eluting Stent Implantation An Individual Patient Data Pairwise and Network Meta-Analysis. *Journal of the American College of Cardiology* 2015; 65(11): 1092-102.

718. Pilkington G, Boland A, Brown T, Oyee J, Bagust A, Dickson R. A systematic review of the clinical effectiveness of first-line chemotherapy for adult patients with locally advanced or metastatic non-small cell lung cancer. *Thorax* 2015; 70(4): 359-67.

719. Police RL, Trask PC, Wang J, et al. Randomized controlled trials in relapsed/refractory chronic lymphocytic leukemia: A systematic review and meta-analysis. *Clinical Lymphoma, Myeloma and Leukemia* 2015; 15(4): 199-207.

720. Popat S, Mellemgaard A, Fahrbach K, et al. Nintedanib plus docetaxel as second-line therapy in patients with non-small-cell lung cancer: a network meta-analysis. *Future Oncology* 2015; 11(3): 409-20.

721. Quigley JM, Bryden PA, Scott DA, Kuwabara H, Cerri K. Relative efficacy and safety of simeprevir and telaprevir in treatment-naive hepatitis C-infected patients in a Japanese population: A Bayesian network meta-analysis. *Hepatology Research* 2015.

722. Regnier SA, Larsen M, Bezlyak V, Allen F. Comparative efficacy and safety of approved treatments for macular oedema secondary to branch retinal vein occlusion: A network meta-analysis. *BMJ Open* 2015; 5(6).

723. Ren Z, Qin L, Wang JQ, Li Y, Li J, Zhang RG. Comparative Efficacy of Four Treatments in Patients with Graves' Disease: a Network Meta-analysis. *Experimental and Clinical Endocrinology & Diabetes* 2015; 123(5): 317-22.

724. Rochwerg B, Alhazzani W, Gibson A, et al. Fluid type and the use of renal replacement therapy in sepsis: a systematic review and network meta-analysis. *Intensive Care Medicine* 2015.

725. Schmid MK, Bachmann LM, Fas L, Kessels AG, Job OM, Thiel MA. Efficacy and adverse events of aflibercept, ranibizumab and bevacizumab in age-related macular degeneration: a trade-off analysis. *British Journal of Ophthalmology* 2015; 99(2): 141-6.

726. Schwendicke F, Jager AM, Paris S, Hsu LY, Tu YK. Treating Pit-and-Fissure Caries: A Systematic Review and Network Meta-analysis. *Journal of Dental Research* 2015; 94(4): 522-33.

727. Schwendicke F, Paris S, Tu YK. Effects of using different criteria for caries removal: A systematic review and network meta-analysis. *Journal of Dentistry* 2015; 43(1): 1-15.

728. Schwingshackl L, Dias S, Hoffmann G. Impact of long-term lifestyle programmes on weight loss and cardiovascular risk factors in overweight/obese participants: A systematic review and network meta-analysis. *Systematic Reviews* 2015; 3(1).

729. Signorovitch JE, Betts KA, Yan YS, et al. Comparative efficacy of biological treatments for moderate-to-severe psoriasis: a network meta-analysis adjusting for cross-trial differences in reference arm response. *British Journal of Dermatology* 2015; 172(2): 504-12.

730. Simpson SH, Lee J, Choi S, Vandermeer B, Abdelmoneim AS, Featherstone TR. Mortality risk among sulfonylureas: a systematic review and network meta-analysis. *Lancet Diabetes & Endocrinology* 2015; 3(1): 43-51.

731. Singh JA, Cameron C, Noorbaloochi S, et al. Risk of serious infection in biological treatment of patients with rheumatoid arthritis: A systematic review and meta-analysis. *The Lancet* 2015.

732. Singh S, Garg SK, Pardi DS, Wang Z, Murad MH, Loftus EV. Comparative Efficacy of Pharmacologic Interventions in Preventing Relapse of Crohn's Disease After Surgery: A Systematic Review and Network Meta-analysis. *Gastroenterology* 2015; 148(1): 64-U485.

733. Sobieraj DM, Coleman CI, Pasupuleti V, Deshpande A, Kaw R, Hernandez AV. Comparative efficacy and safety of anticoagulants and aspirin for extended treatment of venous thromboembolism: A network meta-analysis. *Thrombosis Research* 2015; 135(5): 888-96.

734. Stortecky S, da Costa BR, Mattle HP, et al. Percutaneous closure of patent foramen ovale in patients with cryptogenic embolism: a network meta-analysis. *European Heart Journal* 2015; 36(2): 120-U29.

735. Stynes G, Svedsater H, Wex J, et al. Once-daily fluticasone furoate/vilanterol 100/25 mcg versus twice daily combination therapies in COPD - mixed treatment comparisons of clinical efficacy. *Respiratory Research* 2015; 16.

736. Sun F, Chai SB, Li LS, et al. Effects of Glucagon-Like Peptide-1 Receptor Agonists on Weight Loss in Patients with Type 2 Diabetes: A Systematic Review and Network Meta-Analysis. *Journal of Diabetes Research* 2015.

737. Sun F, Chai SB, Yu K, et al. Gastrointestinal Adverse Events of Glucagon-Like Peptide-1 Receptor Agonists in Patients with Type 2 Diabetes: A Systematic Review and Network Meta-Analysis. *Diabetes Technology & Therapeutics* 2015; 17(1): 35-42.

738. Sun F, Wu SS, Guo SX, et al. Effect of GLP-1 receptor agonists on waist circumference among type 2 diabetes patients: a systematic review and network meta-analysis. *Endocrine* 2015; 48(3): 794-803.

739. Sun F, Wu SS, Wang J, et al. Effect of Glucagon-like Peptide-1 Receptor Agonists on Lipid Profiles Among Type 2 Diabetes: A Systematic Review and Network Meta-analysis. *Clinical Therapeutics* 2015; 37(1): 225-41.

740. Tang J, Zhang H, Yan J, Shao R. Indirect comparison of the efficacy and safety of gefitinib and cetuximab-based therapy in patients with advanced non-small-cell lung cancer. *Molecular and Clinical Oncology* 2015; 3(1): 145-50.

741. Thakur D, Dickerson S, Bhutani MK, Junor R. Impact of Prolonged-release Oxycodone/Naloxone on Outcomes Affecting Patients' Daily Functioning in Comparison With Extended-release Tapentadol: A Systematic Review. *Clinical Therapeutics* 2015; 37(1): 212-24.

742. Thom HHZ, Capkun G, Cerulli A, Nixon RM, Howard LS. Network meta-analysis combining individual patient and aggregate data from a mixture of study designs with an application to pulmonary arterial hypertension. *Bmc Medical Research Methodology* 2015; 15.

743. Thorlund K, Druyts E, Toor K, Mills EJ. Comparative efficacy of golimumab, infliximab, and adalimumab for moderately to severely active ulcerative colitis: a network meta-analysis accounting for differences in trial designs. *Expert Review of Gastroenterology & Hepatology* 2015; 9(5): 693-700.

744. Thorlund K, Druyts E, Wu P, Balijepalli C, Keohane D, Mills E. Comparative Efficacy and Safety of Selective Serotonin Reuptake Inhibitors and Serotonin-Norepinephrine Reuptake Inhibitors in Older Adults: A Network Meta-Analysis. *Journal of the American Geriatrics Society* 2015; 63(5): 1002-9.

745. Tiancha H, Jiyong J, Min Y. How to Promote Bedside Placement of the Postpyloric Feeding Tube: A Network Meta-Analysis of Randomized Controlled Trials. *Journal of Parenteral and Enteral Nutrition* 2015; 39(5): 521-30.

746. Tolley K, Hutchinson M, You XJ, et al. A Network Meta-Analysis of Efficacy and Evaluation of Safety of Subcutaneous Pegylated Interferon Beta-1a versus Other Injectable Therapies for the Treatment of Relapsing-Remitting Multiple Sclerosis. *Plos One* 2015; 10(6).

747. Tricco AC, Soobiah C, Blondal E, et al. Comparative safety of serotonin (5-HT<inf>3</inf>) receptor antagonists in patients undergoing surgery: A systematic review and network meta-analysis. *BMC Medicine* 2015.

748. van Walsem A, Pandhi S, Nixon RM, Guyot P, Karabis A, Moore RA. Relative benefit-risk comparing diclofenac to other traditional non-steroidal anti-inflammatory drugs and cyclooxygenase-2 inhibitors in patients with osteoarthritis or rheumatoid arthritis: a network meta-analysis. *Arthritis Research & Therapy* 2015; 17.

749. Vemulapalli S, Dolor RJ, Hasselblad V, et al. Comparative Effectiveness of Medical Therapy, Supervised Exercise, and Revascularization for Patients with Intermittent Claudication: A Network Meta-analysis. *Clinical Cardiology* 2015; 38(6): 378-86.

750. Wang B, Wang F, Zhang Y, et al. Effects of RAS inhibitors on diabetic retinopathy: a systematic review and meta-analysis. *Lancet Diabetes & Endocrinology* 2015; 3(4): 263-74.

751. Wang C, Zhao N, Wang W, et al. Intraoperative mechanical ventilation strategies for obese patients: a systematic review and network meta-analysis. *Obesity Reviews* 2015; 16(6): 508-17.

752. Wang CS, Guo LB, Chi CJ, et al. Mechanical ventilation modes for respiratory distress syndrome in infants: a systematic review and network meta-analysis. *Critical Care* 2015; 19.

753. Wang J, Meng XH, Guo ZM, Wu YH, Zhao JG. Interventions for Treating Displaced Midshaft Clavicular Fractures A Bayesian Network Meta-Analysis of Randomized Controlled Trials. *Medicine* 2015; 94(11).

754. Wang J, Xia SJ, Liu ZH, et al. Inguinal and subinguinal micro-varicocelectomy, the optimal surgical management of varicocele: a meta-analysis. *Asian Journal of Andrology* 2015; 17(1): 74-80.

755. Wang L, Baser O, Kutikova L, Page JH, Barron R. The impact of primary prophylaxis with granulocyte colony-stimulating factors on febrile neutropenia during chemotherapy: a systematic review and meta-analysis of randomized controlled trials. *Supportive Care in Cancer* 2015.

756. Wang ZY, Qiao D, Lu YH, et al. Systematic Literature Review and Network Meta-Analysis Comparing Bone-Targeted Agents for the Prevention of Skeletal-Related Events in Cancer Patients With Bone Metastasis. *Oncologist* 2015; 20(4): 440-9.

757. Wilhelmus Kirk R. Antiviral treatment and other therapeutic interventions for herpes simplex virus epithelial keratitis. *Cochrane Database of Systematic Reviews*, 2015. http://onlinelibrary.wiley.com/doi/10.1002/14651858.CD002898.pub5/abstract (accessed.

758. Wyles CC, Jimenez-Almonte JH, Murad MH, et al. There Are No Differences in Short- to Mid-term Survivorship Among Total Hip-bearing Surface Options: A Network Meta-analysis. *Clinical Orthopaedics and Related Research* 2015; 473(6): 2031-41.

759. Yan M, Kumachev A, Siu LL, Chan KKW. Chemoradiotherapy regimens for locoregionally advanced nasopharyngeal carcinoma: A Bayesian network meta-analysis. *European Journal of Cancer* 2015.

760. Yao M, Yang L, Wang J, et al. Neurological Recovery and Antioxidant Effects of Curcumin for Spinal Cord Injury in the Rat: A Network Meta-Analysis and Systematic Review. *Journal of Neurotrauma* 2015; 32(6): 381-91.

761. Yildiz A, Nikodem M, Vieta E, Correll CU, Baldessarini RJ. A network meta-analysis on comparative efficacy and all-cause discontinuation of antimanic treatments in acute bipolar mania. *Psychological Medicine* 2015; 45(2): 299-317.

762. Yu QY, Zhu ZL, Liu Y, Zhang J, Li K. Efficacy and Safety of HER2-Targeted Agents for Breast Cancer with HER2-Overexpression: A Network Meta-Analysis. *Plos One* 2015; 10(5).

763. Zeng C, Li H, Yang T, et al. Electrical stimulation for pain relief in knee osteoarthritis: systematic review and network meta-analysis. *Osteoarthritis and Cartilage* 2015; 23(2): 189-202.

764. Zeng C, Wei J, Li H, et al. Comparison between 200 mg QD and 100 mg BID oral celecoxib in the treatment of knee or hip osteoarthritis. *Scientific Reports* 2015; 5.

765. Zhang XL, JiWang W, Wang WJ, Cao N. Effectiveness and Safety of Controlled Venous Pressure in Liver Surgery: A Systematic Review and Network Meta-Analysis. *Biomed Research International* 2015.

766. Zhang XY, Shuai J, Li LP. Vision and Relevant Risk Factor Interventions for Preventing Falls among Older People: A Network Meta-analysis. *Scientific Reports* 2015; 5.

767. Zhou XY, Hetrick SE, Cuijpers P, et al. Comparative efficacy and acceptability of psychotherapies for depression in children and adolescents: A systematic review and network meta-analysis. *World Psychiatry* 2015; 14(2): 207-22.

768. Zhou XY, Ravindran AV, Qin B, et al. Comparative Efficacy, Acceptability, and Tolerability of Augmentation Agents in Treatment-Resistant Depression: Systematic Review and Network Meta-Analysis. *Journal of Clinical Psychiatry* 2015; 76(4): E487-.

769. Zhu GQ, Shi KQ, Huang S, et al. Network Meta-Analysis of Randomized Controlled Trials: Efficacy and Safety of UDCA-Based Therapies in Primary Biliary Cirrhosis. *Medicine* 2015; 94(11).

770. Zhu GQ, Shi KQ, Huang S, et al. Systematic review with network meta-analysis: the comparative effectiveness and safety of interventions in patients with overt hepatic encephalopathy. *Alimentary Pharmacology & Therapeutics* 2015; 41(7): 624-35.

771. Zhu GQ, You J, Shi KQ, et al. Systematic Review With Network Meta-Analysis Adjuvant Chemotherapy for Resected Colorectal Liver Metastases. *Medicine* 2015; 94(1).
