# Supplementary material for: The Global Research Collaboration of Network Meta-Analysis: A Social Network Analysis
Source: PLoS One. 2016 Sep 29;11(9):e0163239. doi: 10.1371/journal.pone.0163239 (PMC5042468; doi:10.1371/journal.pone.0163239)

**S3 Appendix. Additional Figures.**

Figure A. The number of published NMAs from 1997 to 2015


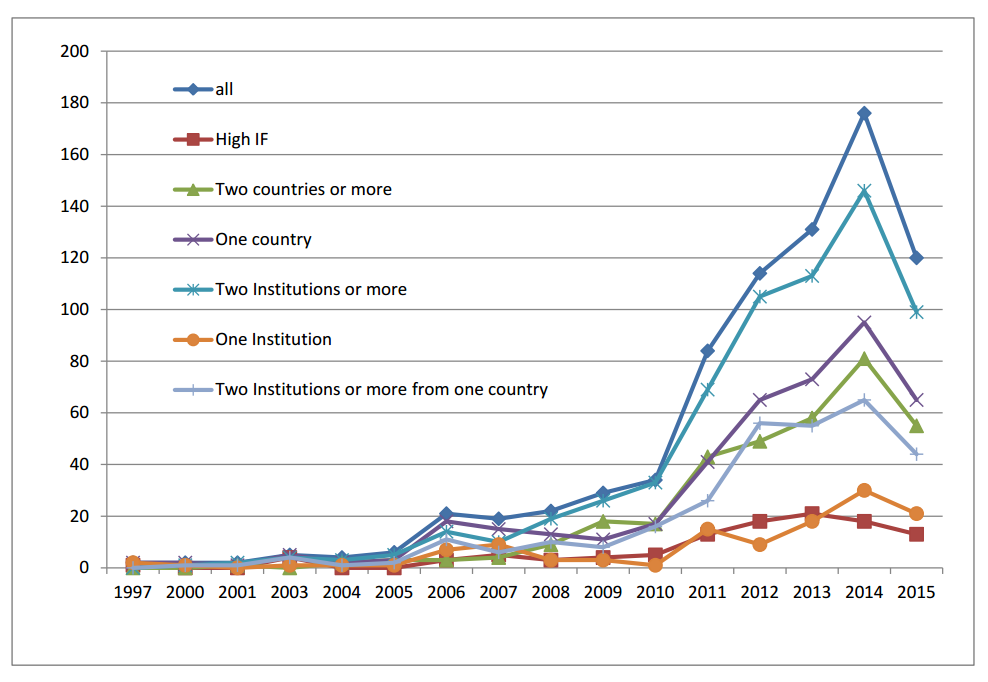
IF: impact factor journal (> 10, ISI Web of Science 2014).

Figure B. The average author, institution, and country number (NO.) per year in all journals or high impact factor journals.


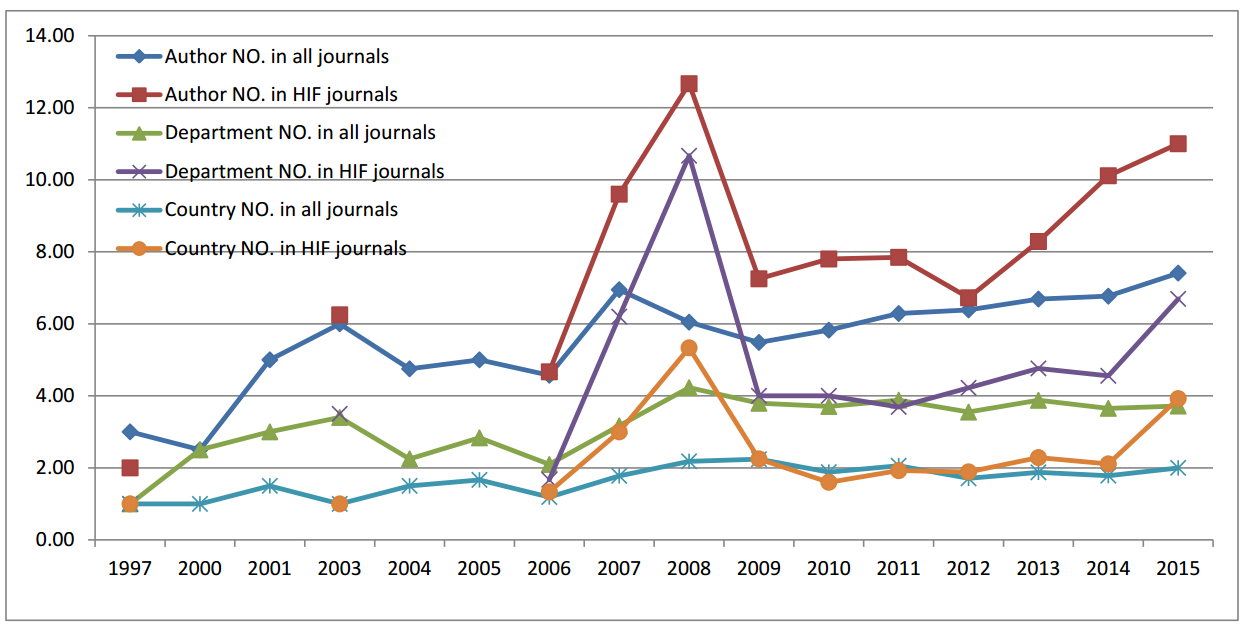


Figure C. The number of authors, institutions, or countries per NMA


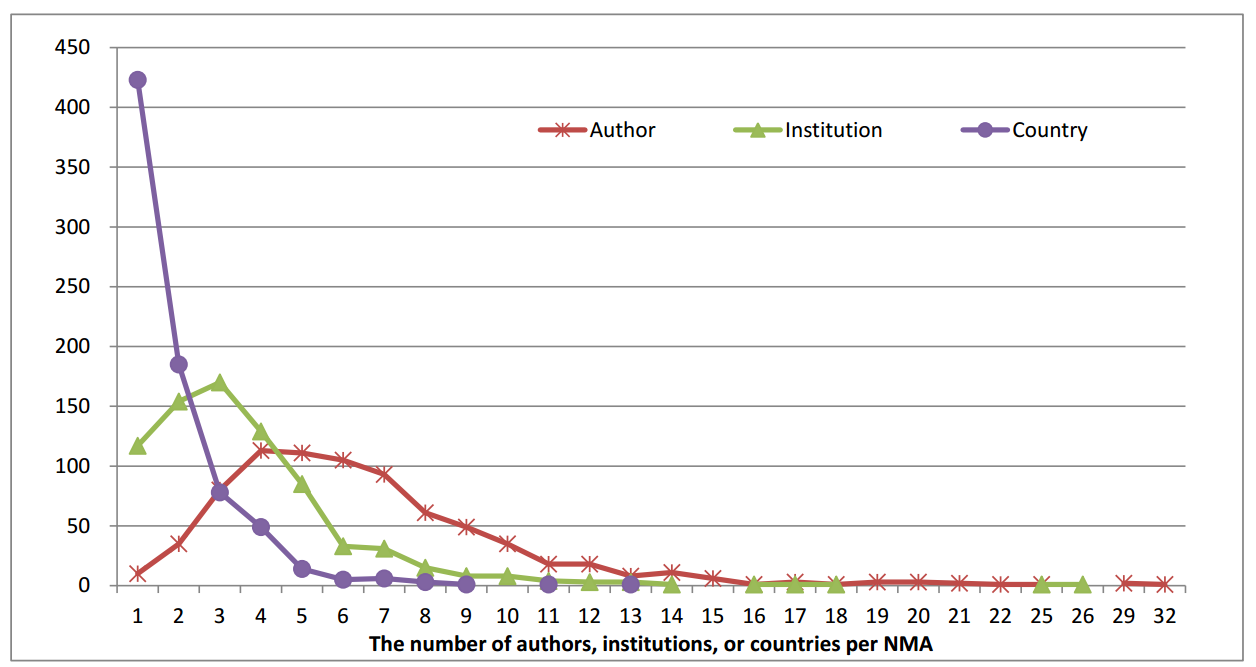


Figure D. The collaboration times at most among authors and institutions in all journals or high impact factor journals.


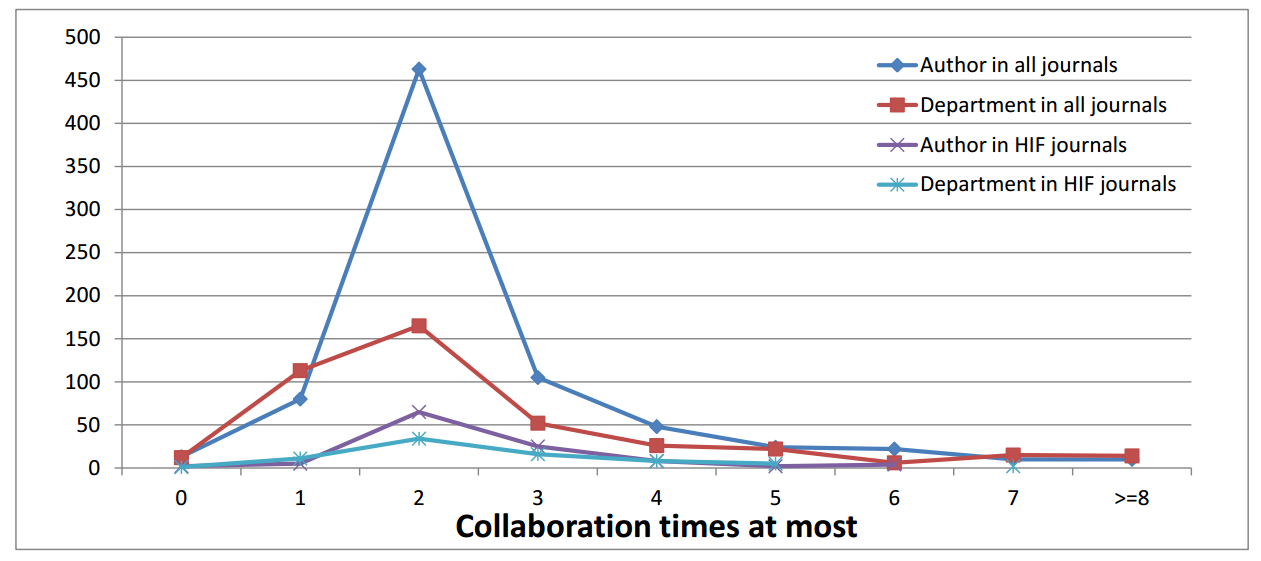


Figure E. Main clusters of authors applying a threshold of five or more NMAs signed in co-authorship in all journals


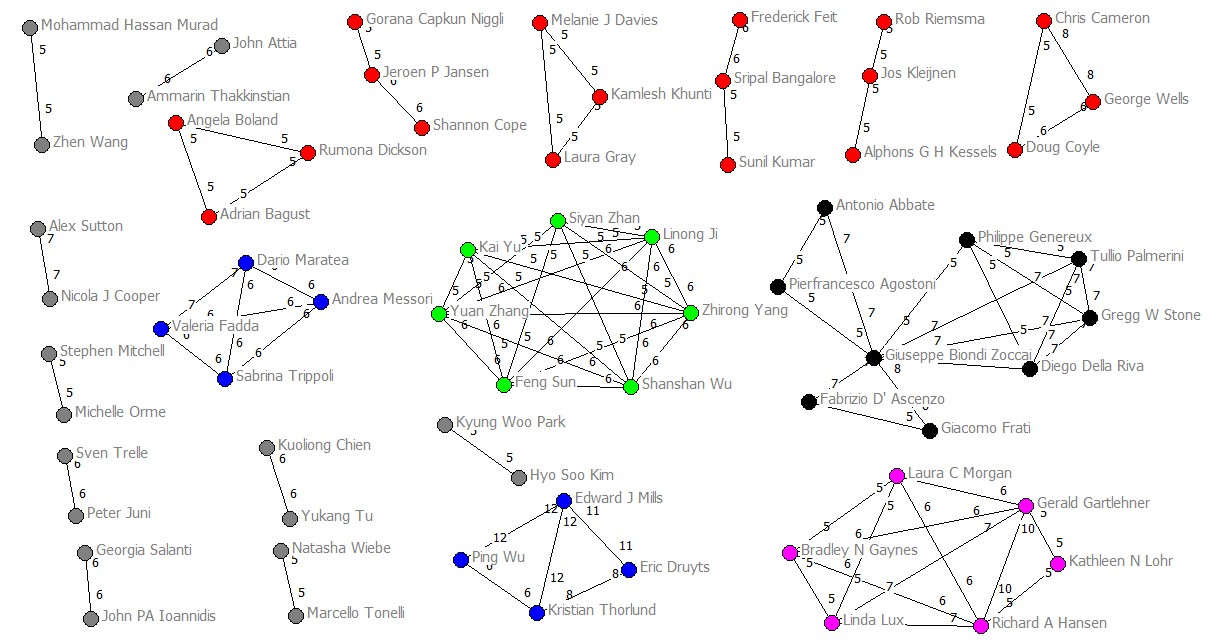


Figure F. Main clusters of institutions applying a threshold of five or more NMAs signed in co-authorship in all journals


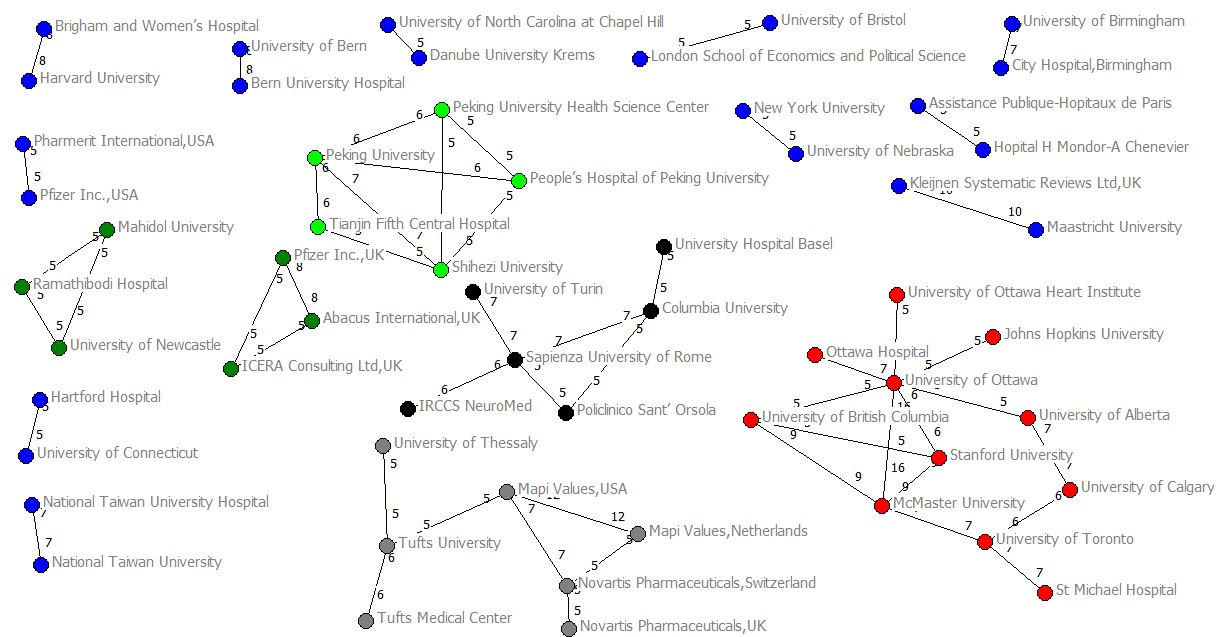

Supplement: S3 Appendix — (DOCX) [file pone.0163239.s003.docx]
