# Supplementary material for: The Global Research Collaboration of Network Meta-Analysis: A Social Network Analysis
Source: PLoS One. 2016 Sep 29;11(9):e0163239. doi: 10.1371/journal.pone.0163239 (PMC5042468; doi:10.1371/journal.pone.0163239)
Supplement: S4 Appendix — (DOCX) [file pone.0163239.s004.docx]

**Appendix S4. Additional Tables**

**Additional Table A. Ranking of authors (top 20) by centrality measure.**

| Freeman's degree | Closeness | Freeman betweenness |
| --- | --- | --- |
| Giuseppe BiondiZoccai(144)  Gregg W Stone(133)  Diego Della Riva(90)  TullioPalmerini(90)  HyoSoo Kim(82)  Edward J Mills(80)  Peter Juni(79)  Gerald Gartlehner(74)  Richard A Hansen(74)  Martin B Leon(73)  Philippe Genereux(67)  Linda Lux(62)  Kyung Woo Park(62)  Jeroen P Jansen(61)  Christoph Stettler(61)  Giacomo Frati(60)  George Wells(58)  Georgia Salanti(57)  AdnanKastrati(57)  Fabrizio D' Ascenzo(57) | Gordon Guyatt (0.42)  Graziella Filippini (0.42)  Chris Cameron (0.42)  Nicole Skoetz (0.42)  Robin Christensen (0.42)  Nicky Welton (0.42)  Edward J Mills (0.42)  IsabellaGhement (0.42)  Milo A Puhan (0.42)  Ping Wu (0.42)  Jeroen P Jansen (0.42)  George Wells (0.42)  Elizabeth Tanjong Ghogomu (0.42)  Peter Tugwell (0.42)  Jasvinder A Singh (0.42)  Rachelle Buchbinder (0.42)  Kristian Thorlund (0.42)  Sven Trelle (0.42)  Pieter Drost (0.42)  Gerbenter Riet(0.42)  Peter Juni(0.42)  Georgia Salanti(0.42)  Steve Kanters(0.42)  Sonal Singh(0.42)  OghenowedeEyawo(0.42)  Matthias Briel(0.42)  Jason W Busse(0.42) | Gordon Guyatt (11.77)  Georgia Salanti (10.59)  Graziella Filippini (8.41)  Milo A Puhan (7.70)  NickyWelton (7.14)  Peter Juni (6.86)  Jeroen P Jansen (6.46)  John PA Ioannidis (6.37)  Gerbenter Riet(6.36)  Thomas A Trikalinos(6.15)  Robin Christensen (5.69)  Alex Sutton (5.60)  Isabella Ghement (5.06)  Sven Trelle (4.66)  Chris Cameron (4.35)  Pieter Drost (4.20)  Nicole Skoetz (4.19)  Benjamin Djulbegovic (4.13)  Jos Kleijnen (3.90)  Keith Wheatley (3.81) |

**Additional Table B. Ranking of institutions (top 20) by centrality measure.**

| Freeman's degree | Closeness | Freeman betweenness |
| --- | --- | --- |
| Columbia University(127)  University of Ottawa(102)  McMaster University(94)  Sapienza University of Rome(93)  Technische Universitat Munchen(73)  Harvard University(66)  University of Birmingham(59)  University of Toronto(58)  University Hospital Basel(58)  University of Bristol(53)  University of Bern(52)  University of Turin(51)  MapiValues, USA(49)  Bern University Hospital(47)  Brigham and Women’s Hospital(46)  Imperial College London(44)  Assistance Publique-Hopitaux de Paris(42)  Tufts University(42)  University of British Columbia(41)  Stanford University(41) | Columbia University (14.33)  Harvard University (14.21)  University of Birmingham (14.05)  University of Bristol (14.04)  McMaster University (14.04)  University of Ottawa (14.02)  Technische Universitat Munchen (13.90)  University Hospital Basel (13.88)  Pfizer Inc., USA (13.83)  Tufts University (13.82)  University of British Columbia (13.80)  Imperial College London (13.78)  Leiden University Medical Center (13.76)  Stanford University (13.75)  Sapienza University of Rome (13.73)  University of Toronto (13.64)  University of Bern (13.63)  Brigham and Women’s Hospital (13.61)  Brown University (13.60)  Copenhagen University Hospital (13.60) | Columbia University(12.67)  University of Birmingham(8.29)  Harvard University(8.24)  University of Bristol(7.47)  McMaster University(6.41)  Technische Universitat Munchen(6.33)  University of Ottawa(5.87)  Sapienza University of Rome(5.53)  Pfizer Inc., USA(4.46)  Boehringer Ingelheim Ltd, Germany(3.75)  Imperial College London(3.73)  University of Toronto(3.34)  Queens University(3.06)  Mapi Values, USA(2.80)  Tufts University(2.62)  AstraZeneca Ltd, UK(2.46)  The Chinese University of Hong Kong(2.33)  Bristol-Myers Squibb, USA(2.30)  Leiden University Medical Center(2.26)  University College London(2.08) |

**Additional Table C. Ranking of countries (top 20) by centrality measure.**

| Freeman's degree | Closeness | Freeman betweenness |
| --- | --- | --- |
| UK(386)  USA(376)  Netherlands(209)  Italy(173)  Germany(172)  Canada(156)  Switzerland(135)  France(124)  Denmark( 91)  Belgium( 87)  Spain( 83)  Australia( 73)  Korea( 54)  Greece( 54)  Brazil( 45)  China( 43)  Austria(35)  Sweden( 35)  Poland( 29)  Czech Republic( 24) | UK(47.37)  USA(46.75)  Italy(43.37)  Germany(43.37)  Canada(42.86)  Australia(41.38)  Netherlands(41.38)  France(40.91)  Spain(40.45)  Switzerland(40.45)  Korea(40)  Denmark(40)  Belgium(40)  Brazil(39.13)  Poland(39.13)  Greece(38.71)  Sweden(38.71)  China(38.30)  Austria(37.90)  Czech Republic(37.50) | UK(16.09)  USA(13.24)  Canada(8.20)  Sweden(5.68)  Italy(5.46)  Germany(3.60)  Belgium(1.92)  Denmark(1.62)  Australia(1.51)  France(1.50)  Poland(1.29)  Brazil(1.18)  Netherlands(1.11)  Spain(1.10)  Switzerland(1.09)  Austria(0.73)  Korea(0.69)  China(0.61)  India(0.58)  Greece(0.47) |
